# Tetrandrine, a Major Alkaloid From *Stephaniae Tetrandrae Radix*, Ameliorates Non‐Alcoholic Fatty Liver Disease in Zebrafish via the PI3K/AKT/STAT3 Pathway

**DOI:** 10.1002/fsn3.71814

**Published:** 2026-05-12

**Authors:** Peng Sun, Jun Wu, Kai Ma, Xiaoli Wei, Jing Ma, Wenhao Han, Xiaoyu Zhu, Xinyu Zhang, Xi Wang, Tangnuer Tuersunniyazi, Wei Gong, Hong Lin

**Affiliations:** ^1^ Ningxia Hui Autonomous Region Institute of Medical Sciences Yinchuan China; ^2^ Science and Technology Center Ningxia Medical University Yinchuan China; ^3^ Public Health School Ningxia Medical University Yinchuan China; ^4^ School of Pharmacy Ningxia Medical University Yinchuan China; ^5^ School of Clinical Medicine Ningxia Medical University Yinchuan China; ^6^ School of Medical Information and Engineering Ningxia Medical University Yinchuan China; ^7^ School of Nursing Ningxia Medical University Yinchuan China

**Keywords:** alkaloids, molecular dynamics simulation, NAFLD, network pharmacology, tetrandrine, zebrafish model

## Abstract

Non‐alcoholic fatty liver disease (NAFLD) is a common metabolic liver disorder with limited effective pharmacological treatments. Tetrandrine (Tet), a major bioactive alkaloid derived from *Stephaniae Tetrandrae Radix*, has shown anti‐inflammatory and metabolic regulatory potential. In this study, we systematically investigated the therapeutic effects and underlying mechanisms of STR alkaloids, with a focus on Tet in NAFLD. Network pharmacology identified active alkaloids and 194 overlapping targets associated with NAFLD, and protein–protein interaction analysis highlighted TP53, EGFR, STAT3, AKT1, and TNF as key hub genes. Functional enrichment analyses indicated that the PI3K/AKT, MAPK, and FoxO signaling pathways were significantly involved, with PI3K/AKT emerging as the central signaling pathway. Molecular docking and molecular dynamics simulations demonstrated stable binding interactions between Tet and STAT3 as well as TNF. Mendelian randomization analysis further suggested a causal relationship between elevated STAT3 expression and increased NAFLD risk. Experimental validation in a thioacetamide‐induced zebrafish NAFLD model showed that Tet significantly reduced hepatic lipid accumulation and decreased serum TG, TC, ALT, and AST levels. Mechanistically, Tet activated PI3K/AKT/STAT3 signaling while inhibiting TP53 and Bax. External validation using GEO datasets supported AKT1 and EGFR as important regulatory genes. Overall, these findings demonstrated that Tet ameliorates NAFLD by modulating lipid metabolism and apoptosis through activation of the PI3K/AKT/STAT3 signaling pathway, providing integrated computational, genetic, and experimental evidence for its potential as a therapeutic agent.

## Graphical abstract

Tet regulates lipid metabolism and cell apoptosis to alleviate NAFLD. Integrated network pharmacology, Mendelian randomization, and experimental validation demonstrate that Tet activates the PI3K/AKT/STAT3 signaling axis, suppresses apoptosis, reduces hepatic lipid accumulation, and improves biochemical liver injury markers, supporting its potential as a therapeutic candidate for NAFLD.

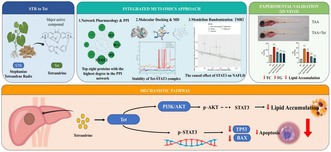

## 1 Introduction

Non‐alcoholic fatty liver disease (NAFLD) has emerged as a major global health concern, with its incidence rising steadily in parallel with increasing rates of obesity, sedentary lifestyles, unhealthy dietary habits, and aging populations (Le et al. 2023). NAFLD is characterized by excessive triglyceride accumulation in hepatocytes and includes a spectrum of disorders ranging from simple steatosis to non‐alcoholic steatohepatitis (NASH), which may progress to fibrosis, cirrhosis, or hepatocellular carcinoma (Brunt et al. 2015). Beyond its hepatic manifestations, NAFLD is closely associated with systemic metabolic disorders, including obesity, type 2 diabetes mellitus (T2DM), dyslipidemia, insulin resistance, and metabolic syndrome. Despite its increasing prevalence, the underlying pathophysiological mechanisms of NAFLD remain incompletely understood, and no specific pharmacological treatments have been approved for clinical use.

Traditional Chinese medicine (TCM), characterized by multi‐component and multi‐target synergistic actions, offers promising opportunities for developing new NAFLD therapies. Among various TCM herbs, Stephaniae Tetrandrae Radix (STR), derived from the roots of Stephania tetrandra S. Moore, has a long history of clinical use for rheumatic diseases, cardiovascular disorders, and malignancies (Jiang et al. 2020). Modern phytochemical analyses using ultra‐fast liquid chromatography–quadrupole time‐of‐flight tandem mass spectrometry (UPLC‐Q‐TOF‐MS/MS) have identified 393 isoquinoline alkaloids across 20 structural classes in STR, highlighting its remarkable chemical diversity (Chen et al. 2020). Among its bioactive constituents, alkaloids such as tetrandrine (Tet), stephanine, and betaine exhibit anti‐inflammatory, antifibrotic, and hypoglycemic properties (Wang et al. 2020; Zhang, Qi, et al. 2020).

Tetrandrine, a bioactive bisbenzyl isoquinoline alkaloid, has demonstrated significant therapeutic potential in multiple disease contexts. Tet exerts potent antitumor effects through modulation of signaling pathways such as TAK1 and NF‐κB and has been shown to regulate hepatic stellate cell activation (X. Li et al. 2016; Liu et al. 2016). Additionally, Tet has glucose‐lowering effects in experimental models of diabetes, suggesting its potential to mitigate metabolic derangements associated with NAFLD (Hsu et al. 2004; Shan et al. 2013). Given the bidirectional relationship between NAFLD and diabetes, elucidating the molecular mechanisms of STR alkaloids—particularly Tet—could provide insight into new therapeutic strategies (Alexopoulos et al. 2021; Padda et al. 2021; Zoppini et al. 2014).

To validate candidate compounds and explore their in vivo metabolic effects, appropriate experimental models are essential. Zebrafish, as a classic model organism for metabolic liver disease research, offers unique advantages in the study of NAFLD (de Oliveira et al. 2019; Goessling and Sadler 2015; Shimizu et al. 2023). Key metabolic processes such as lipid absorption, fatty acid synthesis and breakdown, and insulin signaling are highly conserved between zebrafish and mammals (Hölttä‐Vuori et al. 2010; Ou‐Yang et al. 2025). Moreover, zebrafish share over 70% genetic homology with humans (Howe et al. 2013), and signaling pathways closely associated with NAFLD, including PI3K/AKT and MAPK, are highly conserved between the two species (Yang et al. 2025), making zebrafish an effective tool for investigating early pathological mechanisms of NAFLD and in vivo drug effects. In addition, the transparency of zebrafish embryos, their rapid development, and high reproductive capacity facilitate fast NAFLD modeling and high‐throughput drug screening (Chang et al. 2023).

In this study, we aimed to systematically investigate the therapeutic potential and molecular mechanisms of STR alkaloids, focusing on Tet, in the context of NAFLD. First, we analyzed the active ingredients of STR, predicted their molecular targets, and constructed a drug‐target network using network pharmacology. Next, we employed molecular docking and molecular dynamics (MD) simulations to evaluate the interactions and binding affinities between active compounds and their predicted targets, identifying the most dominant bioactive components. We further conducted Mendelian randomization (MR) analyses to explore causal relationships between key molecular targets and NAFLD progression. To validate our findings experimentally, we examined the effects of Tet in zebrafish larval models of NAFLD and cross‐verified key targets using the Gene Expression Omnibus (GEO) database. This study provided both theoretical and experimental evidence supporting the therapeutic potential of STR alkaloids in NAFLD, elucidates the pharmacological mechanisms of Tet, and offers valuable insights for the development of innovative clinical strategies leveraging the multi‐component and multi‐target advantages of traditional Chinese medicine.

## 2 Materials and Methods

### 2.1 Screening of Active Compounds and Potential Targets of STR Alkaloid

Active compounds of STR were searched in the TCMSP database, using oral bioavailability (OB) ≥ 20% and drug‐likeness (DL) ≥ 0.10 as selection criteria. The keywords “N‐Methylflindersine”, “Tetrandrine”, “Betaine”, “Magnoflorine” and “Stepharine” were queried in the TCMSP (https://tcmsp‐e.com/tcmsp.php), DrugBank (https://go.drugbank.com/), TTD (https://db.idrblab.net/ttd), SymMap (http://www.symmap.org/), ChEMBL (https://www.ebi.ac.uk/chembl/), PharmMapper (http://www.lilab‐ecust.cn/pharmmapper/), and SwissTargetPrediction (http://swisstargetprediction.ch/) databases to identify potential targets.

### 2.2 Prediction and Screening of NAFLD Targets

“Non‐alcoholic fatty liver Diseases” as keywords in GeneCards (https://www.genecards.org), OMIM (https://www.omim.org/), DisGeNET (https://www.disgenet.org/), UniProt (https://www.uniprot.org/), DrugBank, TTD providing the database query for disease targets. Disease targets were screened based on “ *Homo sapiens* ”. In GeneCards database, a relevance score greater than the median was used as the screening criterion. A score value greater than one‐third of the maximum value in the DisGeNET database was used as a filtering criterion (Sun et al. 2025; Wang et al. 2024).

### 2.3 PPI Network of Therapeutic Targets Between STR Alkaloids and NAFLD

Drug and disease targets were standardized to gene symbols, and duplicate entries were removed. These genes were analyzed using the Venny online tool (https://bioinfogp.cnb.csic.es/tools/venny/) to identify intersecting genes, which were then imported into the STRING database (https://cn.string‐db.org/) with species set to “ *Homo sapiens* ” and confidence level ≥ 0.700 (Huang et al. 2026). The resulting data were exported and analyzed topologically using Centiscape 2.2 and CytoNCA plugins in Cytoscape 3.9.1 to construct a protein–protein interaction (PPI) network.

### 2.4 GO Function and KEGG Pathway Enrichment Analysis

Intersection drug and NAFLD target genes were uploaded to the DAVID platform (https://david.ncifcrf.gov/) for Gene Ontology (GO) analysis, which included biological processes (BP), cellular components (CC), and molecular functions (MF). Pathway enrichment was conducted using the Kyoto Encyclopedia of Genes and Genomes (KEGG, https://www.kegg.jp/). The top 10 BP, CC, and MF terms, as well as the top 20 KEGG pathways, were selected based on *p*‐values. KEGG pathway visualization was performed on the Bioinformatics platform (http://www.bioinformatics.com.cn). Additionally, pathways related to Metabolism, Genetic Information Processing, Environmental Information Processing, Cellular Processes, and Organismal Systems were identified using the KEGG Mapper tool (Niu et al. 2024).

### 2.5 MCODE Cluster Analysis

The MCODE algorithm in Metascape (https://metascape.org/) was used to identify functional modules of the common targets. By using molecular complex detection technology, protein interactions were clustered into gene clusters with similar functions. MCODE analysis was performed with parameters: Min Overlap = 3, *p*‐Value Cutoff = 0.05 and Min Enrichment = 1.5 to identify protein complexes involved in the therapeutic effects of STR alkaloids.

### 2.6 Network Construction of Active Ingredient‐Target‐Pathway Interaction

Active compounds, core targets, and the top 20 enriched pathways from KEGG were imported into Cytoscape 3.9.1 to construct the active ingredient‐target‐pathway network. Topological parameters were analyzed to identify the key active ingredients and core targets responsible for the pharmacological effects.

### 2.7 STR Alkaloids ADMET Evaluation

SwissADME (http://www.swissadme.ch/index.php) and ADMETLab 2.0 (https://admetmesh.scbdd.com/) were used to evaluate the absorption, distribution, metabolism, excretion, and toxicity (ADMET) properties of the STR alkaloids. The compounds were assessed for physicochemical properties according to Lipinski's rules (molecular weight < 500 g/mol, topological polar surface area < 140, octanol–water partition coefficient ≤ 4.15, hydrogen bond acceptors < 10, hydrogen bond donors ≤ 5). Toxicity was evaluated using five parameters: hERG inhibition, human hepatotoxicity, carcinogenicity, AMES toxicity, and eye corrosion.

### 2.8 Molecular Docking

Molecular docking was used to predict the therapeutic potential of active compounds by analyzing their binding energies with key target proteins. Five active ingredients and eight core proteins were docked for 40 times in a one‐by‐one way. In the TCMSP database, download 5 active ingredients in. MOL2 format as small molecules. Eight macromolecular core proteins were searched in the RCSB database (https://www.rcsb.org/), “ *Homo sapiens* ” was selected, and the “*Unique Ligands*” were recorded. The downloaded macromolecular proteins were imported into PyMOL software to remove ligands, and then exported into PDBQT format. The AutoDock Tools software was used to remove water and hydrotreat small molecules and macromolecular proteins, and the charge number of small molecules was checked. A crystal structure docking grid box was constructed for each target, and the interaction between molecules and primary ligands and molecules was compared, allowing the binding energy of each active compound in the docking concept to be minimum, to achieve molecular docking. In order to ensure the accuracy of the data, the number of docking runs was selected to be 50 times, and finally PyMOL software was used to visualize the docking results.

### 2.9 Molecular Dynamics Simulations

Molecular dynamics (MD) simulations were conducted using the Desmond v53011 module in the Schrödinger suite. The protein‐ligand complex was initially processed with the Protein Preparation module under default parameters, including assignment of bond orders, addition of hydrogen atoms, removal of crystallographic water molecules, and restrained energy minimization. The prepared complex was subsequently embedded in an explicit solvent environment using the TIP3P water model and the OPLS3 force field within the System Builder module. An orthorhombic simulation box was defined based on a buffer‐distance criterion. The system was neutralized with appropriate counter ions, and Na^+^ions were added to mimic physiological ionic strength. Prior to the production phase, the system underwent energy minimization to eliminate unfavorable contacts. MD simulations were then performed under default conditions (Kato et al. 2021). Post‐simulation analyses were carried out using the Simulation Interactions Diagram tool.

Structural stability of the protein‐ligand complex was evaluated by calculating the root mean square deviation (RMSD) of backbone atoms. Protein compactness was assessed using the radius of gyration (Rg), while residue‐level flexibility was analyzed through root mean square fluctuation (RMSF). In addition, the number of intermolecular hydrogen bonds between the protein and ligand was monitored throughout the simulation to assess interaction persistence.

### 2.10 Mendelian Randomization Analysis of Key Genes and NAFLD

#### 2.10.1 Study Design

A two‐sample MR approach was used to explore the causal relationship between key genes and NAFLD. MR analysis helps address confounding biases in observational studies by utilizing genetic variants to infer causal effects.

#### 2.10.2 Data Source

SNP data for eight candidate genes (TP53, STAT3, EGFR, AKT1, TNF, CTNNB1, BCL2, and INS) and NAFLD were obtained from publicly available genome‐wide association studies (GWAS) data from European populations. Sample sizes and SNP details were provided in Table 1.

**TABLE 1:** A summary of the data for MR analysis.

Name	Dataset	SNPs in GWAS	Sample size	European	Year	Author
TP53	eqtl‐a‐ENSG00000141510	18,457	31,684	European	2018	Vosa U
STAT3	eqtl‐a‐ENSG00000168610	16,677	31,470	European	2018	Vosa U
EGFR	prot‐a‐909	10,534,735	3301	European	2018	Sun BB
AKT1	eqtl‐a‐ENSG00000142208	20,441	30,721	European	2018	Vosa U
TNF	eqtl‐a‐ENSG00000232810	33,864	14,263	European	2018	Vosa U
CTNNB1	eqtl‐a‐ENSG00000168036	18,730	31,684	European	2018	Vosa U
BCL2	eqtl‐a‐ENSG00000171791	18,388	31,644	European	2018	Vosa U
INS	ebi‐a‐GCST90025989	4,231,359	435,516	European	2021	Barton AR
NAFLD	finn‐b‐NAFLD	16,380,466	2,178,987	European	2021	NA

#### 2.10.3 Instrumental Variable Selection

Instrumental variables (IVs) were selected based on GWAS summaries and literature reviews, with SNPs having *p*‐values < 1 × 10^−5^. SNPs with strong linkage disequilibrium (*r* ^2^ < 0.01) and a distance of > 1000 kb were excluded. The “mv_harmonise_data” function from the “TwoSampleMR” package was used to harmonize alleles, and “mv_lasso_feature_selection” was applied to remove collinear variables, ensuring the independence of IVs. These stringent criteria ensured the effectiveness of the SNPs as instrumental variables for the subsequent MR analysis.

#### 2.10.4 MR Analysis

MR analysis was performed using the “TwoSampleMR” (version 0.6.8) and “MR‐PRESSO” (version 1.0) packages. Methods including inverse variance weighting (IVW), MR‐Egger regression, weighted median (WM), and weighted models were applied to evaluate causality; the IVW method was the primary approach. Sensitivity analyses, including heterogeneity tests, horizontal pleiotropy assessments, and one‐by‐one exclusion, were conducted to ensure robustness. *p*‐values < 0.05 were considered statistically significant. All analyses were performed using R 4.2.3.

### 2.11 The Zebrafish Experiment

#### 2.11.1 Experimental Animal Husbandry

Zebrafish stocks (wildtype, CZRC ID: CZ1) were obtained from the China Zebrafish Resource Center (CZRC) and maintained at 28°C in a circulating aquarium facility with a 12:12 h light:dark cycle. The fish were housed in tanks with a pH of 7.4. Embryos were obtained via natural spawning and cultured in embryo medium at 28.5°C in culture dishes within an incubator. Embryos were used at 3–5 days post‐fertilization (dpf).

#### 2.11.2 Drugs, Reagents, and Instruments

TAA (Shanghai Sangon Biotech, CAS: 62–55‐5), Tet (Beijing Solaibao Company, CAS: 518–34‐3), LY294002 (Shanghai MedChemExpress, CAS: 154447–36‐6), Oil Red O (Wuhan Servicebio Company, Lot number: G1015‐100ML), and triglyceride (TG) and total cholesterol (TC) kits (Jiancheng Institute of Biological Engineering, Nanjing, Batch numbers: A110‐1‐1 and A111‐1‐1, respectively) were used in this study. Zebrafish were maintained in a standard breeding system under controlled laboratory conditions (Shanghai Haisheng Co. Ltd.). The microscope used was an Olympus Model BX61 (Japan). The zebrafish breeding system from Beijing Aisen Biotechnology Co. Ltd. (Model: ESEN‐AW‐S1) and a constant‐temperature biochemical incubator from Guangzhou Huruiming Instrument Co. Ltd. (Model: LRH‐250Z) were also employed. Real‐time PCR was performed using a Bio‐Rad CFX96 system (USA). An Ultramicro‐ultraviolet spectrophotometer (Thermo Scientific, USA, Model: NanoDrop 2000) was used for measurements.

#### 2.11.3 Modeling and Administration of NAFLD in Zebrafish

Following established protocols, 3‐day‐old zebrafish larvae were exposed to 0.4 mg/mL thioacetamide (TAA) for 48 h in 12‐well plates, with the solution replaced every 24 h (Gao et al. 2024; H. Zhang et al. 2024). After 24 h of modeling, the treatment groups were divided into the following: low‐dose Tet (0.625 μM), medium‐dose Tet (1.25 μM), and high‐dose Tet (2.5 μM) (Orencio et al. 2026; Zhang, Wang, et al. 2020). The normal control group was treated with culture water. At the end of the experiment, Oil Red O staining and pathological observation were conducted according to established methods. Biochemical parameters, including TC and TG, were measured following the respective kit instructions.

#### 2.11.4 Whole Fish Oil Red O Staining

The juvenile zebrafish were fixed overnight in 4% paraformaldehyde (PFA) and washed three times with PBS. The fish were then immersed in 60% isopropyl alcohol for 30 min. After discarding the alcohol, Oil Red O solution (prepared by mixing ddH2O at a 3:2 volume ratio) was applied for 3 h. The staining solution was discarded, and the fish were rinsed with 60% isopropyl alcohol before adding ddH_2_O. Finally, fat droplets in the liver tissue were observed and photographed using a microscope.

#### 2.11.5 TG and TC Assays

Juvenile zebrafish (*n* = 20 per group) were washed three times with PBS, and their body mass was accurately recorded. For each sample, 9 times the volume of pre‐cooled normal saline was added (weight in grams: volume in mL = 1:9). The fish were then disrupted using an ultrasonic homogenizer in an ice‐water bath. After homogenization, the samples were centrifuged at 4°C for 10 min at 2500 RPM, and the supernatant was collected. The concentrations of TG and TC were measured using commercial assay kits (Nanjing, China), with absorbance measured at 500 nm using an enzyme‐labeled apparatus. Each sample was tested in triplicate.

#### 2.11.6 Real‐Time Fluorescence Quantitative PCR (RT‐qPCR)

The genes analyzed in this study include P53, BCL‐2, BAX, PI3K, AKT and STAT3. Primer sequences were designed using Primer 5 software, and primer specificity was verified using the online tool Primer‐BLAST. The sequences are shown in Table 2. Primers were synthesized by Sangon Biotech (Shanghai) Co. Ltd. Zebrafish tissues were homogenized in a centrifuge tube with RNA lysate pre‐added (4°C). After homogenization, the samples were centrifuged at 4°C at 12,000 rcf for 10 min, and the supernatant was collected for RNA extraction. Total RNA extraction, reverse transcription, and RT‐qPCR were performed strictly according to the kit instructions (Tiangen Biochemical Technology Co. Ltd., China). GAPDH was used as the internal reference gene. Each sample was analyzed in triplicate, and gene expression levels were calculated using the 2^−ΔΔCt method.

**TABLE 2:** The primer sequence of target gene.

Gene	Primer forward (5′‐3′)	Primer reverse (5′‐3′)
P53	GCTTGTCACAGGGGTCATTT	ACAAAGGTCCCAGTGGAGTG
BCL2‐F	TCACTCGTTCAGACCCTCAT	ACGCTTTCCACGCACAT
BAX	GGCTATTTCAACCAGGGTTCC	TGCGAATCACCAATGCTGT
PI3K	GAGATTTTCTCGGCCCTGGCT	ACTCTTCCCATCTGTGTGAGGC
AKT	GGCTATAAGGAGCGACCGCA	GGTGCGCTCAATGACAGTGG
STAT3	CCCTGGGACTAACTCTGGCA	AGAGGTCCTGGATTGGCCTC
GAPDH	TGCTGGTATTGCTCTCAACG	GCCATCAGGTCACATACACG

#### 2.11.7 Investigation of LY294002 on Tet‐Mediated PI3K/AKT/STAT3 Signaling in Zebrafish NAFLD

To investigate whether the effect of Tet in improving NAFLD depends on the PI3K/AKT/STAT3 signaling pathway, we conducted experiments using the established zebrafish NAFLD model. Three groups were set up: the control group, the model group, the Tet intervention group, and the Tet plus PI3K‐specific inhibitor LY294002 treatment group (Gharbi et al. 2007). Hepatic lipid droplet accumulation was assessed by Oil Red O staining, and mRNA expression levels of PI3K, AKT, and STAT3 were measured using RT‐qPCR.

### 2.12 GEO Database Validation

#### 2.12.1 Acquisition of Gene Expression Data

Gene expression data from the GSE126848 dataset in the GEO database (https://www.ncbi.nlm.nih.gov/geo/) were used for validation. This dataset includes 15 NAFLD patients, 16 with NASH, and 14 controls. The genes with low expression in all samples were filtered, standardized, and log was taken according to the gene expression level, treated, and the coefficient of variation (CV) of gene expression was calculated, and genes with CV < 10% were filtered. R language was used to classify the gene expression data of the dataset, including 14 normal samples and 31 NAFLD patients, for subsequent analysis.

#### 2.12.2 Identification of STR Alkaloids and NAFLD‐Related Hub Genes Based on WGCNA

Weighted Gene Co‐Expression Network Analysis (WGCNA) was performed using the R “WGCNA” package to assess co‐expression modules. Modules with significant correlation to clinical features were identified, and genes with the greatest correlation were considered NAFLD‐related. Modules with a correlation coefficient > 0.75 were considered to have similar gene expression patterns. We also analyzed the expression levels of key core genes.

### 2.13 Network Analysis of NAFLD TCM Syndromes

Based on a systematic literature review and the *Expert Consensus on Traditional Chinese Medicine Diagnosis and Treatment of Nonalcoholic Fatty Liver Disease* (2023), we summarized the major TCM syndrome patterns associated with NAFLD, including “Ganyuqizhi”, “Ganshenbuzu”, “Gansenyinxu”, “Qiyinliangxu”, “Yuxuezuluo”, and “Gandanshire”. These syndrome terms were used as keywords to retrieve corresponding syndrome‐related target genes from the SymMap database. Intersection analyses were then performed between syndrome‐related targets and the predicted targets of total STR alkaloids and tetrandrine to identify potential core syndrome‐associated targets. Subsequently, overlapping targets were subjected to GO functional annotation and KEGG pathway enrichment analysis using the DAVID database. Finally, Cytoscape was employed to construct the “STR alkaloids‐Syndrome‐Target‐Pathway” and “Tetrandrine‐Syndrome‐Target‐Pathway” regulatory networks, enabling visualization of the molecular associations among active compounds, TCM syndromes, and signaling pathways.

## 3 Results

### 3.1 Active Ingredients of STR Alkaloid

A total of 19 active components of STR alkaloids were identified from the TCMSP database. After secondary screening, five key alkaloids were selected which were N‐Methylflindersine, Tetrandrine, Betaine, Magnoflorine, and Stepharine (Table 3).

**TABLE 3:** Active ingredients of STR alkaloid.

No.	Mol ID	Molecule name	OB (%)	DL	Molecular structure
1	MOL002331	N‐Methylflindersine	32.36	0.18	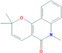
2	MOL002332	Stepharine	27.79	0.33	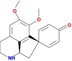
3	MOL002343	Tetrandrine	26.64	0.1	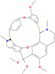
4	MOL002346	Betaine	24.8	0.55	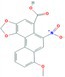
5	MOL000764	Magnoflorine	26.69	0.55	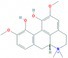

### 3.2 Drug Target Inquiry for STR Alkaloid

Using the keywords “N‐Methylflindersine”, “Tetrandrine”, “Betaine”, “Magnoflorine”, and “Stepharine”, we conducted target queries on TCMSP, DrugBank, TTD, SymMap, ChemBL, PharmaMapper, and SwissTargetPrediction. This yielded 89, 2, 3, 220, 1017, 1216, and 419 drug targets, respectively, for a total of 2966 targets. After removing duplicates, 1060 unique results were obtained. These targets were mapped to the UniProt database, yielding 592 unique drug target genes under the “ *Homo sapiens* ” condition (Table S1).

### 3.3 Target Query of NAFLD

The keyword “non‐alcoholic fatty liver disease” was used to query disease targets in the “GeneCards”, “OMIM”, “DisGeNET”, “UniProt”, “DrugBank”, and “TTD” databases, resulting in 1344, 528, 73, 4, 4, and 14 disease targets, respectively, totaling 1967. After eliminating duplicates, 1864 unique disease targets were identified (Table S2).

### 3.4 Key Target Identification via PPI Network Analysis

**FIGURE 1:**
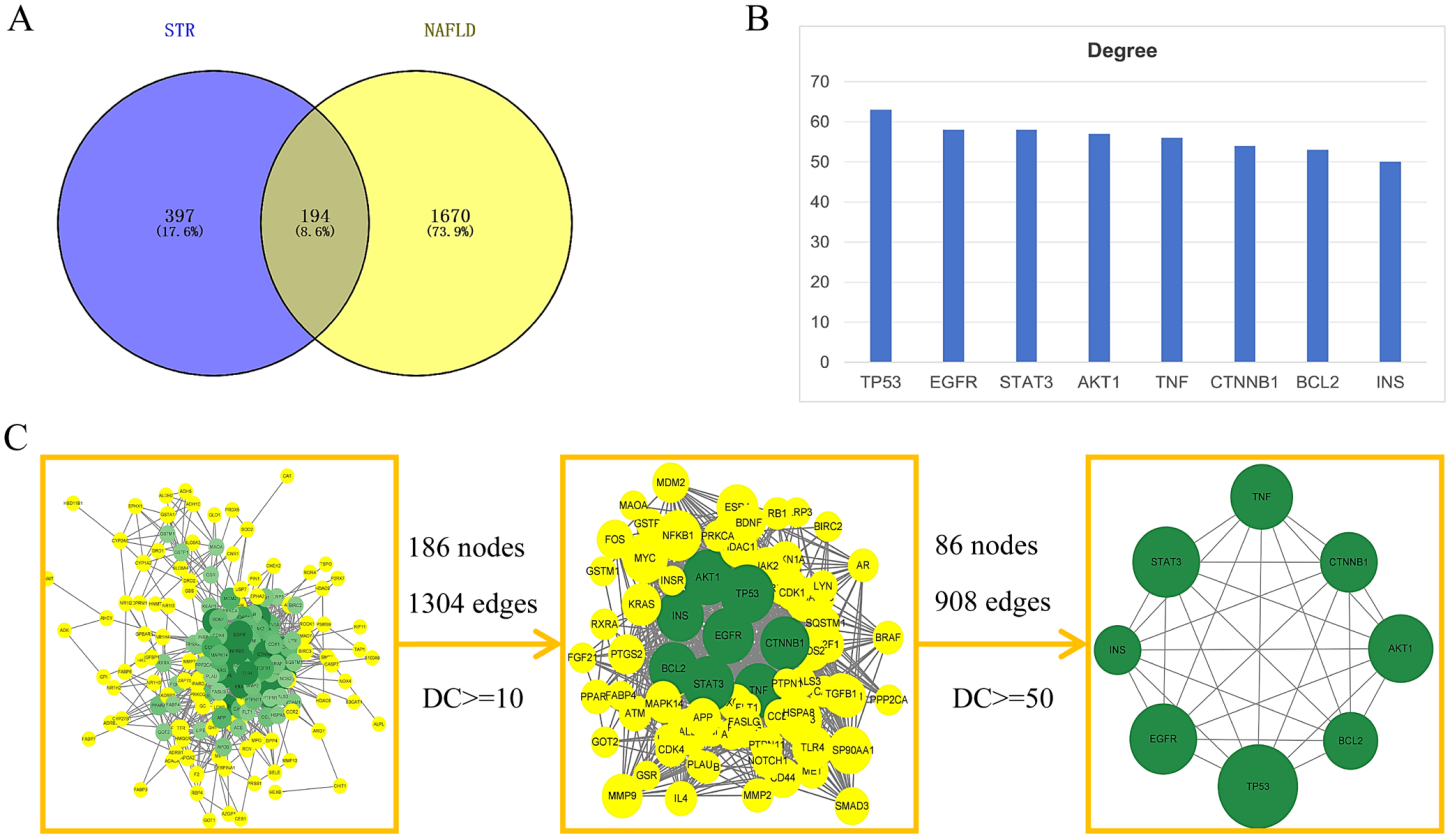
Targeting STR alkaloids in the treatment of NAFLD. (A) Venn diagram showing the intersection of targets. (B) PPI network. (C) Top eight proteins with the highest degree in the PPI network.

The 592 drug target genes and 1864 disease target genes were imported into the Venny platform to identify common genes, resulting in 194 overlapping genes (Figure 1A; Table S3). These genes were analyzed using the STRING database to construct a PPI network comprising 186 nodes and 1304 edges (Figure 1B). Topological analysis was performed using the Centiscape2.2 and CytoNCA plug‐ins, which assessed point centrality, intermediate centrality, and proximity centrality. The first screening with a degree centrality (DC) threshold of ≥ 10 yielded 86 nodes and 908 edges, while a second screening with a threshold of DC ≥ 50 identified 8 nodes and 28 edges. Key proteins identified in the PPI network as central to the treatment of NAFLD include TP53, EGFR, STAT3, AKT1, TNF, CTNNB1, BCL2, and INS, with degree values of 63, 58, 58, 57, 56, 54, 53, 53, and 50, respectively (Figure 1C).

### 3.5 GO and KEGG Pathway Enrichment Analysis

**FIGURE 2:**
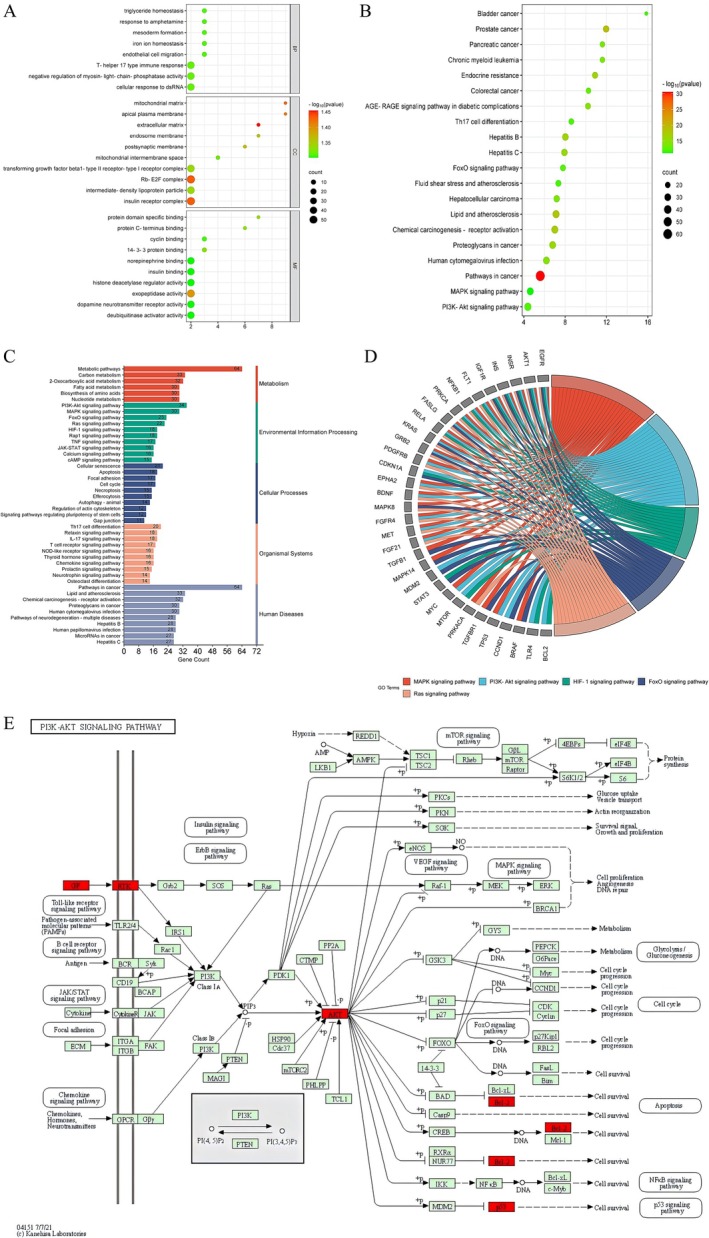
Key targets of STR alkaloids for the treatment of NAFLD. (A) GO enrichment analysis. (B) KEGG pathway enrichment analysis. (C) Classification of KEGG pathway enrichment results. (D) Relationships between the top five enriched pathways and related key genes. (E) The role of the PI3K‐AKT signaling pathway, with key proteins in red.

The intersection genes of drug targets and disease targets were uploaded to the David online platform for GO and KEGG pathway. A total of 1117 items were obtained from GO enrichment, among which 867, 89, and 152 items were BPs, CCs, and MFs, respectively. BP mainly involved fungal defense response, triglyceride homeostasis, iron ion homeostasis, mesoblastic formation, reaction to amphetamine, and negative regulation of myosin light chain phosphatase activity. CC mainly includes apical plasma membrane, mitochondrial matrix, endocytosomal membrane, and extracellular matrix, etc. MF mainly includes protein domain specific binding, protein C‐terminal binding, cyclin binding, and 14–3‐3 protein binding. Display the top 10 items with *p*‐value < 0.05 using a bubble chart (Figure 2A; Table S4). KEGG results showed that most were involved in cancer‐related enrichment pathways, MAPK signaling pathway, P13K–Akt signaling pathway, and FoxO signaling pathway (Figure 2B; Table S5).

In addition, a total of 306 pathways were obtained by the Mapper analysis tool in the KEGG database. The first 20 pathways were selected and displayed in a bubble map after crossing with the Metascape database, pathways arranged according to the number of classified and enriched genes were obtained, among them, the most important is the PI3K‐AKT signaling pathway (Figure 2C; Table S6). According to the number of enriched genes in each pathway, after crossing with the David database, in order to prove the mechanism of the treatment of NAFLD by STR alkaloids, the top five pathways with the highest number of enriched genes were displayed on the string diagram. EGFR, AKT1, INSR, INS, and IGF1R are highly correlated with phosphorylation, stress, and metabolism (Figure 2D). Key node proteins in signaling pathways were labeled in red, and the pathway containing the key node proteins is the PI3K‐AKTsignaling pathway (Figure 2E).

### 3.6 MCODE Cluster Analysis

**FIGURE 3:**
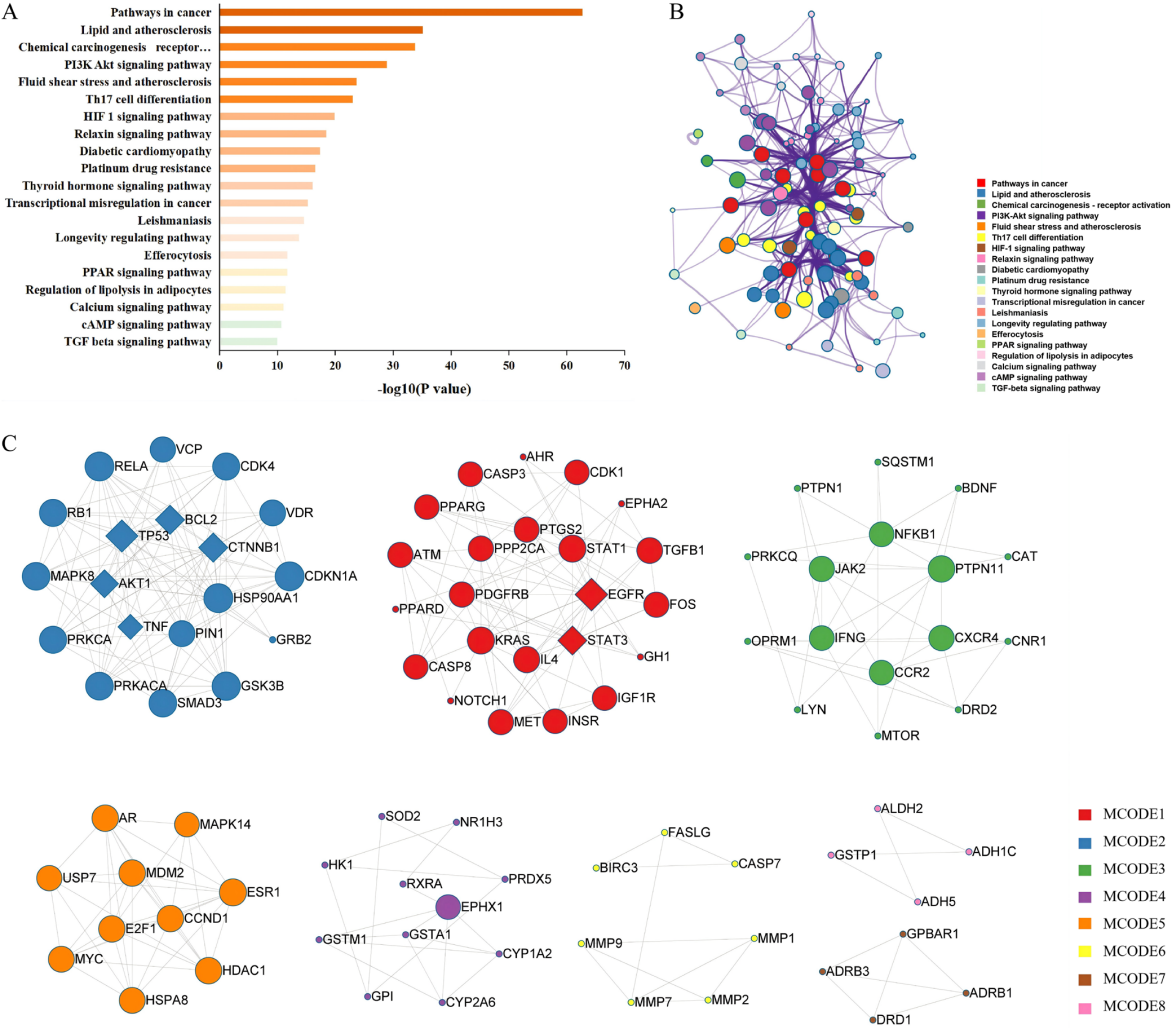
Cluster module analysis of related protein targets of STR alkaloids in NAFLD. (A) Highly enriched terms of STR alkaloids in NAFLD. (B) Subnetwork showing specific interactions. (C) Cluster analysis of STR alkaloids in NAFLD.

To explore the mechanism by which STR alkaloids regulate NAFLD, a modular network was constructed using the MCODE algorithm to identify core therapeutic targets. Topological network analysis revealed key pathways including cancer, lipid metabolism, atherosclerosis, chemical carcinogenesis, and the PI3K‐Akt signaling pathway (Figure 3A). A subset network was generated to show the relationships between targets and pathways (Figure 3B), offering insights into the potential functions of targets in different clusters. MCODE cluster analysis of the 194 intersecting genes identified 8 common target modules for STR alkaloids in the treatment of NAFLD (Figure 3C). The key genes in these modules are represented as diamonds. Notably, MCODE1, including genes such as BCL2, TP53, AKT1, TNF, and CTNNB1, was associated with pathways in cancer, human cytomegalovirus infection, and hepatitis B. MCODE2, which included EGFR and STAT3, was enriched in pathways related to cancer, MAPK signaling, and human papillomavirus infection (Table 4; Table S7).

**TABLE 4:** Pathway enrichment results for MCODE1 and MCODE2.

MCODE	Go	Description	Log10 (P)
MCODE1	hsa05200	Pathways in cancer	−22.9
MCODE1	hsa05167	Human cytomegalovirus infection	−21.0
MCODE1	hsa05163	Hepatitis B	−20.3
MCODE2	hsa05200	Pathways in cancer	−22.9
MCODE2	hsa05167	MAPK signaling pathway	−14.1
MCODE2	hsa05163	Human papillomavirus infection	−13.7

### 3.7 Network Construction of “Disease‐Active Ingredients‐Targets‐Pathway” Interactions

**FIGURE 4:**
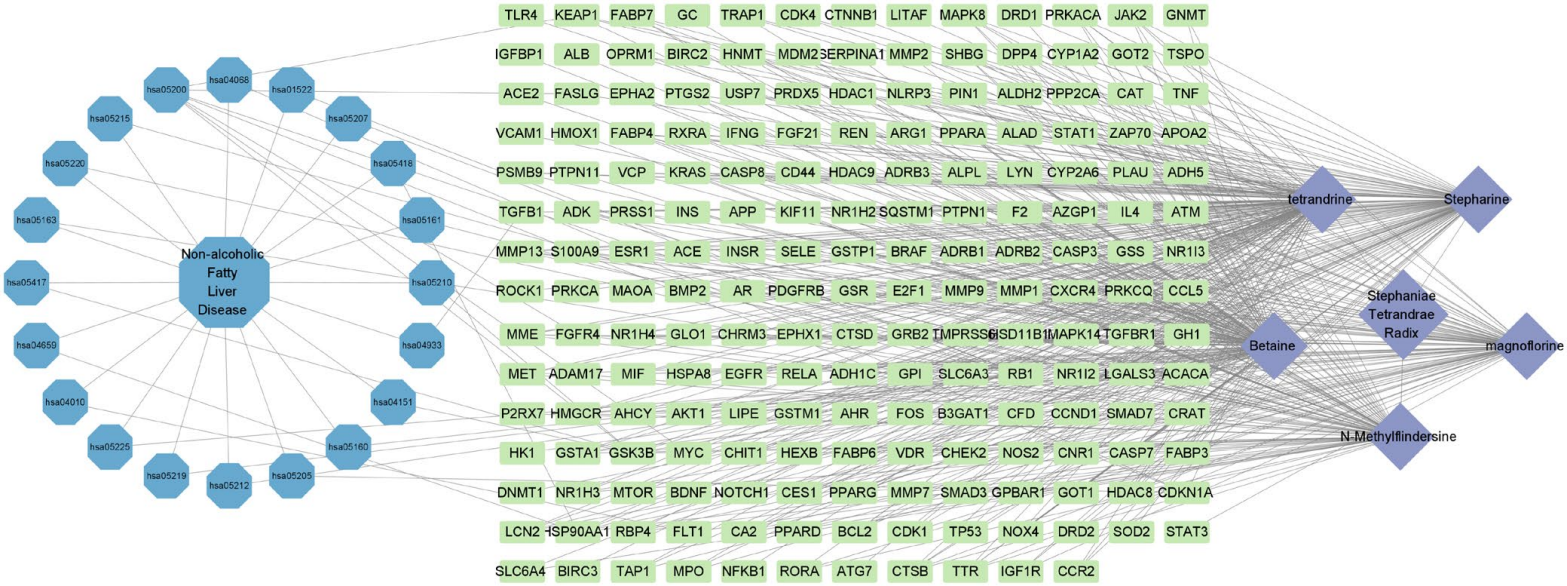
“Disease‐Active ingredients‐Targets‐Pathway” diagram of STR alkaloids in NAFLD.

To visualize the relationships between active ingredients, core targets, and enriched pathways, a “disease‐active ingredients‐targets‐pathway” network diagram was created using Cytoscape 3.9.1 software (Figure 4). The network contained 227 nodes and 420 edges, with the blue hexagons representing disease and pathway names, purple diamonds indicating STR alkaloids, and green rectangles representing common targets. Key pathways included cancer, lipid metabolism, atherosclerosis, chemical carcinogenesis, hepatitis B, and other related pathways. The purple rectangles indicate common drug‐target interactions, with straight lines showing their relationships.

### 3.8 ADME Evaluation and Toxicity Analysis of STR Alkaloids

Pharmacokinetic analysis of STR alkaloids was conducted using the SwissADME database. Except for Tet, which has a high molecular weight, and Betaine, which is highly hydrophilic, the remaining compounds complied with Lipinski's rule of five. Although Tet has a high molecular weight, its high gastrointestinal absorption suggests it may rely on transporter‐mediated transmembrane pathways. A systematic evaluation of the toxicological parameters (such as hERG inhibition, hepatotoxicity and carcinogenicity) revealed Tet posed a higher risk for hERG inhibition, while N‐Methylflindersine and Betaine presented an elevated risk of hepatotoxicity. N‐Methylflindersine also carried dual risks for carcinogenicity and AMES toxicity. Stepharinewas highly carcinogenic but not genotoxic, with both compounds showing relatively low ocular toxicity. Consequently, Magnoflorine and Betaine exhibited low combined toxicity, while Tet and Stepharine may require modifications in dosage form or indications to mitigate systemic toxicity (Tables 5 and 6).

**TABLE 5:** AMDE evaluation of core components.

Parameters	N‐Methylflindersine	Stepharine	Tetrandrine	Betaine	Magnoflorine
MW ≤ 500	241.29	297.35	622.75	117.15	342.41
HBA< 10	2	4	8	2	4
HBD ≤ 5	0	1	0	0	2
MLog *p* ≤ 4.15	2.63	1.81	3.73	−3.67	−1.71
TPSA (Å2) < 140	31.23	47.56	61.86	40.13	58.92
Rotatable bonds < 10	0	2	4	2	2
GI absorption	High	High	High	Low	High
BBB permeability	YES	YES	NO	NO	YES

**TABLE 6:** Toxicity analysis of core components.

Parameters	N‐Methylflindersine	Stepharine	Tetrandrine	Betaine	Magnoflorine
hERG	0.033	0.633	0.987	0.008	0.109
Human hepatotoxicity	0.835	0.508	0.538	0.784	0.022
Carcinogens	0.895	0.895	0.031	0.052	0.021
AMES Toxicity	0.831	0.024	0.084	0.003	0.162
Eye corrosion	0.003	0.003	0.003	0.064	0.003

### 3.9 Molecular Docking Verification

**FIGURE 5:**
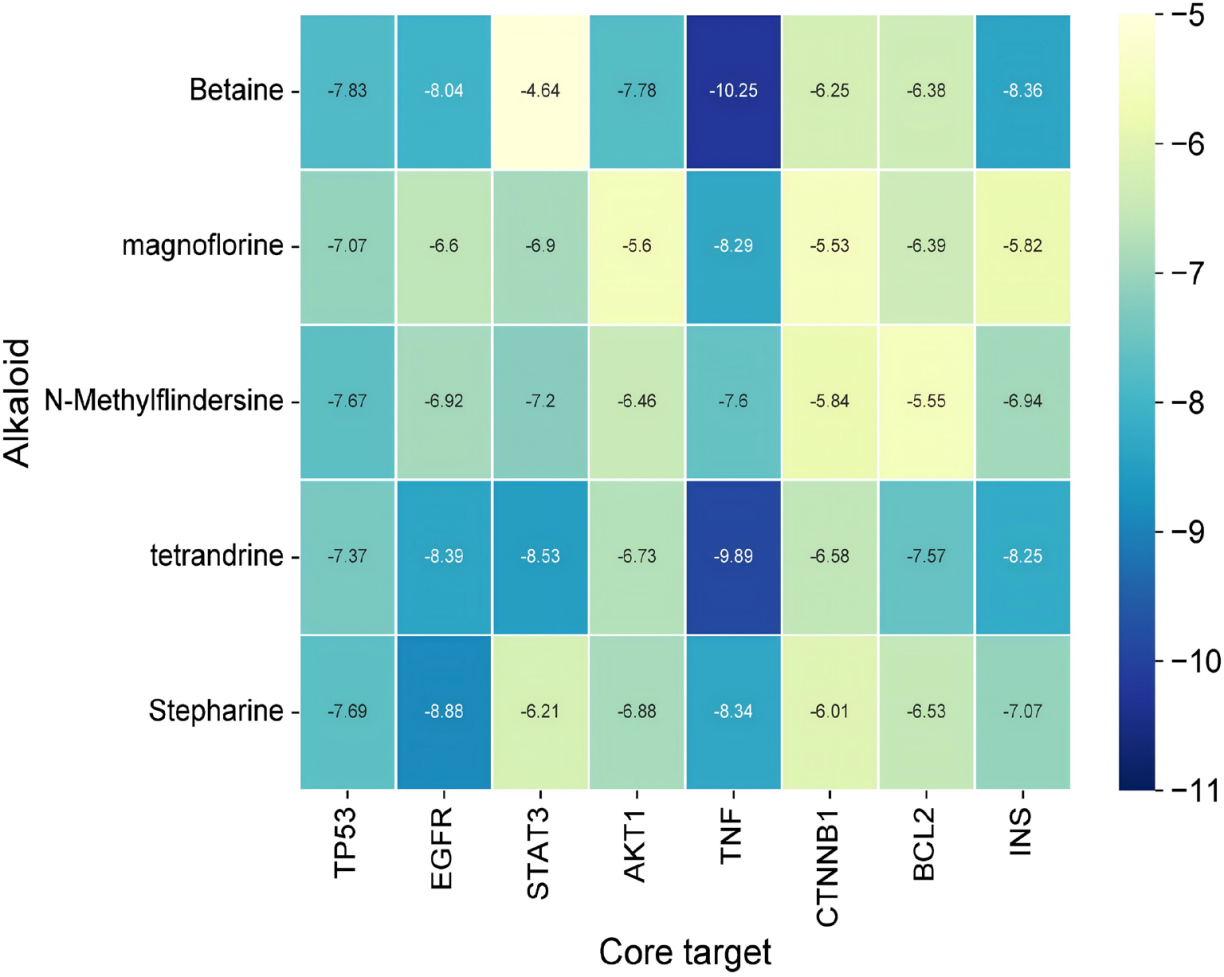
Docking results of core active ingredients and key node proteins.

Molecular docking was performed to predict the therapeutic potential of the alkaloids by analyzing the binding energies between their active components and key target proteins. The selected components, N‐Methylflindersine, Tet, Betaine, Magnoflorine, and Stepharine, were docked with the core proteins TP53, STAT3, EGFR, AKT1, TNF, and CTNNB1. The results indicated strong interactions between Betaine and TNF, and between Tet and all target proteins, with the lowest binding energy observed for TNF and STAT3. Binding energy values above 8.5 kcal/mol were considered significant for molecular interactions (Figure 5).

**FIGURE 6:**
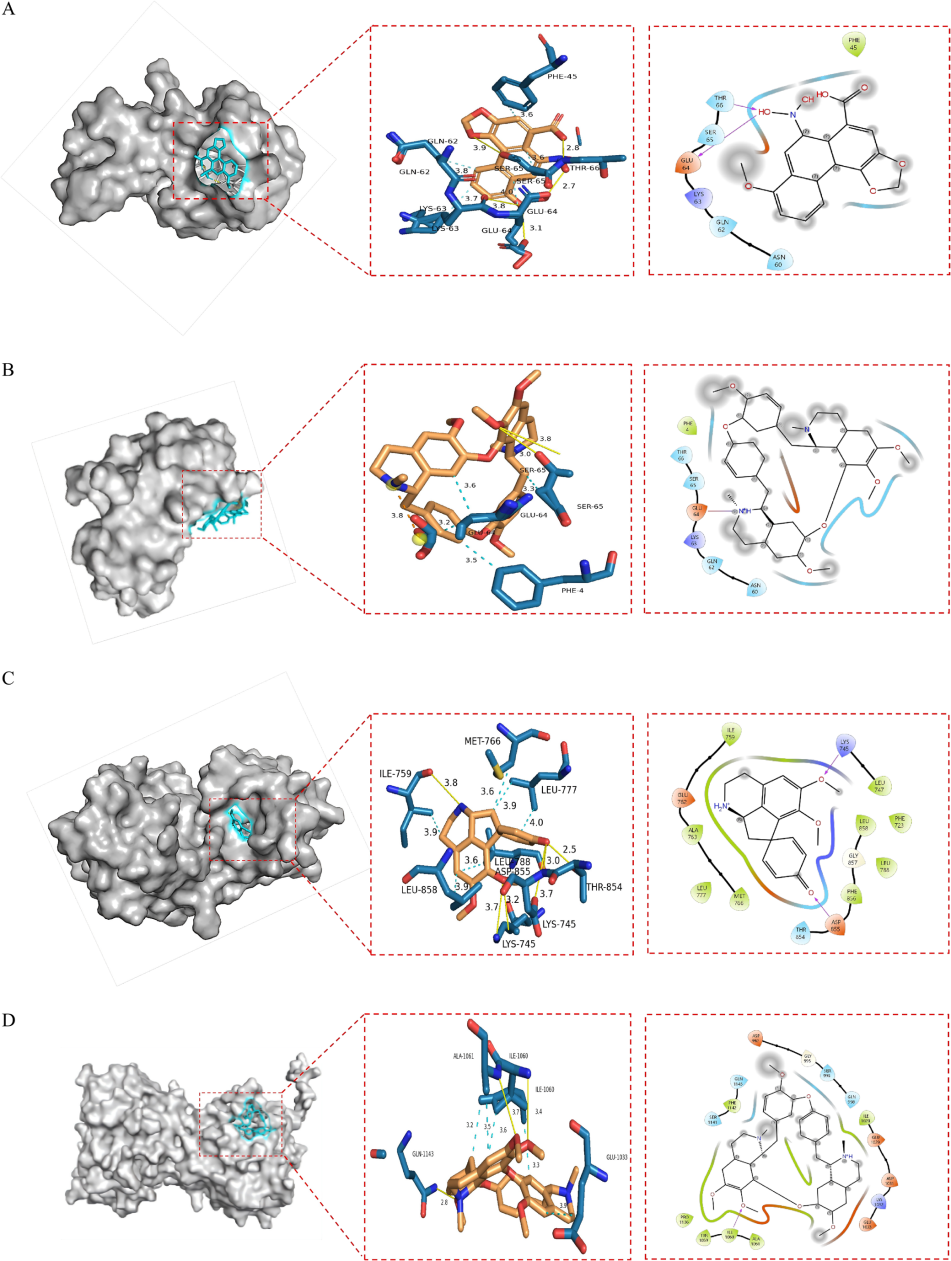
Molecular docking results of alkaloids with the highest binding energy molecules of each of the eight core proteins. (A) TNF‐Betaine. (B) TNF‐Tet. (C) EGFR‐Stepharine. (D) STAT3‐Tet.

For TNF‐Betaine, as shown in Figure 6A, betaine formed hydrophobic interactions with PH‐45 (3.6 Å), GLN‐62 (3.8 Å), LYS‐63 (3.7 Å), GLU‐64 (4.0 Å), and SER‐65 (3.6 Å). It also formed hydrogen bonds with LYS‐63 (3.8 Å), SER‐65 (3.9 Å), and THR‐66 (2.8 Å), as well as two hydrogen bonds with GLU‐64 (3.1 and 2.7 Å). For TNF‐Tet in Figure 6B, Tet formed hydrophobic interactions with PH‐4 (3.5 Å), SER‐65 (3.3 Å), and GLU‐64 (3.6 and 3.2 Å). It formed two hydrogen bonds with SER‐65 (3.0 and 3.8 Å) and a salt bridge with GLU‐64. For EGFR and stephine, as shown in Figure 6C, hydrophobic interactions were observed with ILE‐759 (3.9 Å), MET‐766 (3.6 Å), LEU‐788 (3.6 Å), and LEU‐858 (3.9 Å). Two additional hydrophobic interactions were formed with LEU‐777 (4.0 and 3.9 Å). Hydrogen bonds were formed with ILE‐759 (3.8 Å), THR‐854 (2.5 Å), and two hydrogen bonds with LYS‐745 (3.2 and 3.7 Å) and ASP‐855 (3.0 and 3.7 Å). For STAT3 and Tet, as shown in Figure 6D, hydrophobic interactions were formed with GLU‐1033 (3.9 Å) and ALA‐1061 (3.5 Å), as well as with ILE‐1060 (3.6, 3.2, and 3.3 Å). Hydrogen bonds were formed with ILE‐1060 (3.4 Å), ALA‐1061 (3.7 Å), and GLN‐1143 (2.8 Å).

### 3.10 Molecular Dynamics Simulations

MD simulations provide valuable insights into the dynamic stability of receptor‐ligand complexes under physiological conditions. Considering binding affinity, intra‐complex interactions, and the structural diversity of the compounds, the molecular docking results discussed earlier were selected for MD simulations to evaluate their binding stability.

**FIGURE 7:**
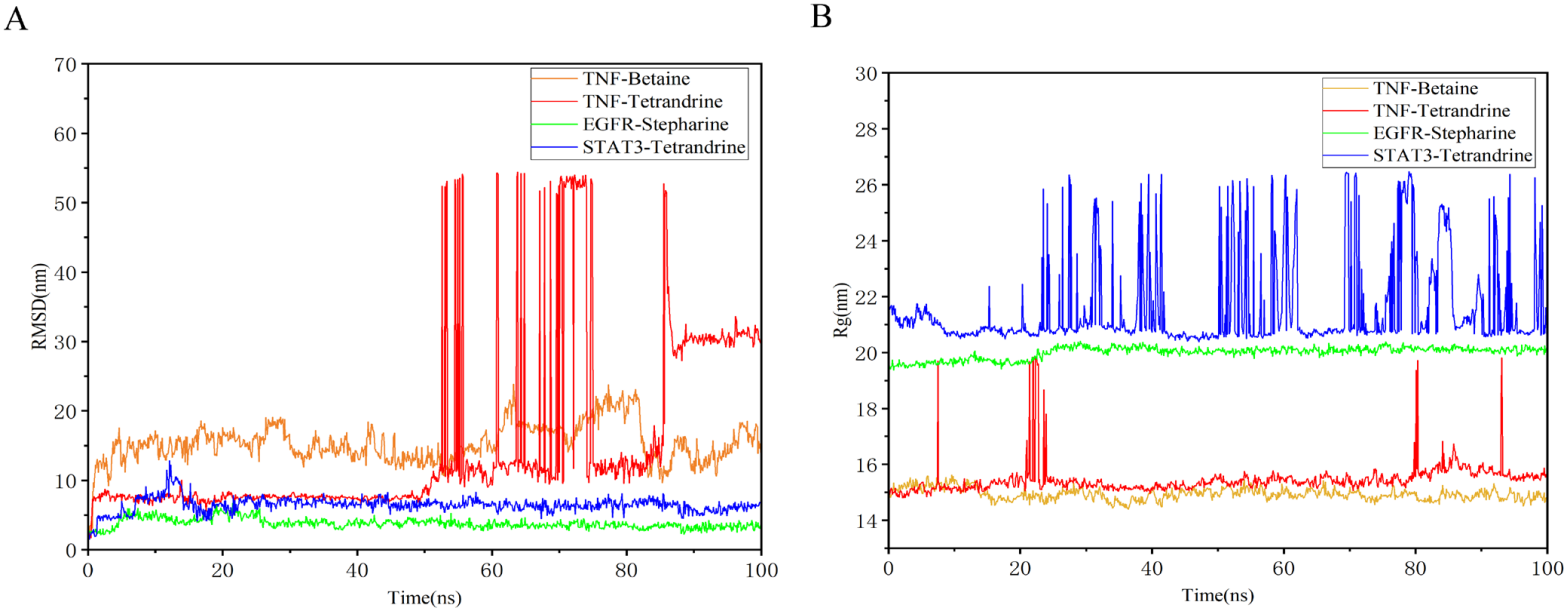
The fluctuation plot of the target protein‐ligand complexes (A) RMSD (B) Rg.

The RMSD measures the difference between the real‐time conformation and the initial conformation during the simulation process and serves as a key index for determining whether the system has reached equilibrium. The stability of each system was assessed by monitoring the RMSD of the simulated trajectories over 100 ns. A stable system typically exhibits RMSD fluctuations below 0.2 nm. In Figure 7A, the RMSD curves for the three protein–ligand complexes indicated stable trajectories throughout the simulations. These complexes showed consistently low fluctuation ranges, suggesting stability over time. For example, the RMSD curves for Stepharine‐EGFR and Tet‐STAT3 complexes displayed some fluctuations at the start. After 5 ns, Stepharine‐EGFR stabilized at 3–5 Å, while Tet‐STAT3 stabilized at 5–7 Å after 15 ns. This indicates that both interactions become stable after 15 ns. Notably, the conformations of EGFR and STAT3 did not change significantly upon Tet binding, suggesting the stability of this complex.

**FIGURE 8:**
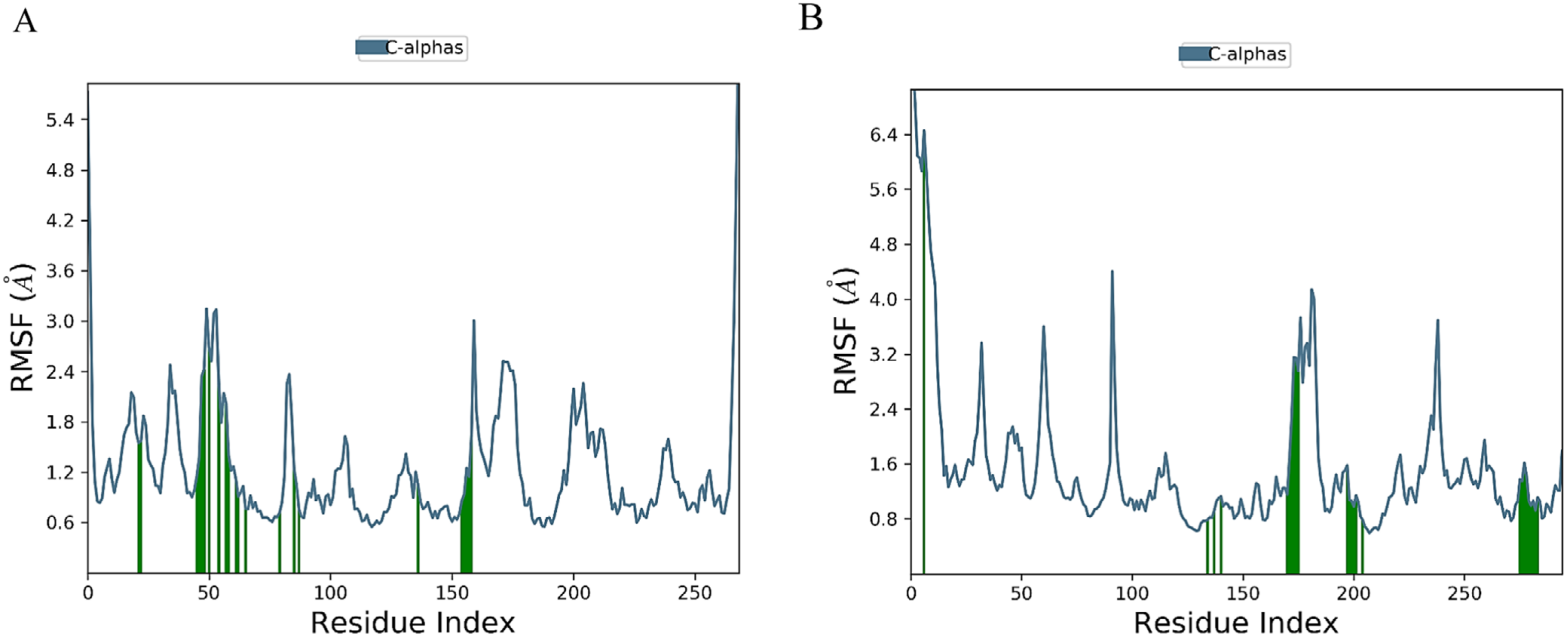
The fluctuation plot of the target protein‐ligand complexes RMSF. (A) EGFR‐Stepharine. (B) STAT3‐Tetrandrine.

Based on the RMSD and pharmacokinetic results, both stepharine and Tet showed good stability, warranting further analysis of their RMSF, hydrogen bonds and radius of gyration (Rg). The Rg is an indicator of the compactness and level of constraint within a molecular system, reflecting the degree of protein folding. A higher Rg value suggests lower stability, while a lower Rg value indicates a more compact and stable system. As shown in Figure 7, the TNF‐Betaine and EGFR‐Stepharine complexes exhibited stable fluctuations within the 0–100 ns time range. The Rg value for the TNF‐Betaine complex fluctuated around 15 nm, and when compared with the STAT3‐Tet, EGFR‐Stepharine and TNF‐Tet complexes, the TNF‐Betaine complex demonstrated the greatest stability (Figure 7B). RMSF quantifies the stability of amino acid residues within proteins, providing an indicator of protein rigidity and dynamic stability. In the RMSF analysis, the Stepharine‐EGFR complex exhibited greater fluctuations in residues 47–53 and 172–178, while the Tet‐STAT3 complex showed greater fluctuations in residues 27–34, 59–66, 90–94, 173–186, and 232–240. These regions of higher fluctuation suggest increased flexibility within the complexes. The Stepharine‐EGFR and Tet‐STAT3 complexes exhibited reduced residue fluctuations, indicating increased rigidity in those regions. This stability suggested that both compounds effectively enhance protein stability by reducing the flexibility of amino acids within their target proteins (Figure 8).

**FIGURE 9:**
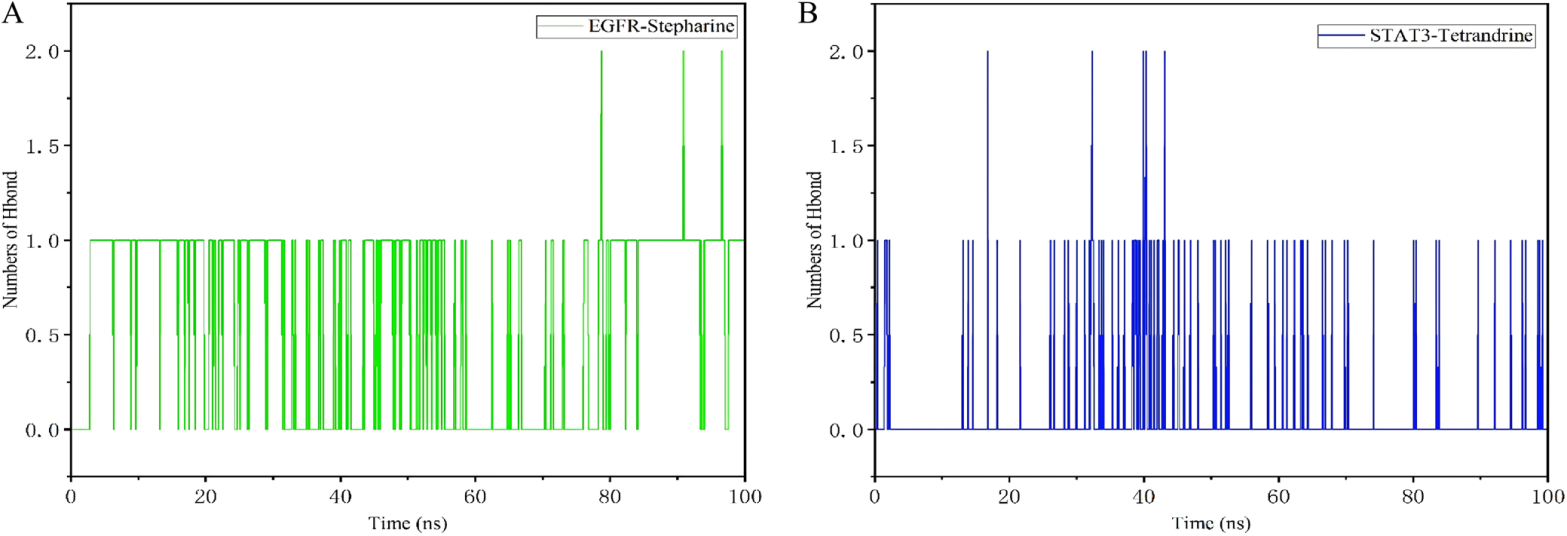
Hydrogen bond number of complex. (A) EGFR‐Stepharine. (B) STAT3‐Tetrandrine.

The complexes and target proteins displayed various interactions, including hydrogen bonding, hydrophobic interactions, and hydrophilic interactions. Figure 9 illustrated the fluctuation of hydrogen bonds (H bonds) over time during molecular dynamics simulations. Hydrogen bonding was a crucial non‐covalent interaction in protein‐ligand binding, playing a key role in stabilizing complex structures. The variation in hydrogen bond numbers reflected the dynamic movement of ligands within the receptor‐binding site, potentially influencing drug binding affinity and selectivity. Figure 9A depicted the EGFR complex with Stepharine, where the number of hydrogen bonds fluctuates between 0 and 2 over a 100‐ns simulation period. While the number of hydrogen bonds was predominantly 1, it occasionally increased to 2. Despite these fluctuations, hydrogen bonding remained relatively stable overall, suggesting that Stepharine forms stable hydrogen bond interactions with EGFR, which helped maintain the structure of the complex. The fluctuating hydrogen bond numbers may represent the ligand's dynamic movement within the binding site. Figure 9B showed the STAT3‐Tet complex, where hydrogen bond fluctuations mirror those observed in the EGFR‐Stepharine complex. This suggested that Tet also interacted with STAT3 via hydrogen bonds, contributing to the stability of the complex. In summary, the hydrogen bond patterns for both complexes were similar, indicating comparable stability driven by hydrogen bonding interactions.

### 3.11 Results of MR Analysis

**FIGURE 10:**
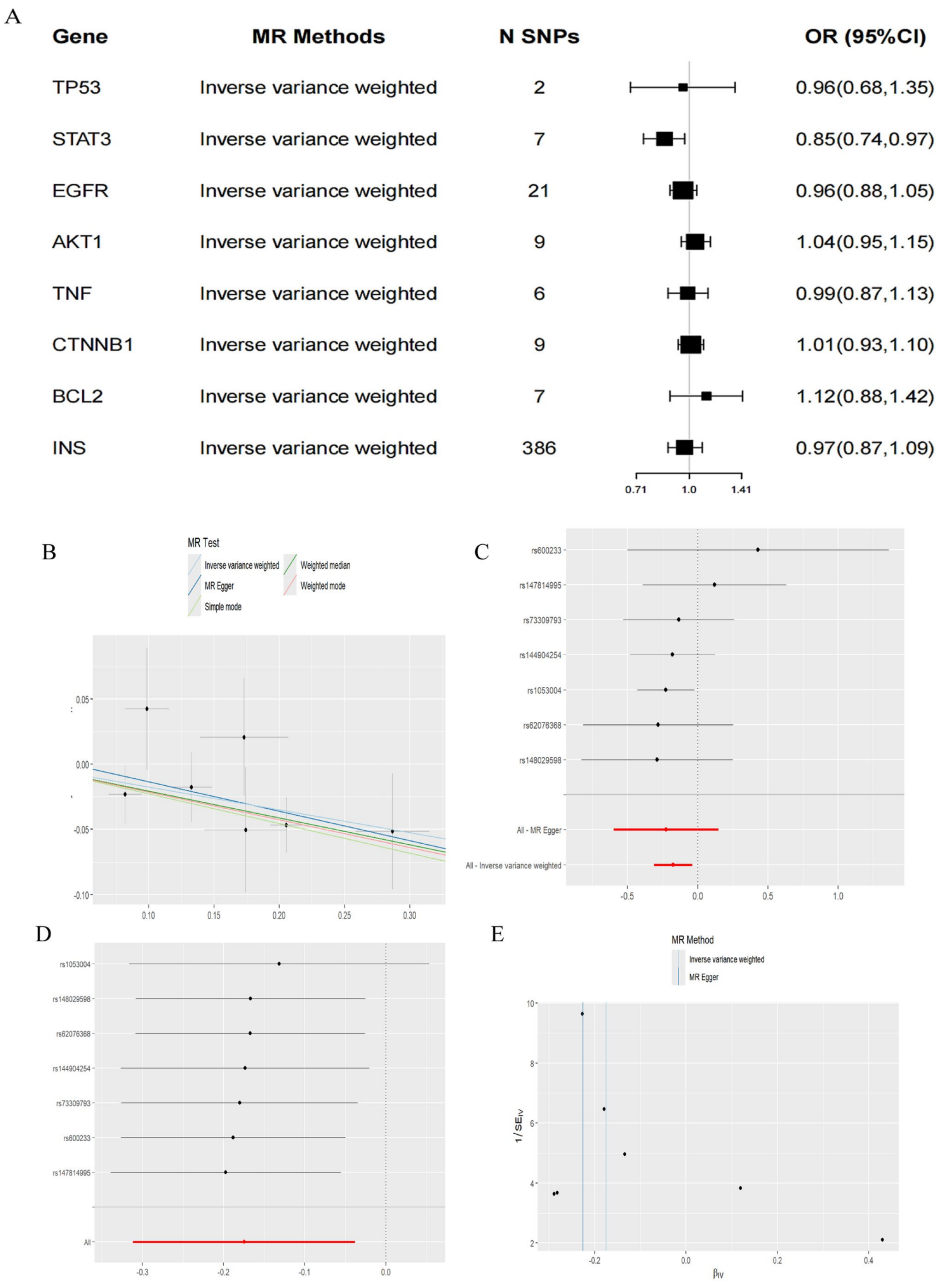
Results of MR analysis of key genes on NAFLD. (A) Forest plot of causal relationships between the eight candidate genes and NAFLD using the IVW method. (B) Causal effect scatterplot of MR analysis of the relationship between STAT3 and NAFLD. (C) Causal effect forest plot of each SNP in STAT3 on NAFLD risk. (D) Leave‐one‐out plot for STAT3 and NAFLD risk after removing one SNP. (E) STAT3 and NAFLD relationship SNP‐biased funnel plot.

MR analyses were conducted to explore the causal relationship between specific genes and NAFLD. Eight candidate genes, TP53, STAT3, EGFR, AKT1, TNF, CTNNB1, BCL2, and INS were assessed for potential causal associations with NAFLD. The SNPs used in the analysis are listed in Table S8, and all selected SNPs were identified as strong IVs. Figure 10A showed the causal effect of each gene on NAFLD.

The results indicated that, except for STAT3, no significant causal relationships were observed between the other seven genes and NAFLD. The IVW analysis revealed that higher levels of STAT3 were associated with an increased risk of NAFLD after Bonferroni correction, with an OR of 0.85 (95% CI: 0.74–0.97) and a *p*‐value of 0.02 (Figure 10B and Figure 10C). A leave‐one‐out analysis (Figure 10D) confirmed the robustness of these results, showing consistency even after excluding individual SNPs. Sensitivity analyses for the STAT3 gene further validated the robustness of the findings. The funnel plot for STAT3 (Figure 10E) appeared roughly symmetrical, indicating no significant publication bias.

Additionally, Cochran's Q test showed no significant heterogeneity (*p* = 0.66), supporting the consistency and robustness of the results. The MR‐Egger regression intercept was 0.01 (*p* = 0.79), suggesting no horizontal pleiotropy. MR‐PRESSO analysis (RSSobs = 4.53, *p* = 0.827) also indicated no significant pleiotropic effect. Based on these sensitivity analyses, we conclude that the causal relationship between STAT3 and NAFLD is statistically significant and not influenced by heterogeneity or horizontal pleiotropy. Detailed statistical results are provided in Tables S9 and S10.

### 3.12 Intersection Genes, Key Target PPI Construction, and Core Protein Selection of Tet in NAFLD

**FIGURE 11:**
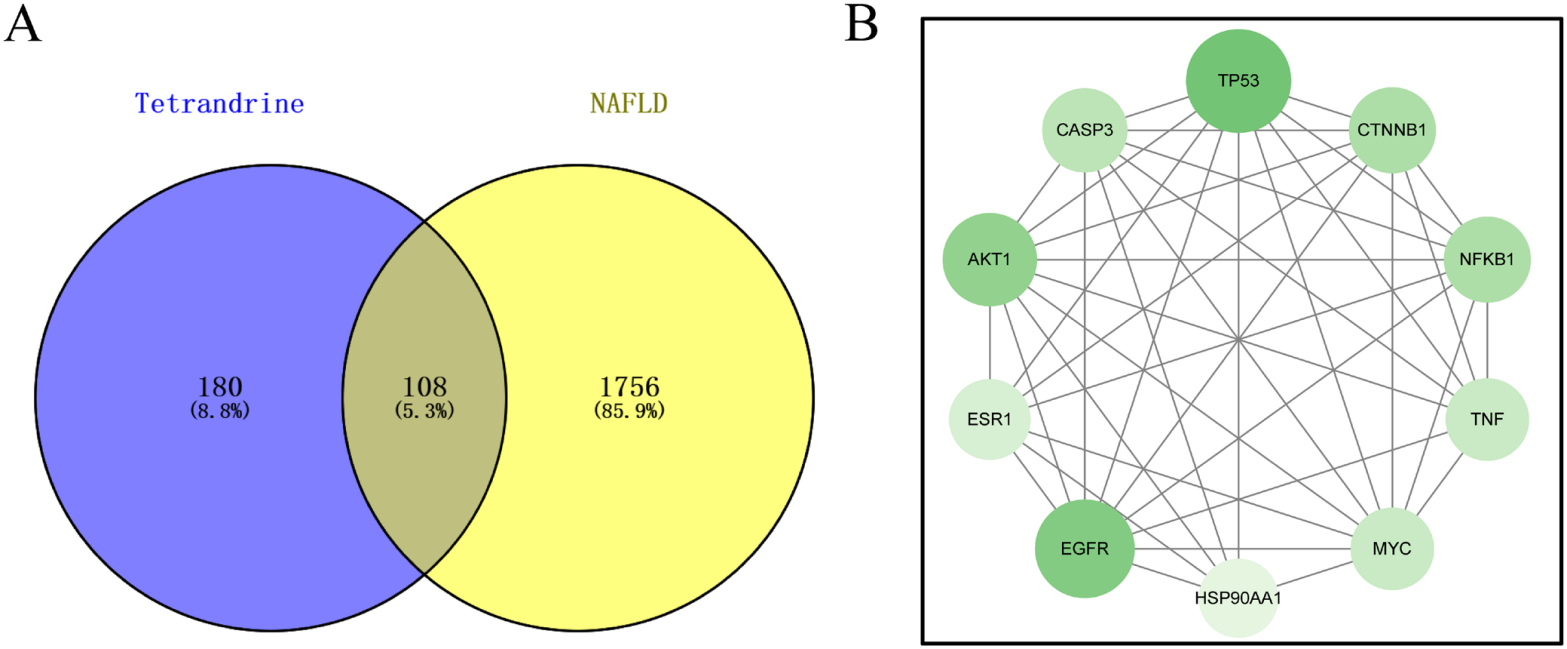
Targeting of Tet in the treatment of NAFLD. (A) Intersection target Venny diagram (B) PPI network.

A total of 288 Tet target genes and 1864 NAFLD target genes were imported into the Venny online mapping platform, resulting in 108 common genes (Figure 11A; Table S11). These common genes were imported into the String platform to build the PPI network using Cytoscape 3.9.1 software. Centiscape2.2 and CytoNCA plug‐in are used for network topological analysis to identify 10 nodes and 40 edges with DC ≥ 50 thresholds and identify key nodes with high centrality. PPI network analysis shows that TP53, EGFR, AKT1, TNF, CTNNB1, NFKB1, CASP3, MYC, ESR1, and HSP90AA1 are key proteins involved in NAFLD treatment (Figure 11B; Table S12).

### 3.13 GO Function and KEGG Pathway Enrichment Analysis

**FIGURE 12:**
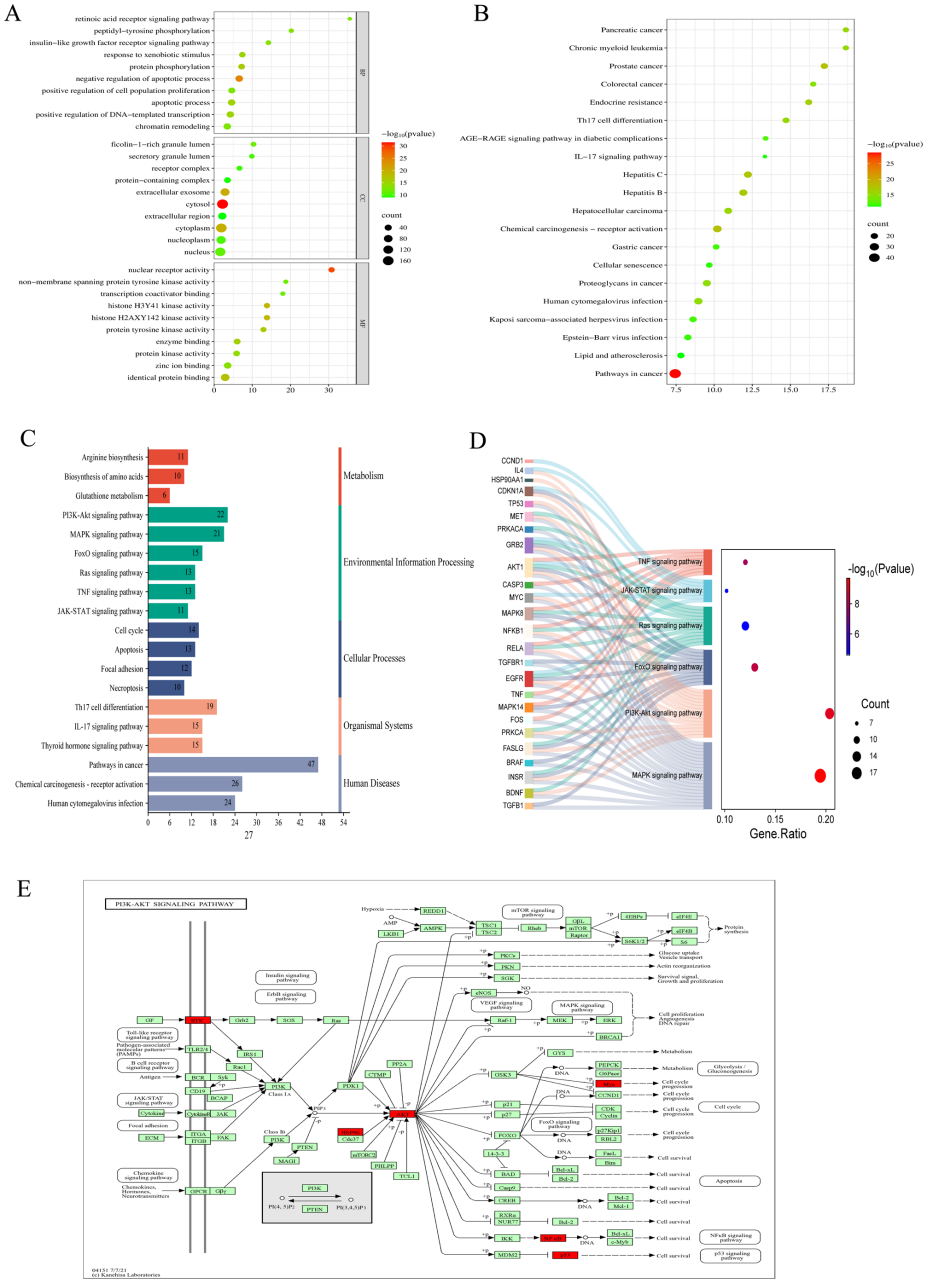
Key targets of STR alkaloids for the treatment of NAFLD. (A) GO enrichment analysis. (B) KEGG pathway enrichment analysis. (C) Classification of KEGG pathway enrichment results. (D) Sangita diagram of key pathways and genes. (E) PI3K‐AKT signaling pathway.

The intersection 108 genes of Tet targets and NAFLD disease targets were uploaded to the DAVID platform for GO and KEGG pathway enrichment analysis. A total of 1117 GO terms were obtained, including 534 BPs, 58 CCs, and 169 MFs. BP mainly involved protein phosphorylation, negative regulation of apoptotic process, apoptotic process and positive regulation of DNA‐templated transcription. CC mainly includes cytosol, extracellular region, and cytoplasm, etc. MF mainly includes nuclear receptor activity, identical protein binding, and protein tyrosine kinase activity. The top 10 items with *p*‐value < 0.05 are selected to be displayed in bubble chart (Figure 12A; Table S13). KEGG results showed that most were involved in cancer‐related enrichment pathways (Figure 12B; Table S14).

**FIGURE 13:**
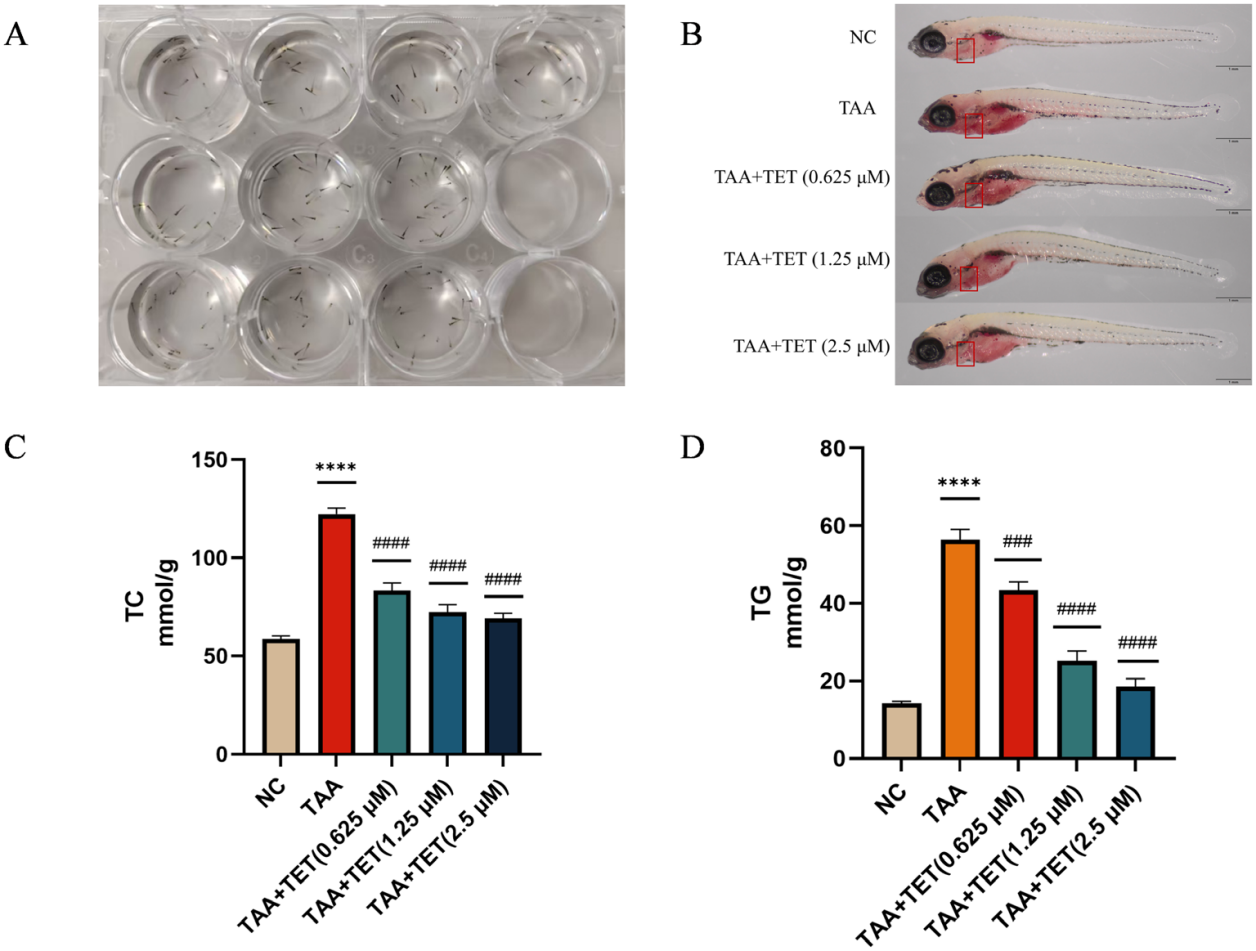
Liver steatosis and lipid deposition of juvenile zebrafish reduced by Tet. (A) Juvenile zebrafish modeling. (B) Oil red O staining results. (C) TC levels in each group. (D) TG levels in each group. Values are expressed as the mean ± SD in each group. *Compared with control group, ****p < 0.0001; ^#^compared with TAA group, ^###^ *p* < 0.001, and ^####^ *p* < 0.0001.

In addition, a total of 277 paths were obtained in the KEGG database by the Mapper analysis tool; the most important pathway is the PI3K‐Akt signaling pathway, MAPK signaling pathway (Figure 12C). After crossing with the David database, select the first 20 paths to display in the bubble chart. After hybridization with the Metascape database, the pathways arranged according to the number of classified and enriched genes were obtained. EGFR, AKT1 are highly correlated with the PI3K‐Akt signaling pathway, MAPK signaling pathway (Figure 12D). The key node proteins in the signaling pathway are marked in red, and the pathway containing the key node proteins is the PI3K‐AKT signaling pathway (Figures 12E and 13).

### 3.14 Establishment of the NAFLD Model in Zebrafish Larvae and Analysis of Oil Red O Staining, TG and TC Content

Oil Red O staining showed pronounced lipid accumulation (dark red staining) in the model group, which was markedly reduced after Tet treatment, indicating severe disruption of lipid metabolism and significant lipid accumulation in the liver. Following Tet intervention, lipid accumulation in the liver was notably reduced, with medium and high concentrations of Tet effectively improving lipid deposition (Figure 13A,B). The levels of TC and TG in the model group were significantly higher compared to the control group (****p < 0.001). Treatment with Tet significantly reduced both TC and TG levels in the zebrafish (###*p* < 0.001), as shown in Figure 13C,D. These results suggest that Tet can alleviate lipid metabolism disorders and liver lipid accumulation in NAFLD zebrafish.

### 3.15 Effects of Tet on Gene Expression Related to the PI3K/AKT/STAT3 Signaling Pathway in Juvenile NAFLD Zebrafish

**FIGURE 14:**
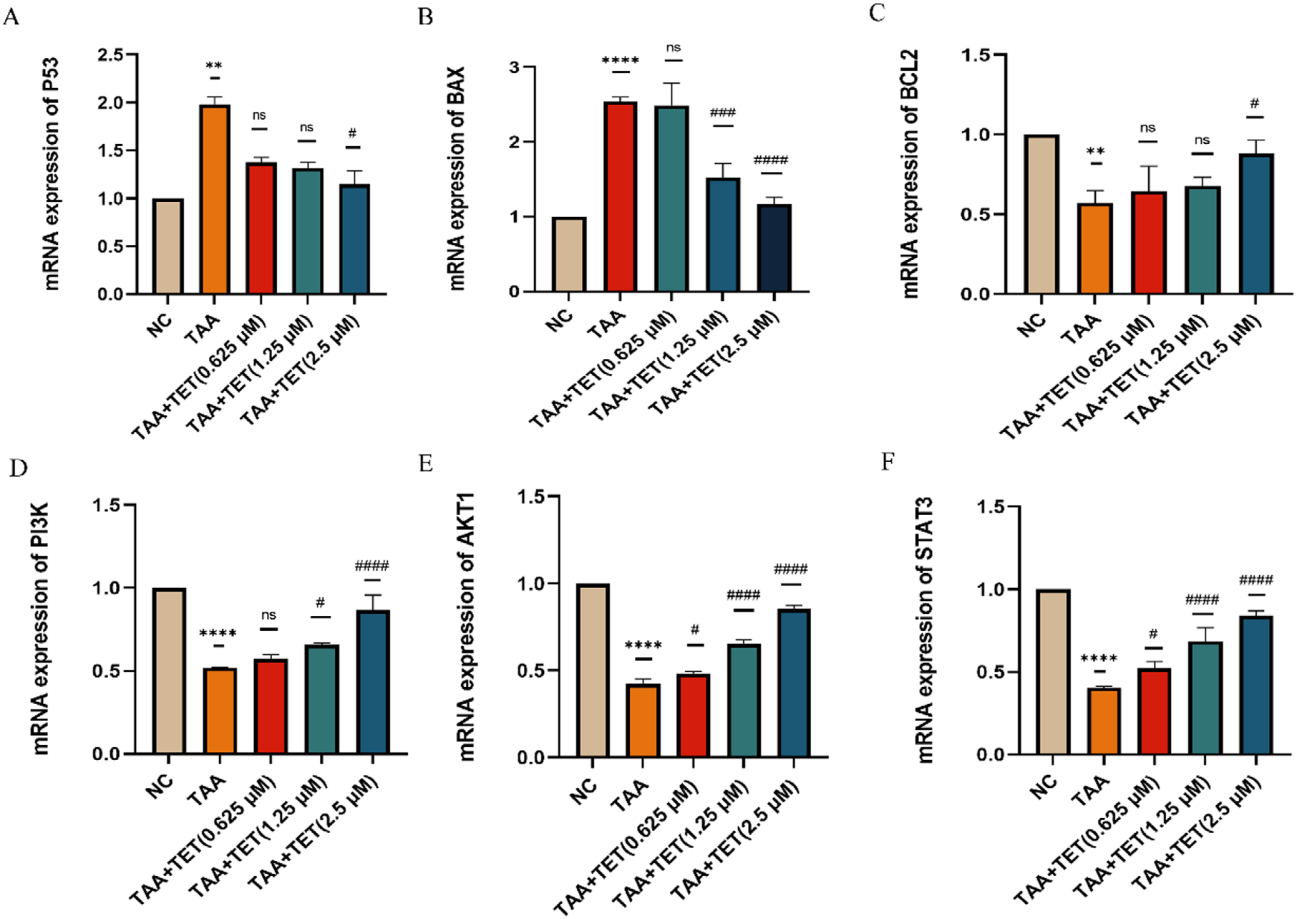
The expression of related genes was verified by PCR. (A) P53 mRNA expression level. (B) *BAX* mRNA expression level. (C) *BCL2* mRNA expression level. (D) *PI3K* mRNA expression level. (E) *AKT1* mRNA expression level. (F) *STAT3* mRNA expression level. *Compared with control group, **p < 0.01 and ****p < 0.0001; ^#^compared with TAA group, ^##^ *p* < 0.05, ^###^ *p* < 0.001 and ^####^ *p* < 0.0001.

Based on network pharmacology predictions, we examined the expression levels of genes involved in apoptosis and the PI3K/AKT signaling pathway, including P53, BCL2, Bax, PI3K, AKT, and STAT3. The tumor suppressor protein P53 plays a key role in cell apoptosis. qPCR results showed that the expression levels of P53 and Bax were significantly higher in the model group compared to the control group (**p < 0.01), while BCL2 expression was significantly lower (**p < 0.01). After Tet treatment, the expression of P53 and Bax were significantly reduced (#*p *< 0.05), whereas BCL2 expression was significantly increased (#*p* < 0.05) (Figure 14A–C). These findings suggest that Tet may induce apoptosis by regulating p53, BCL2 and Bax protein expression.

The expression levels of PI3K and AKT were significantly lower in the model group compared to the control group (****p < 0.0001). Furthermore, treatment with Tet significantly increased the expression of PI3K, AKT, and STAT3 compared to the model group (####*p* < 0.0001) (Figure 14D–F). These results indicate that the PI3K/AKT signaling pathway is inhibited in the model group, and that Tet can activate this pathway. Thus, Tet appears to inhibit apoptosis through the PI3K/AKT/STAT3 signaling pathway.

### 3.16 Effect of LY294002 on Tet‐Mediated PI3K/AKT/STAT3 Signaling in the Zebrafish NAFLD Model

**FIGURE 15:**
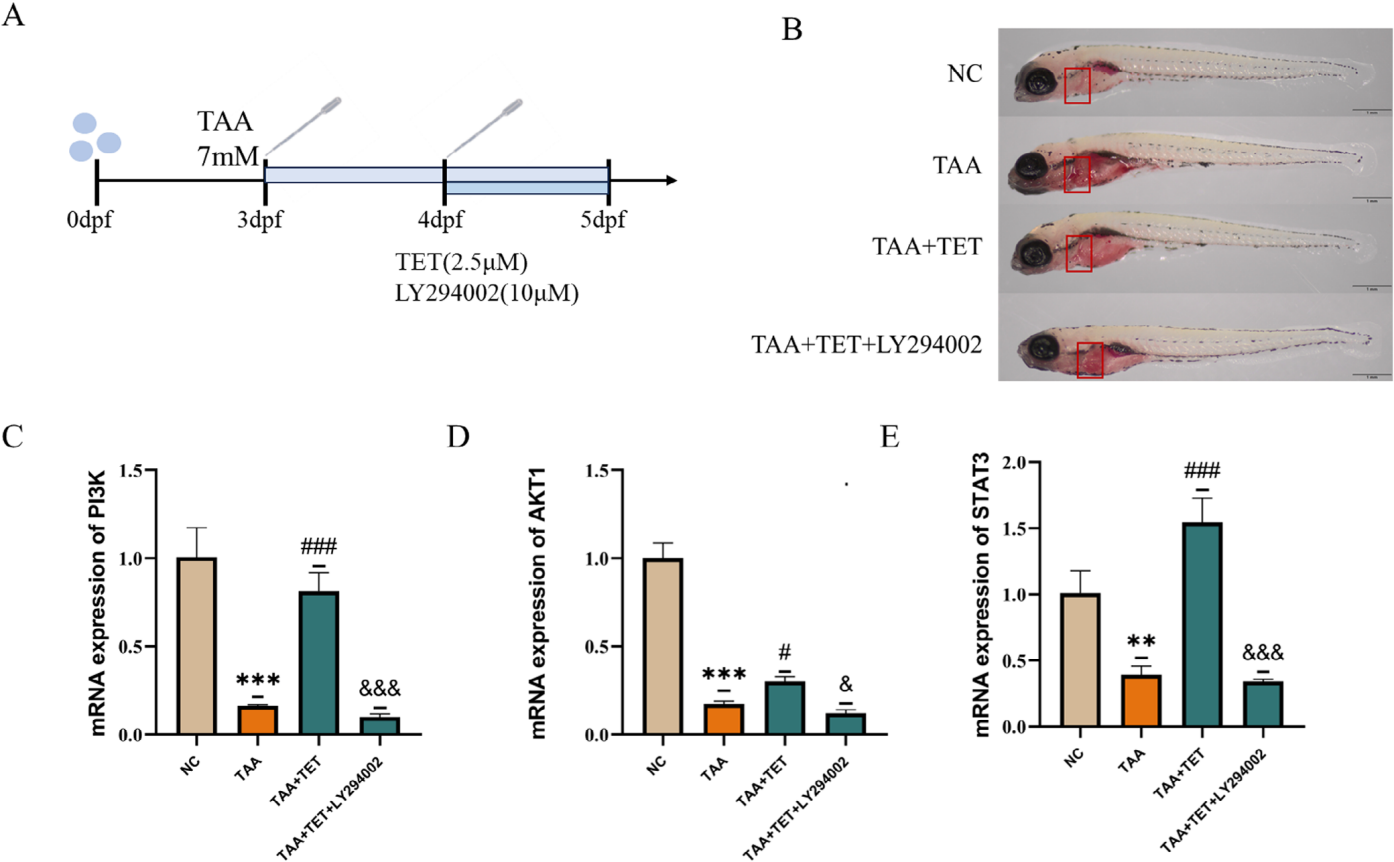
Effect of LY294002 on Tet‐mediated PI3K/AKT/STAT3 signaling in NAFLD model. (A) Schematic of the experimental design. (B) Oil red O staining results. (C–E) mRNA expression levels of PI3K, AKT, and STAT3. *Indicates comparison with the NC group: *p < 0.05, **p < 0.01, ***p < 0.001. ^#^Indicates comparison with the TAA group: ^#^ *p* < 0.05, ^###^ *p* < 0.01, ^####^ *p* < 0.001. ^&^Indicates comparison with the TAA+TET group: ^&^ *p* < 0.05, ^&&^ *p* < 0.01, ^&&&^ *p* < 0.001.

Compared with the model group, zebrafish in the Tet intervention group showed significantly reduced hepatic lipid droplet accumulation, accompanied by a marked increase in mRNA expression levels of PI3K, AKT, and STAT3 (#*p* < 0.05). When LY294002 was co‐administered, the Tet‐induced improvement in hepatic lipid accumulation was markedly attenuated, and the mRNA expression levels of the pathway‐related genes were significantly lower than in the Tet‐only group (&*p* < 0.05). These results indicate that Tet's ameliorative effect on NAFLD lipid deposition may be associated with activation of the PI3K/AKT/STAT3 signaling pathway (Figure 15).

### 3.17 Using WGCNA to Identify Genes Associated With NAFLD

**FIGURE 16:**
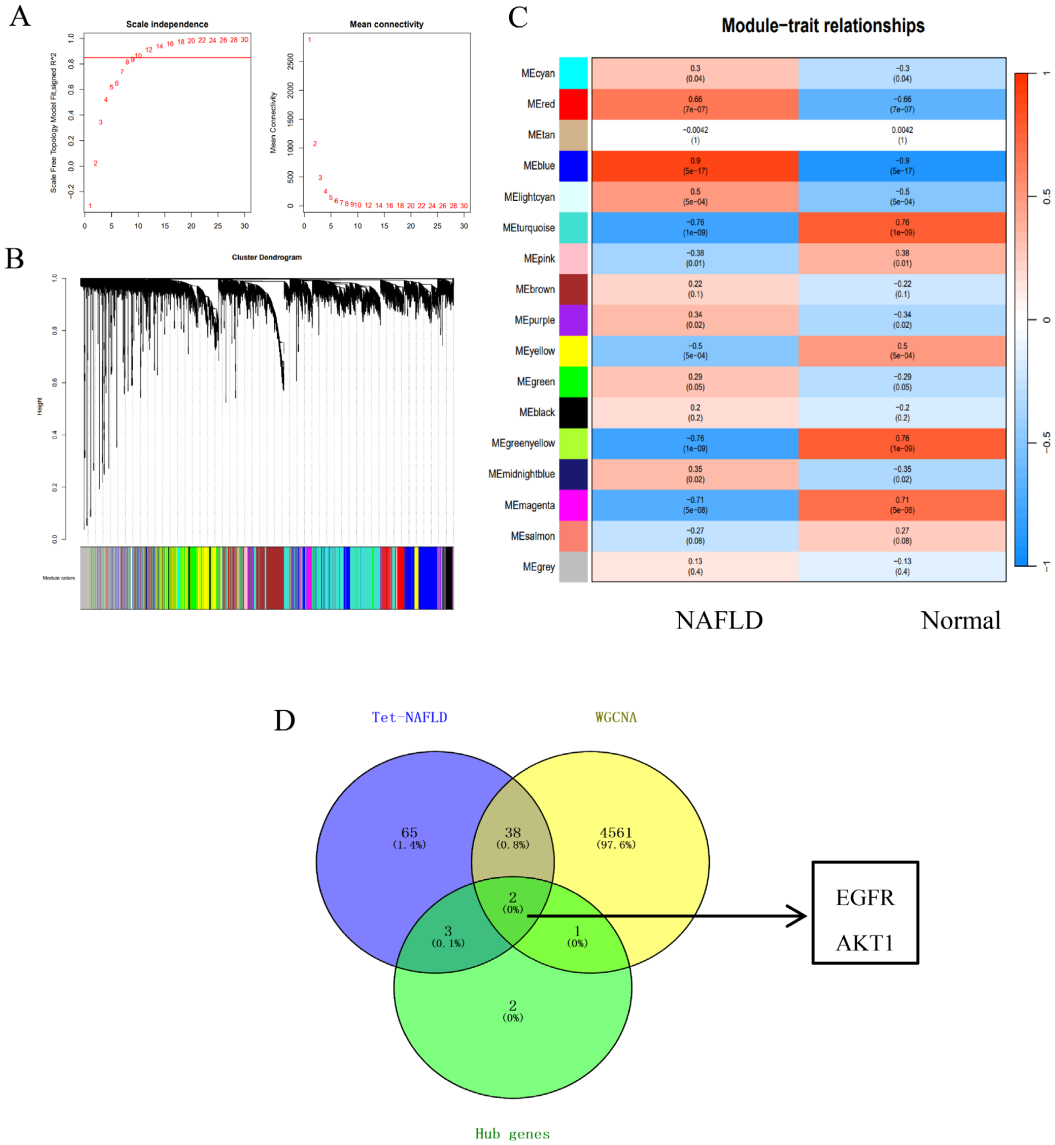
Using GEO database and WGCNA to verify the core genes of STR alkaloids in NAFLD. (A) The scale‐free topological degree and module connectivity of different soft thresholds. (B) The tree diagram of gene module division when the soft threshold is determined to be 10. (C) The correlation between modules and NAFLD. (D) Intersection genes between the most correlated genes in WGCNA, Tet‐NAFLD, and hub genes.

WGCNA was performed on 11,561 genes to further identify key genes associated with NAFLD. Cluster analysis revealed the correlation between all samples, and a weighted gene co‐expression network was constructed using the expression matrix of DEGs across all samples. The soft threshold was set to 10 (*R* ^2^=0.86), which exceeds the threshold of 0.8. The mean connectivity was 17.52, and a scaling free network was constructed (Figure 16A). By combining modules with high correlation, 17 co‐expression modules were identified, with a minimum of 30 genes per module (Figure 16B). Pearson correlation analysis between module genes and NAFLD identified MEblue as most strongly correlated with NAFLD (cor = 0.9, *p* = 5 × 10^−17^). MEturquoise, MEgreenyellow and significant correlation between NAFLD and highest (cor = 0.76, *p* = 1e^−9^), was selected as the target module (Figure 16C). Using gene importance and module correlation as screening criteria (importance > 0.5, correlation > 0.75), 81 target genes were obtained. According to the intersection genes, key core genes and 81 target genes of WGCNA, it was found that EGFR, and AKT1 were the core genes for final verification (Figure 16D).

### 3.18 Network Analysis of “NAFLD‐Syndrome‐Tetrandrine‐Target‐Pathway”

Using the SymMap database, we identified syndrome‐related targets corresponding to the six NAFLD‐associated TCM syndromes. Intersection analysis between these targets and predicted STR alkaloid targets yielded 11 overlapping genes (TREM1, LTF, PNMT, AKR1C3, F7, GSTM2, GAD2, KCNA5, IDO1, KCNH2, and TSPO). KEGG enrichment analysis indicated that these genes were primarily involved in metabolic pathways, taurine and hypotaurine metabolism, steroid hormone biosynthesis, drug metabolism, and glutathione metabolism. The “STR alkaloids‐Syndrome‐Target‐Pathway” network was subsequently constructed (Figure S1).

**FIGURE 17:**
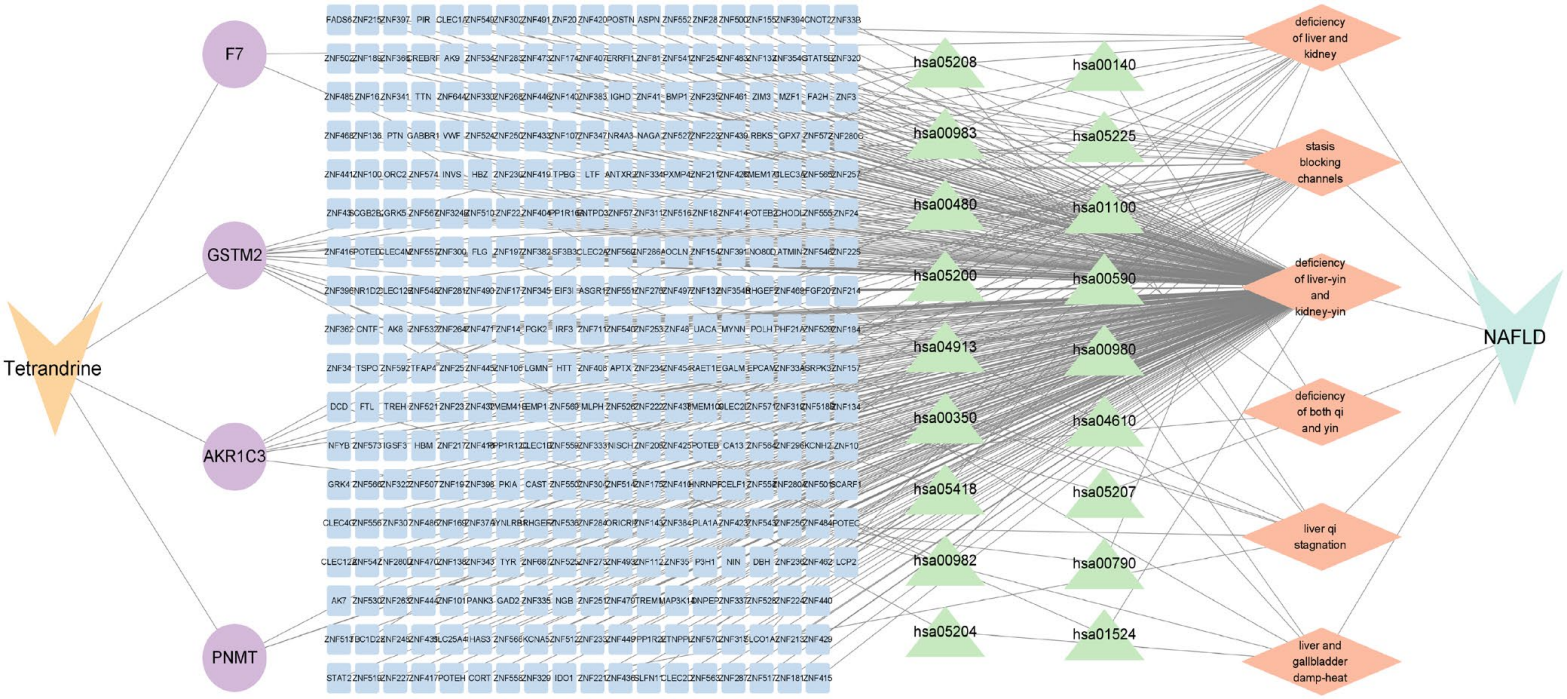
“Tet‐syndrome‐target‐pathway” regulatory network.

Further intersection analysis between syndrome‐related targets and predicted tetrandrine targets identified four overlapping genes (PNMT, AKR1C3, F7, and GSTM2). KEGG enrichment analysis revealed significant enrichment in metabolic pathways, tyrosine metabolism, folate biosynthesis, and steroid hormone biosynthesis. The corresponding “Tetrandrine‐Syndrome‐Target‐Pathway” regulatory network was established (Figure 17), illustrating the potential molecular mechanisms of tetrandrine within the NAFLD TCM syndrome framework.

## 4 Discussion

The pathophysiology of NAFLD involves complex interactions between multiple factors, which collectively contribute to its development. These factors include lipid metabolism disorders, lipid toxicity, endoplasmic reticulum stress, insulin resistance, intestinal microbiome dysbiosis, and oxidative stress, all of which culminate in excessive hepatic fat accumulation and the progression of NAFLD (Musso et al. 2018; Neumann et al. 2018; Parola and Pinzani 2024; Watt et al. 2019). Given its multifactorial nature, developing multi‐target therapeutic strategies is essential. TCM, with its ability to target multiple pathways simultaneously, holds promise as a potential treatment for NAFLD.

Tet, a bisbenzylisoquinoline alkaloid, has been clinically used for the treatment of silicosis, hypertension, and arrhythmia. Accumulating pharmacological evidence demonstrates that it exerts diverse biological activities, including anti‐inflammatory, antioxidant, anti‐fibrotic, immunomodulatory, and anti‐apoptotic effects (Chan et al. 2021; Mo et al. 2022; Qian et al. 2024), which align closely with the central pathological features of NAFLD, persistent low‐grade inflammation, oxidative stress, dysregulated lipid metabolism, and progressive fibrosis. The multi‐target regulatory properties of Tet, particularly its ability to modulate inflammatory signaling, alleviate oxidative stress, and restore metabolic balance, provide a strong rationale for its therapeutic potential in NAFLD. Additionally, computational analyses further supported its selection as a candidate drug. Tet exhibited the highest number of overlapping targets with NAFLD‐related genes, favorable binding affinities to key targets such as STAT3, AKT1, and TP53, and predicted pharmacokinetic and safety advantages compared with other STR alkaloids. Integrating network pharmacology, in silico simulations, and prior pharmacological evidence, Tet was thus identified as the core active component for mechanistic investigation in the zebrafish NAFLD model.

To further elucidate the molecular mechanisms of Tet in NAFLD, we integrated network pharmacology predictions with experimental validation, revealing that the PI3K/AKT signaling pathway is a key mediator of Tet's effects. This pathway regulates lipid metabolism homeostasis, cell migration, and apoptosis (Su et al. 2023). Our findings show that in zebrafish models, Tet treatment significantly activated the PI3K/AKT pathway, suggesting its involvement in regulating lipid metabolism. The PI3K/AKT pathway is closely associated with hepatic lipid deposition (Yilmaz and Cizmecioglu 2025). It enhances glucose uptake of glucose transporter 4 (GLUT4) membrane translocation, lipid biogenesis, and reduces lipid degradation (Ramachandran and Saravanan 2015). Studies have shown that NUP85 can reduce lipid deposition and inflammation in the liver by inhibiting the PI3K/AKT pathway; in a methionine‐choline deficient mouse model, activation of the PI3K/AKT pathway leads to increased liver triglyceride content and promotes lipid drop formation by upregulating fatty acid synthetase (FASN) and stearoyl‐CoA desaturase 1 (SCD1) (Wu et al. 2024). Chronic low‐grade inflammation plays a crucial role in the progression of NAFLD, and the PI3K/AKT pathway is known to modulate immune cell recruitment and activation, thus regulating the inflammatory response (Hawkins and Stephens 2015; Satapathy and Sanyal 2015). Additionally, the PI3K/AKT‐NRF2 pathway has been shown to alleviate oxidative stress and suppress NF‐κB‐mediated inflammation during NAFLD progression (J. Li et al. 2021).

Our study further demonstrated that Tet regulates hepatocyte apoptosis. Tet treatment reduced the expression of the anti‐apoptotic protein BCL2 while increasing the levels of pro‐apoptotic factors Bax and p53. This aligns with the known regulatory role of the PI3K/AKT pathway in apoptosis, where it inhibits BCL2 and activates Bax and p53 pathways (M. Chen et al. 2022; Xie et al. 2022). In NAFLD, excessive fat accumulation in hepatocytes, in the form of triglycerides, is a hallmark of the disease, often accompanied by hepatocyte ballooning degeneration and varying degrees of fibrosis (Arab et al. 2020). Zhang et al. found that Nourishing Yin and moistening dryness formula can activate the PI3K/AKT signaling pathway and inhibit colon cell apoptosis, thereby reducing the symptoms of Yin deficiency constipation mice (Hanyu Zhang et al. 2023). Liu et al. found that Glycyrrhiza flavone inhibits apoptosis through the PI3K/AKT signaling pathway and improves gastric ulcer in ethanol‐induced rats (Guo et al. 2024). AKT was an upstream regulator of STAT3 when treated with an AKT inhibitor (Tang et al. 2022). We also found that Tet may influence STAT3 signaling, a key regulator of liver lipid homeostasis and fibrosis (Zhao et al. 2021). Our findings suggested that Tet's therapeutic effects on NAFLD may be mediated through the PI3K/AKT/STAT3 signaling pathway, as evidenced by reduced levels of PI3K, AKT1, and STAT3 in the treatment group compared to controls.

The pharmacological effects of STR alkaloids, particularly tetrandrine, demonstrate a multi‐target and multi‐pathway regulatory pattern. At the molecular level, this regulatory profile aligns closely with the key pathological components emphasized in NAFLD‐related TCM syndromes, including “dampness”, “phlegm” and “blood stasis” (Cheng et al. 2026; Ding et al. 2024; Z. Liu et al. 2025; Zheng et al. 2025). By integrating network pharmacology results with syndrome‐specific target analysis derived from the SymMap database, the present study establishes a molecular‐level “syndrome‐target‐pathway” association framework. This integrative approach provides mechanistic insights into how tetrandrine may modulate biological processes corresponding to TCM syndrome characteristics, thereby enhancing the theoretical coherence and translational relevance of tetrandrine in the context of NAFLD management.

Despite promising results, this study has several limitations. Some findings relied on public databases without clinical validation, and the MR analysis was mainly based on European populations, limiting generalizability. Moreover, while the zebrafish NAFLD model offered advantages in gene homology, conserved metabolic pathways, and high‐throughput screening, its liver lacks the classical lobular architecture of mammals. Future studies will validate protein expression and functional outcomes in mammalian (e.g., mouse) models and in vitro cell systems to establish a multi‐level mechanistic evidence chain. Safety optimization will also be addressed through structural modifications to reduce hERG channel affinity and formulation strategies, such as nano‐delivery systems, to improve pharmacokinetics and reduce cardiac exposure. Finally, to systematically elucidate Tet's multi‐pathway regulation, high‐fat diet–induced mouse models will be used to examine phosphorylation changes in PI3K/AKT, MAPK, and FoxO pathways, combined with specific pathway inhibitors, to clarify pathway‐specific contributions and potential crosstalk in Tet‐mediated hepatoprotection.

## 5 Conclusion

By integrating network pharmacology, molecular simulations, bioinformatics, Mendelian randomization, and experimental validation, we explore the therapeutic effects of tetrandrine alkaloids in NAFLD and uncover the underlying molecular mechanisms. Our results indicate that tetrandrine can effectively mitigate liver dysfunction, reduce liver steatosis, lipid metabolism disorder, and promote apoptosis by modulating the PI3K/AKT/STAT3 signaling pathway. This study provides a solid theoretical foundation for further clinical research into the therapeutic potential of tetrandrine for NAFLD.

## Author Contributions

**Xiaoyu Zhu:** software, data curation. **Peng Sun:** conceptualization, methodology, writing – original draft, resources, software, funding acquisition. **Xinyu Zhang:** data curation, software. **Wei Gong:** conceptualization, writing – review and editing, writing – original draft, methodology, software, data curation, supervision. **Wenhao Han:** software, data curation. **Xi Wang:** software, data curation. **Jing Ma:** data curation, software. **Hong Lin:** conceptualization, writing – review and editing, funding acquisition. **Kai Ma:** software, data curation. **Jun Wu:** methodology, data curation. **Xiaoli Wei:** software, data curation. **Tangnuer Tuersunniyazi:** methodology.

## Funding

This work was supported by Scientific research project of Ningxia Medical University (XT2020024). Ningxia Scientific Research Projects of Higher Education Institutions (NYG2022041). Ningxia Natural Science Foundation general project (2025AAC030674).

## Conflicts of Interest

The authors declare no conflicts of interest.

## Supporting information


**Figure S1:** “STR alkaloids‐syndromes‐targets‐pathways” regulatory network.

**Table S1:** Drug target inquiry for STR alkaloid.

**Table S2:** Target query of NAFLD.

**Table S3:** Intersection genes of NAFLD and STR alkaloid.

**Table S4:** GO enrichment analysis.

**Table S5:** KEGG Pathway enrichment analysis.

**Table S6:** KEGG Pathway form database.

**Table S7:** Pathway enrichment results for MCODEs.

**Table S8:** SNPs information for the 8 feature genes.

**Table S9:** Results of MR analysis of feature genes and NAFLD.

**Table S10:** Heterogeneity and pleiotropy of MR analysis results.

**Table S11:** Intersection genes of NAFLD and Tet.

**Table S12:** PPI network of Tet in the treatment of NAFLD.

**Table S13:** Go enrichment of Tet in the treatment of NAFLD.

**Table S14:** Kegg pathway of Tet in the treatment of NAFLD.

## Data Availability

The original contributions presented in the study are included in the article/Supporting Information. Supporting Information S1 is available in the supplementary section.
