# Supplementary figures and images for: Tetrandrine, a Major Alkaloid From Stephaniae Tetrandrae Radix, Ameliorates Non‐Alcoholic Fatty Liver Disease in Zebrafish via the PI3K/AKT/STAT3 Pathway
Source: Food Sci Nutr. 2026 May 12;14(5):e71814. doi: 10.1002/fsn3.71814 (PMC13168532; doi:10.1002/fsn3.71814)

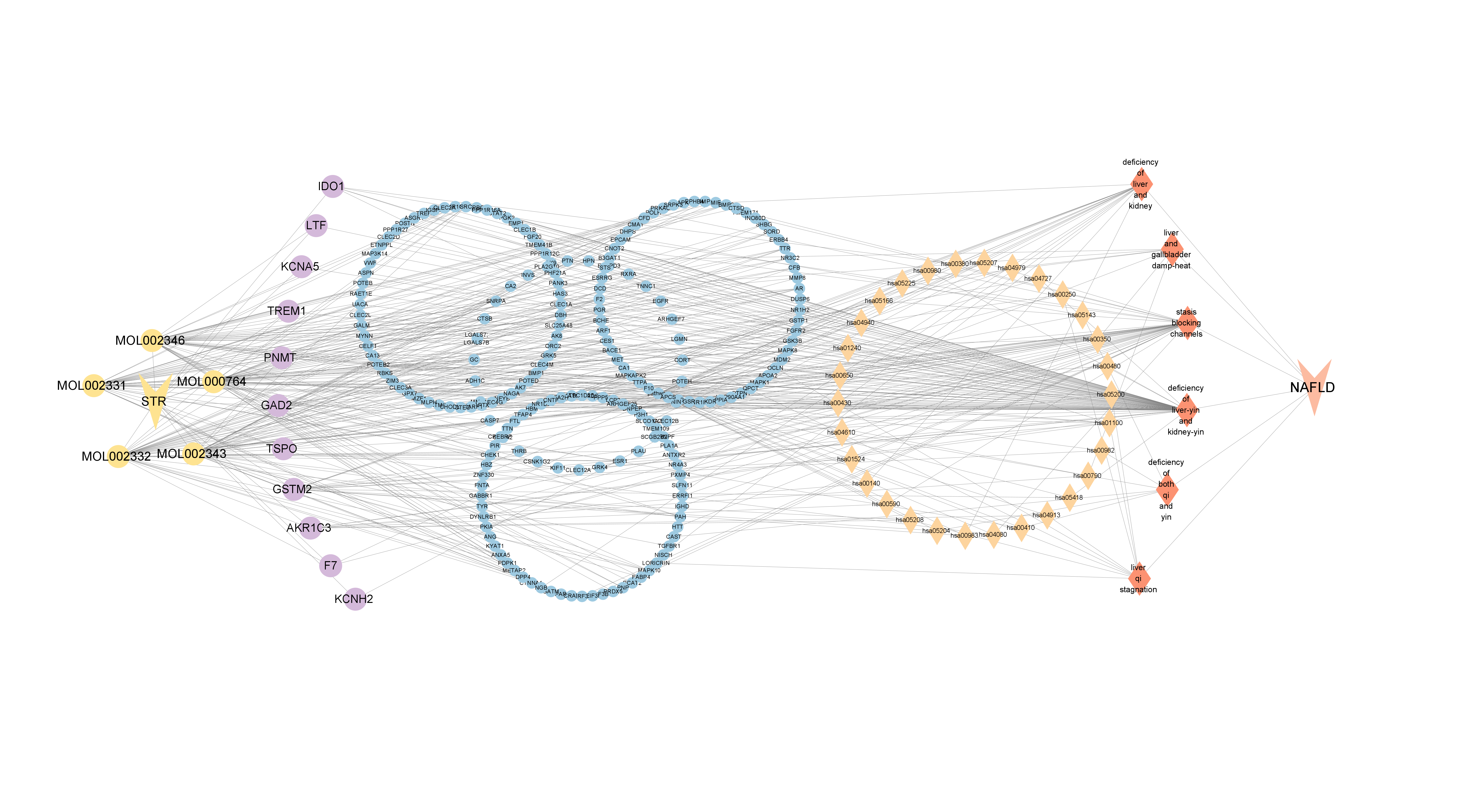


Fig.S1 “STR alkaloids-syndromes-targets-pathways” regulatory network

Supplement: Supplementary file 1 — Figure S1: “STR alkaloids‐syndromes‐targets‐pathways” regulatory network. [file FSN3-14-e71814-s005.docx]
