# Supplementary material for: Tetrandrine, a Major Alkaloid From Stephaniae Tetrandrae Radix, Ameliorates Non‐Alcoholic Fatty Liver Disease in Zebrafish via the PI3K/AKT/STAT3 Pathway
Source: Food Sci Nutr. 2026 May 12;14(5):e71814. doi: 10.1002/fsn3.71814 (PMC13168532; doi:10.1002/fsn3.71814)
Supplement: Supplementary file 8 — Table S7: Pathway enrichment results for MCODEs. [file FSN3-14-e71814-s010.docx]

| Color | MCODE | | Go | | Description | | Log10(P) | |  |
| --- | --- | --- | --- | --- | --- | --- | --- | --- | --- |
|  | MCODE1 | | hsa05200 | | Pathways in cancer | | -22.9 | |  |
|  | MCODE1 | | hsa05163 | | Human cytomegalovirus infection | | -21.0 | |  |
|  | MCODE1 | | hsa05161 | | Hepatitis B | | -20.3 | |  |
|  | MCODE2 | | hsa05200 | | Pathways in cancer | | -22.9 | |  |
|  | MCODE2 | | hsa05167 | | MAPK signaling pathway | | -14.1 | |  |
|  | MCODE2 | | hsa05163 | | Human papillomavirus infection | | -13.7 | |  |
|  | MCODE3 | | hsa05235 | | PD-L1 espression and PD-1checkpoint pathway in cancer | | -11.4 | |  |
|  | MCODE3 | | hsa04920 | | Adipocytokine signaling pathway | | -9.6 | |  |
|  | MCODE3 | | hsa04931 | | Insulin resistance | | -8.7 | |  |
|  | MCODE4 | | hsa05204 | | Chemical carcinogenesis-DNA adducts | | -10.6 | |  |
|  | MCODE4 | | hsa00980 | | Metabolism of xenobiotics by cytochrome P450 | | -10.3 | |  |
|  | MCODE4 | | hsa05207 | | Chemical carcinogenesis-receptor activation | | -10.3 | |  |
| 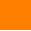 | | MCODE5 | | hsa05169 | | Epstein-Barr virus infection | | -13.2 | |
| 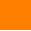 | MCODE5 | | hsa05220 | | Chronic myeloid leukemia | | -10.7 | |  |
| 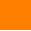 | MCODE5 | | hsa05200 | | Pathways in cancer | | -10.3 | |  |
|  | MCODE6 | | hsa05200 | | Pathways in cancer | | -9.7 | |  |
|  | MCODE6 | | hsa05219 | | Bladder cancer | | -7.1 | |  |
|  | MCODE6 | | hsa05417 | | Lipid and atherosclerosis | | -7.1 | |  |
|  | MCODE7 | | hsa04020 | | Calcium signaling pathway | | -5.6 | |  |
|  | MCODE7 | | hsa04080 | | Neuroactive ligand-receptor interaction | | -5.2 | |  |
|  | MCODE8 | | hsa00071 | | Fatty acid degradation | | -8.0 | |  |
|  | MCODE8 | | hsa00620 | | Pyruvate metabolism | | -7.9 | |  |
|  | MCODE8 | | hsa00010 | | Glycolysis/Gluconeogenesis | | -7.4 | |  |

Table S7 Pathway enrichment results for MCODEs
