# Supplementary material for: Tetrandrine, a Major Alkaloid From Stephaniae Tetrandrae Radix, Ameliorates Non‐Alcoholic Fatty Liver Disease in Zebrafish via the PI3K/AKT/STAT3 Pathway
Source: Food Sci Nutr. 2026 May 12;14(5):e71814. doi: 10.1002/fsn3.71814 (PMC13168532; doi:10.1002/fsn3.71814)
Supplement: Supplementary file 9 — Table S8: SNPs information for the 8 feature genes. [file FSN3-14-e71814-s012.docx]

**Table S8 SNPs information for the 8 feature genes**

| Gene | SNP | A1 | A2 | Chr | n | beta | se | p | eaf | pos.exporsure | R2 | F value |
| --- | --- | --- | --- | --- | --- | --- | --- | --- | --- | --- | --- | --- |
| TP53 | rs35850753 | T | C | 17 | 28529 | -0.356015 | 0.0377475 | 4.04E-21 | 0.0253475 | 7578671 | 0.003108291 | 88.94668332 |
|  | rs57985740 | G | A | 17 | 30902 | 0.080471 | 0.0147073 | 4.46E-08 | 0.205754 | 7413625 | 0.000967845 | 29.93538067 |
| STAT3 | rs1053004 | A | G | 17 | 30755 | 0.205443 | 0.0120967 | 1.09E-64 | 0.612999 | 40466092 | 0.009291356 | 288.4168524 |
|  | rs141688182 | A | G | 17 | 22116 | -0.195379 | 0.0383743 | 3.55E-07 | 0.0246171 | 39775120 | 0.001170737 | 25.92003335 |
|  | rs144904254 | C | T | 17 | 24959 | 0.286758 | 0.0282721 | 3.57E-24 | 0.046124 | 40522158 | 0.004104889 | 102.8679646 |
|  | rs147814995 | T | C | 17 | 18928 | 0.173181 | 0.0337361 | 2.85E-07 | 0.0320964 | 40543581 | 0.001390278 | 26.34904286 |
|  | rs600233 | T | C | 17 | 22757 | 0.098641 | 0.016868 | 4.98E-09 | 0.854688 | 40697793 | 0.001500447 | 34.1939741 |
|  | rs62076368 | G | C | 17 | 30544 | 0.0819616 | 0.0125262 | 6.01E-11 | 0.657867 | 41110630 | 0.001399742 | 42.81083851 |
|  | rs73309793 | C | A | 17 | 30065 | 0.132668 | 0.0158455 | 5.64E-17 | 0.168922 | 40215747 | 0.002326204 | 70.09573231 |
| EGFR | rs10081281 | T | C | 7 | 3301 | 0.132 | 0.0248 | 1.00E-07 | 0.45947 | 55236796 | 0.008509179 | 28.31270031 |
|  | rs10211618 | C | T | 2 | 3301 | -0.1335 | 0.0302 | 9.77E-06 | 0.22052 | 78250631 | 0.005884909 | 19.52924423 |
|  | rs10743150 | A | G | 11 | 3301 | -0.1967 | 0.0438 | 7.08E-06 | 0.9069 | 10824540 | 0.00607253 | 20.15567236 |
|  | rs116846094 | T | C | 17 | 3301 | 0.3427 | 0.0768 | 8.13E-06 | 0.02689 | 58750328 | 0.005995818 | 19.89951813 |
|  | rs116970268 | G | C | 7 | 3301 | -0.2487 | 0.0539 | 3.98E-06 | 0.05766 | 52469472 | 0.006408207 | 21.27702145 |
|  | rs12425000 | T | C | 12 | 3301 | 0.2053 | 0.0452 | 5.50E-06 | 0.08478 | 2044727 | 0.006210832 | 20.61758626 |
|  | rs13262591 | A | G | 8 | 3301 | 0.1215 | 0.0263 | 3.80E-06 | 0.35039 | 144480208 | 0.006423869 | 21.32936126 |
|  | rs1371841 | G | T | 3 | 3301 | 0.1263 | 0.0255 | 7.59E-07 | 0.44515 | 29814715 | 0.007376753 | 24.51676315 |
|  | rs142765860 | C | T | 7 | 3301 | -0.3196 | 0.0669 | 1.82E-06 | 0.0355 | 10494995 | 0.006866311 | 22.80857206 |
|  | rs149521914 | A | G | 5 | 3301 | 0.3341 | 0.0722 | 3.63E-06 | 0.03563 | 136137516 | 0.00644503 | 21.40007756 |
|  | rs17070612 | G | A | 13 | 3301 | 0.214 | 0.0478 | 7.59E-06 | 0.07598 | 79548519 | 0.006035277 | 20.03127276 |
|  | rs4700974 | T | G | 5 | 3301 | -0.1364 | 0.0306 | 8.51E-06 | 0.21555 | 180117474 | 0.005983208 | 19.85741348 |
|  | rs560567196 | A | T | 8 | 3301 | -0.6172 | 0.1313 | 2.63E-06 | 0.01185 | 913690 | 0.006649354 | 22.083056 |
|  | rs62118284 | A | G | 19 | 3301 | 0.3674 | 0.0792 | 3.47E-06 | 0.02439 | 9036338 | 0.006476799 | 21.50625208 |
|  | rs62143197 | A | G | 19 | 3301 | -0.1377 | 0.0304 | 5.89E-06 | 0.22311 | 54320716 | 0.006177094 | 20.50489287 |
|  | rs73922258 | A | C | 19 | 3301 | 0.1752 | 0.0395 | 9.12E-06 | 0.11378 | 8257417 | 0.005924448 | 19.66123543 |
|  | rs757460 | C | G | 16 | 3301 | -0.1219 | 0.0273 | 7.76E-06 | 0.29351 | 367174 | 0.006003733 | 19.92594413 |
|  | rs76494503 | C | T | 7 | 3301 | 0.1895 | 0.0426 | 8.51E-06 | 0.09495 | 9505180 | 0.00595879 | 19.77588927 |
|  | rs79011291 | A | G | 1 | 3301 | 0.3472 | 0.0745 | 3.16E-06 | 0.0273 | 219670203 | 0.00653662 | 21.70619393 |
|  | rs7911360 | A | G | 10 | 3301 | -0.1226 | 0.0274 | 7.76E-06 | 0.38814 | 122805547 | 0.006028486 | 20.00859557 |
|  | rs8013053 | A | G | 14 | 3301 | 0.1915 | 0.0395 | 1.29E-06 | 0.83951 | 75790221 | 0.007069952 | 23.4898453 |
| AKT1 | rs11624762 | T | C | 14 | 26008 | 0.24557 | 0.0180081 | 2.43E-42 | 0.122922 | 105312935 | 0.00709927 | 185.9436856 |
|  | rs149007767 | T | C | 7 | 29114 | 0.129835 | 0.0166458 | 6.20E-15 | 0.149708 | 50370254 | 0.002085287 | 60.83372146 |
|  | rs3809455 | T | G | 14 | 17840 | -0.218295 | 0.0328664 | 3.09E-11 | 0.033836 | 105173831 | 0.002466697 | 44.10975172 |
|  | rs45490496 | T | A | 14 | 22246 | 0.355405 | 0.0116884 | 1.00E-200 | 0.581559 | 105272678 | 0.039902521 | 924.4807853 |
|  | rs4727027 | A | G | 7 | 30222 | -0.0572308 | 0.0123833 | 3.81E-06 | 0.639097 | 148869277 | 0.000706247 | 21.3578781 |
|  | rs55906115 | C | T | 14 | 11786 | 0.0592892 | 0.0128786 | 4.15E-06 | 0.308311 | 105278849 | 0.001795011 | 21.19044948 |
|  | rs7150306 | A | G | 14 | 30274 | 0.0845355 | 0.0156718 | 6.89E-08 | 0.174318 | 105454226 | 0.000960182 | 29.0945603 |
|  | rs72700137 | C | T | 14 | 3802 | -0.30175 | 0.0484676 | 4.78E-10 | 0.0152714 | 105322458 | 0.010091931 | 38.74030286 |
|  | rs77526211 | A | G | 13 | 28314 | -0.148343 | 0.0292445 | 3.92E-07 | 0.0432109 | 40974023 | 0.000907925 | 25.72853371 |
| TNF | rs1121800 | T | A | 6 | 13565 | -0.29148 | 0.0119052 | 2.23E-132 | 0.601256 | 31535074 | 0.042319915 | 599.3494264 |
|  | rs149007767 | T | C | 7 | 13100 | -0.0832278 | 0.0166669 | 5.93E-07 | 0.149708 | 50370254 | 0.001899897 | 24.93221485 |
|  | rs3020644 | G | A | 6 | 13566 | -0.0587203 | 0.0121366 | 1.31E-06 | 0.400462 | 31894626 | 0.00172259 | 23.40552691 |
|  | rs3094672 | A | T | 6 | 7533 | -0.0952462 | 0.0138106 | 5.33E-12 | 0.754758 | 30993377 | 0.00627435 | 47.55047965 |
|  | rs55793580 | T | C | 9 | 14263 | 0.0787324 | 0.0158368 | 6.65E-07 | 0.169838 | 127009395 | 0.001729853 | 24.71218883 |
|  | rs78745236 | G | A | 6 | 4208 | -0.215736 | 0.0475975 | 5.83E-06 | 0.0158648 | 31297237 | 0.00485832 | 20.53385526 |
| CTNNB1 | rs115150816 | C | T | 3 | 28927 | -0.242198 | 0.0293186 | 1.44E-16 | 0.0428476 | 41609265 | 0.002353574 | 68.23773774 |
|  | rs115216619 | T | C | 3 | 23402 | -0.177744 | 0.039426 | 6.53E-06 | 0.023299 | 41147314 | 0.00086775 | 20.32297796 |
|  | rs138284160 | A | G | 3 | 24930 | -0.16472 | 0.0338882 | 1.17E-06 | 0.0318056 | 41488334 | 0.000946808 | 23.62440187 |
|  | rs1722846 | C | T | 3 | 31566 | 0.388782 | 0.0115143 | 1.00E-200 | 0.552754 | 41233889 | 0.034858497 | 1140.012729 |
|  | rs365510 | T | C | 3 | 29992 | 0.0967948 | 0.0118787 | 3.69E-16 | 0.488562 | 41064399 | 0.002209024 | 66.39528868 |
|  | rs56662026 | C | T | 3 | 31160 | 0.432845 | 0.0311491 | 6.72E-44 | 0.0374161 | 41434763 | 0.006158761 | 193.0838404 |
|  | rs62258611 | T | C | 3 | 30902 | -0.113315 | 0.021599 | 1.55E-07 | 0.0826074 | 41805083 | 0.000889886 | 27.52196312 |
|  | rs7637381 | A | G | 3 | 27642 | -0.199872 | 0.0373858 | 8.97E-08 | 0.0259671 | 41205786 | 0.001032933 | 28.57979674 |
|  | rs9813107 | C | T | 3 | 29830 | -0.104168 | 0.0184282 | 1.58E-08 | 0.117996 | 40987921 | 0.001070002 | 31.95020867 |
| BCL2 | rs11152375 | T | C | 18 | 31306 | -0.0947061 | 0.0144365 | 5.37E-11 | 0.783811 | 60906684 | 0.001372802 | 43.03326808 |
|  | rs1375493 | A | G | 2 | 31529 | -0.060327 | 0.0119949 | 4.93E-07 | 0.565077 | 182323766 | 0.000801626 | 25.29313398 |
|  | rs2919917 | C | T | 8 | 31529 | 0.0794717 | 0.0134632 | 3.57E-09 | 0.265373 | 79657263 | 0.001103922 | 34.84182233 |
|  | rs4766578 | A | T | 12 | 28377 | -0.10256 | 0.0118952 | 6.58E-18 | 0.531061 | 111904371 | 0.002612822 | 74.33304393 |
|  | rs4940576 | C | T | 18 | 31528 | 0.0871343 | 0.0136641 | 1.81E-10 | 0.746612 | 60848639 | 0.001288131 | 40.66199467 |
|  | rs6936204 | C | T | 6 | 29787 | -0.0881412 | 0.0125745 | 2.39E-12 | 0.663547 | 32217092 | 0.001646774 | 49.13006135 |
|  | rs76428106 | C | T | 13 | 21035 | -0.248007 | 0.0528102 | 2.65E-06 | 0.0128467 | 28604007 | 0.001047357 | 22.05215107 |
|  | rs78096097 | G | A | 18 | 29734 | -0.19338 | 0.0370757 | 1.83E-07 | 0.0264181 | 60902235 | 0.0009141 | 27.20289844 |
| INS | rs10122243 | T | C | 9 | 435516 | -0.0153627 | 0.00199767 | 5.00E-15 | 0.415773 | 22158924 | 0.000135776 | 59.14058416 |
|  | rs10146997 | G | A | 14 | 435516 | 0.0232609 | 0.00237731 | 2.70E-23 | 0.222146 | 79945162 | 0.000219777 | 95.73690859 |
|  | rs10152546 | A | T | 15 | 435516 | 0.0146048 | 0.00199011 | 2.10E-13 | 0.457724 | 40855210 | 0.000123646 | 53.85612097 |
|  | rs10192766 | G | A | 2 | 435516 | 0.0140013 | 0.00214008 | 1.50E-10 | 0.703061 | 88315993 | 9.8272E-05 | 42.80304948 |
|  | rs10229964 | G | A | 7 | 435516 | 0.017042 | 0.00198052 | 1.20E-17 | 0.427468 | 1051776 | 0.000169983 | 74.04242987 |
|  | rs1042445 | T | C | 3 | 435516 | -0.0189944 | 0.00240272 | 1.50E-16 | 0.219768 | 186395436 | 0.000143476 | 62.49464966 |
|  | rs10426201 | A | G | 19 | 435516 | -0.0240671 | 0.00264574 | 9.10E-20 | 0.833971 | 48384749 | 0.000189962 | 82.74679929 |
|  | rs10431392 | G | A | 12 | 435516 | -0.0216571 | 0.0037413 | 2.00E-08 | 0.07424 | 115103643 | 7.69339E-05 | 33.50838845 |
|  | rs1047743 | G | C | 17 | 435516 | -0.0167665 | 0.00212838 | 1.20E-15 | 0.319304 | 73790093 | 0.000142469 | 62.05610219 |
|  | rs10486064 | T | C | 7 | 435516 | 0.0198359 | 0.00282503 | 6.50E-12 | 0.139487 | 13987644 | 0.000113189 | 49.30099652 |
|  | rs10493008 | A | G | 1 | 435516 | -0.0136153 | 0.00198048 | 9.30E-12 | 0.45121 | 22013771 | 0.000108508 | 47.26193664 |
|  | rs10497015 | C | T | 2 | 435516 | -0.0113025 | 0.00201221 | 4.80E-08 | 0.389781 | 148378069 | 7.24381E-05 | 31.55007755 |
|  | rs10509746 | C | T | 10 | 435516 | 0.0272518 | 0.00198244 | 1.70E-45 | 0.552051 | 102656897 | 0.000433708 | 188.9680092 |
|  | rs1051775 | C | T | 6 | 435516 | -0.0149956 | 0.0019928 | 3.70E-15 | 0.423886 | 52658962 | 0.000129999 | 56.6237035 |
|  | rs10786156 | G | C | 10 | 435516 | -0.0244238 | 0.00200186 | 4.50E-36 | 0.433276 | 96014622 | 0.00034167 | 148.8528218 |
|  | rs1079866 | G | C | 7 | 435516 | 0.019611 | 0.00288173 | 6.60E-13 | 0.133199 | 41470093 | 0.000106327 | 46.31171986 |
|  | rs10832918 | A | G | 11 | 435516 | -0.0206071 | 0.00203679 | 2.40E-26 | 0.624923 | 18335684 | 0.000234982 | 102.3621159 |
|  | rs10835211 | A | G | 11 | 435516 | -0.0123162 | 0.00224141 | 1.90E-08 | 0.260128 | 27701365 | 6.93229E-05 | 30.19318031 |
|  | rs10862960 | T | C | 12 | 435516 | -0.0113695 | 0.0020012 | 8.40E-09 | 0.450535 | 77424135 | 7.41081E-05 | 32.27748955 |
|  | rs10874746 | C | T | 1 | 435516 | -0.0195556 | 0.00205416 | 2.90E-22 | 0.656434 | 93323971 | 0.000208056 | 90.62995414 |
|  | rs10876864 | A | G | 12 | 435516 | 0.0150888 | 0.00198235 | 1.00E-14 | 0.57479 | 56401085 | 0.000133011 | 57.93576414 |
|  | rs10892919 | T | C | 11 | 435516 | 0.0153383 | 0.00198194 | 6.20E-15 | 0.567078 | 122736571 | 0.000137502 | 59.89236404 |
|  | rs10913189 | C | T | 1 | 435516 | -0.0270185 | 0.00396376 | 9.50E-13 | 0.0649132 | 176473979 | 0.000106674 | 46.46284223 |
|  | rs10935299 | A | G | 3 | 435516 | -0.0198198 | 0.00216489 | 1.20E-19 | 0.289723 | 138843870 | 0.000192415 | 83.81560272 |
|  | rs10992828 | A | G | 9 | 435516 | 0.0231374 | 0.00228012 | 1.10E-24 | 0.261062 | 96415368 | 0.000236378 | 102.9702358 |
|  | rs11020842 | A | G | 11 | 435516 | -0.0368386 | 0.00532103 | 1.60E-12 | 0.0354064 | 94317202 | 0.000110043 | 47.9305955 |
|  | rs11042751 | C | T | 11 | 435516 | 0.0705733 | 0.0022946 | 1.00E-200 | 0.242944 | 2149864 | 0.002167307 | 945.9428046 |
|  | rs11073477 | A | G | 15 | 435516 | 0.0141384 | 0.00213826 | 3.30E-10 | 0.308173 | 96885638 | 0.000100376 | 43.719734 |
|  | rs11088472 | C | A | 21 | 435516 | 0.0168008 | 0.00199984 | 2.20E-18 | 0.570687 | 40699376 | 0.00016203 | 70.57768808 |
|  | rs11111274 | A | G | 12 | 435516 | -0.079304 | 0.00221768 | 1.00E-200 | 0.736945 | 102838128 | 0.002927622 | 1278.764091 |
|  | rs11197593 | C | T | 10 | 435516 | 0.0173503 | 0.00197694 | 1.20E-20 | 0.461323 | 117982741 | 0.000176826 | 77.02381139 |
|  | rs112635299 | T | G | 14 | 435516 | -0.07484 | 0.00694303 | 7.20E-28 | 0.0207035 | 94838142 | 0.000266716 | 116.1896597 |
|  | rs1126671 | C | T | 4 | 435516 | 0.0368821 | 0.00214645 | 3.60E-72 | 0.695277 | 100048414 | 0.000677472 | 295.2485238 |
|  | rs113017476 | A | G | 2 | 435516 | -0.0312853 | 0.00504666 | 2.10E-10 | 0.0393039 | 31989359 | 8.82328E-05 | 38.43001552 |
|  | rs113439442 | T | C | 16 | 435516 | -0.0426316 | 0.00406629 | 1.30E-25 | 0.0661062 | 1995997 | 0.000252321 | 109.9169258 |
|  | rs114165349 | C | G | 1 | 435516 | -0.110723 | 0.00646479 | 3.00E-67 | 0.0236245 | 27021913 | 0.000673086 | 293.335652 |
|  | rs11545482 | T | C | 2 | 435516 | -0.0889317 | 0.00707176 | 1.10E-37 | 0.0205017 | 70315987 | 0.000362991 | 158.1452557 |
|  | rs1155397 | G | A | 7 | 435516 | 0.0140983 | 0.00212438 | 2.30E-11 | 0.693717 | 114953597 | 0.000101116 | 44.04200612 |
|  | rs11599690 | C | T | 10 | 435516 | -0.013159 | 0.00215495 | 1.50E-09 | 0.296702 | 32218599 | 8.56111E-05 | 37.28802521 |
|  | rs11603496 | A | G | 11 | 435516 | -0.0157424 | 0.00198017 | 2.20E-16 | 0.541872 | 1576283 | 0.000145101 | 63.20259919 |
|  | rs11618980 | C | T | 13 | 435516 | -0.0146147 | 0.00233164 | 4.10E-10 | 0.234723 | 110354780 | 9.02015E-05 | 39.28753863 |
|  | rs116509476 | C | T | 1 | 435516 | 0.0479914 | 0.00612895 | 2.10E-15 | 0.0260189 | 220693476 | 0.000140763 | 61.31301709 |
|  | rs11691852 | A | C | 2 | 435516 | -0.0136438 | 0.00204484 | 3.40E-11 | 0.643229 | 40609689 | 0.000102212 | 44.51947465 |
|  | rs1171265 | G | A | 1 | 435516 | -0.0183801 | 0.00203721 | 8.60E-19 | 0.644736 | 66003252 | 0.00018687 | 81.39957676 |
|  | rs1171619 | G | A | 10 | 435516 | 0.0142446 | 0.00239419 | 4.10E-10 | 0.784384 | 61465838 | 8.12725E-05 | 35.39820967 |
|  | rs117380693 | A | G | 12 | 435516 | -0.0315616 | 0.00528241 | 1.30E-09 | 0.0357385 | 24377109 | 8.19623E-05 | 35.69865187 |
|  | rs11738977 | G | A | 5 | 435516 | -0.0378122 | 0.00209531 | 5.20E-73 | 0.326955 | 59018442 | 0.000747203 | 325.6606811 |
|  | rs117633128 | A | G | 13 | 435516 | -0.0222713 | 0.00324288 | 3.90E-11 | 0.104983 | 99738554 | 0.000108287 | 47.16581955 |
|  | rs11792865 | C | G | 9 | 435516 | 0.014711 | 0.00274787 | 4.10E-08 | 0.153621 | 19409206 | 6.58051E-05 | 28.66091369 |
|  | rs11794448 | G | A | 9 | 435516 | -0.0191608 | 0.00289785 | 2.40E-11 | 0.13323 | 109995268 | 0.000100376 | 43.71932801 |
|  | rs118073883 | C | T | 16 | 435516 | -0.0303825 | 0.00505066 | 1.00E-09 | 0.0396704 | 1164498 | 8.30827E-05 | 36.18668095 |
|  | rs11809207 | A | G | 1 | 435516 | 0.0238964 | 0.00249494 | 9.00E-24 | 0.188459 | 26521140 | 0.000210596 | 91.73662374 |
|  | rs11862755 | T | C | 16 | 435516 | 0.0892253 | 0.00384275 | 6.00E-123 | 0.0708688 | 1866873 | 0.001236375 | 539.1253795 |
|  | rs11928797 | A | C | 3 | 435516 | 0.0274429 | 0.00306862 | 1.20E-20 | 0.11619 | 33457493 | 0.000183607 | 79.97823013 |
|  | rs12042441 | G | A | 1 | 435516 | 0.0154014 | 0.00284116 | 3.40E-08 | 0.136799 | 244275252 | 6.74677E-05 | 29.38508984 |
|  | rs12118034 | T | C | 1 | 435516 | 0.066352 | 0.00253192 | 2.90E-154 | 0.183228 | 176809368 | 0.001574417 | 686.7617252 |
|  | rs12141189 | C | T | 1 | 435516 | -0.0438658 | 0.00228253 | 1.90E-87 | 0.243974 | 221053545 | 0.000847319 | 369.3321595 |
|  | rs12208357 | T | C | 6 | 435516 | 0.0459497 | 0.0038819 | 1.70E-32 | 0.0700762 | 160543148 | 0.000321612 | 140.1117893 |
|  | rs12210951 | G | A | 6 | 435516 | 0.0163304 | 0.00218586 | 5.10E-14 | 0.279286 | 21936868 | 0.000128141 | 55.81449056 |
|  | rs12253847 | C | T | 10 | 435516 | -0.0126221 | 0.00200597 | 1.70E-10 | 0.409577 | 22859656 | 9.09014E-05 | 39.59244949 |
|  | rs12294104 | T | C | 11 | 435516 | 0.0224008 | 0.00260165 | 1.20E-17 | 0.172766 | 30382899 | 0.000170197 | 74.1356879 |
|  | rs12364884 | A | G | 11 | 435516 | 0.0131334 | 0.00233492 | 1.20E-08 | 0.229476 | 2055968 | 7.26398E-05 | 31.63795023 |
|  | rs12375 | T | C | 1 | 435516 | 0.0174694 | 0.00208614 | 1.10E-15 | 0.328948 | 10596341 | 0.000160989 | 70.12406489 |
|  | rs12432100 | T | C | 14 | 435516 | 0.0215783 | 0.00215813 | 1.00E-25 | 0.701261 | 93871182 | 0.000229496 | 99.97174099 |
|  | rs12454712 | C | T | 18 | 435516 | -0.0160613 | 0.00205101 | 1.60E-15 | 0.374889 | 60845884 | 0.000140786 | 61.32306286 |
|  | rs12550809 | T | A | 8 | 435516 | 0.0107384 | 0.00204424 | 1.50E-08 | 0.365207 | 105178239 | 6.33554E-05 | 27.5939197 |
|  | rs12652907 | G | A | 5 | 435516 | 0.0240425 | 0.00387061 | 4.90E-09 | 0.0702472 | 173376506 | 8.85845E-05 | 38.58321619 |
|  | rs12679822 | G | C | 8 | 435516 | -0.0208674 | 0.00308349 | 2.10E-11 | 0.124032 | 77690380 | 0.000105148 | 45.79832545 |
|  | rs1270231 | A | G | 9 | 435516 | 0.0471593 | 0.00309215 | 1.60E-54 | 0.885471 | 4840877 | 0.000533799 | 232.6010116 |
|  | rs12769257 | C | T | 10 | 435516 | -0.0203593 | 0.00254688 | 1.40E-17 | 0.183347 | 21736100 | 0.000146704 | 63.90086297 |
|  | rs12912439 | T | C | 15 | 435516 | 0.0181273 | 0.00216477 | 1.60E-17 | 0.298667 | 95828705 | 0.000160979 | 70.11980967 |
|  | rs12975366 | C | T | 19 | 435516 | -0.0218929 | 0.00212268 | 1.10E-24 | 0.383132 | 54759361 | 0.00024419 | 106.3740112 |
|  | rs13107325 | T | C | 4 | 435516 | 0.0305647 | 0.0037914 | 1.20E-16 | 0.0748855 | 103188709 | 0.000149201 | 64.98888246 |
|  | rs13210597 | A | C | 6 | 435516 | 0.0173234 | 0.00250802 | 1.40E-11 | 0.189757 | 96969694 | 0.000109535 | 47.70921619 |
|  | rs13232767 | C | A | 7 | 435516 | -0.0125315 | 0.00199835 | 1.20E-09 | 0.601168 | 148659528 | 9.02858E-05 | 39.3243011 |
|  | rs13246732 | T | C | 7 | 435516 | 0.0153344 | 0.00210307 | 1.10E-13 | 0.317735 | 113775765 | 0.000122059 | 53.16479266 |
|  | rs13411546 | A | G | 2 | 435516 | -0.0119759 | 0.00199579 | 2.60E-09 | 0.406331 | 128622491 | 8.26697E-05 | 36.00680967 |
|  | rs1344672 | G | C | 3 | 435516 | -0.0349489 | 0.00197498 | 3.50E-71 | 0.445699 | 141125705 | 0.000718498 | 313.1407762 |
|  | rs1351394 | C | T | 12 | 435516 | 0.0263976 | 0.00196438 | 2.50E-43 | 0.511292 | 66351826 | 0.000414471 | 180.5825933 |
|  | rs1361108 | T | C | 6 | 435516 | 0.0642035 | 0.00197005 | 1.00E-200 | 0.455886 | 126767600 | 0.00243277 | 1062.089012 |
|  | rs1367628 | T | G | 9 | 435516 | 0.0183985 | 0.0025613 | 3.90E-13 | 0.819401 | 74069700 | 0.000118465 | 51.59907792 |
|  | rs1369924 | C | A | 16 | 435516 | -0.0281567 | 0.00253887 | 1.90E-29 | 0.184691 | 5924291 | 0.000282329 | 122.9930539 |
|  | rs146101385 | A | G | 4 | 435516 | -0.0217085 | 0.00376674 | 4.10E-08 | 0.07395 | 15004876 | 7.6259E-05 | 33.21439525 |
|  | rs1471246 | A | G | 10 | 435516 | 0.0164347 | 0.00201598 | 3.60E-15 | 0.604012 | 62074139 | 0.000152574 | 66.45828484 |
|  | rs1495743 | C | G | 8 | 435516 | -0.0280523 | 0.0023726 | 5.60E-34 | 0.778663 | 18273300 | 0.000320881 | 139.7931538 |
|  | rs1497406 | G | A | 1 | 435516 | -0.0181066 | 0.00197553 | 9.30E-21 | 0.579238 | 16505320 | 0.00019285 | 84.004889 |
|  | rs150296 | A | G | 15 | 435516 | 0.0113032 | 0.00203156 | 1.40E-08 | 0.378279 | 89941863 | 7.10736E-05 | 30.95576373 |
|  | rs1512135 | C | T | 4 | 435516 | -0.02302 | 0.00310767 | 4.70E-13 | 0.11236 | 46973888 | 0.000125974 | 54.8704919 |
|  | rs1536241 | G | A | 6 | 435516 | -0.0138594 | 0.00196396 | 1.20E-12 | 0.490908 | 6960310 | 0.000114333 | 49.79911073 |
|  | rs1574525 | G | A | 17 | 435516 | -0.0149602 | 0.00220224 | 1.70E-11 | 0.27711 | 1623675 | 0.000105949 | 46.1470041 |
|  | rs157934 | C | T | 7 | 435516 | 0.0578113 | 0.00213151 | 6.50E-166 | 0.304494 | 130585492 | 0.001686218 | 735.6118684 |
|  | rs1583164 | G | A | 8 | 435516 | 0.0201473 | 0.0020106 | 3.50E-24 | 0.596567 | 77105970 | 0.000230504 | 100.4107835 |
|  | rs1621686 | G | A | 12 | 435516 | 0.0196093 | 0.00215532 | 2.60E-21 | 0.293495 | 32084875 | 0.000190026 | 82.77490087 |
|  | rs165316 | G | A | 1 | 435516 | 0.0732794 | 0.00245603 | 9.59E-198 | 0.197269 | 91533297 | 0.002039884 | 890.2141239 |
|  | rs1678960 | C | T | 12 | 435516 | 0.0157586 | 0.00259899 | 3.30E-10 | 0.171134 | 122264635 | 8.44083E-05 | 36.76410964 |
|  | rs16824937 | A | G | 1 | 435516 | 0.0302009 | 0.00379382 | 4.50E-16 | 0.0714915 | 2182650 | 0.000145485 | 63.3700913 |
|  | rs16873583 | G | T | 6 | 435516 | -0.0157446 | 0.00264406 | 8.90E-10 | 0.164758 | 45593732 | 8.14106E-05 | 35.45836095 |
|  | rs16877774 | C | T | 8 | 435516 | -0.0168289 | 0.00219666 | 1.30E-14 | 0.278395 | 109395713 | 0.000134748 | 58.69265851 |
|  | rs1693551 | C | T | 8 | 435516 | -0.0137831 | 0.0019772 | 2.10E-12 | 0.461757 | 101675584 | 0.000111568 | 48.59489143 |
|  | rs16948305 | T | C | 18 | 435516 | -0.0164622 | 0.00272376 | 3.50E-10 | 0.155857 | 668465 | 8.38682E-05 | 36.52885652 |
|  | rs17012555 | T | C | 1 | 435516 | 0.0319032 | 0.0053551 | 4.70E-11 | 0.0343927 | 208626275 | 8.1488E-05 | 35.4920699 |
|  | rs17050272 | A | G | 2 | 435516 | -0.0213052 | 0.001995 | 1.10E-28 | 0.409945 | 121306440 | 0.000261799 | 114.0468873 |
|  | rs17074673 | G | A | 3 | 435516 | 0.0186221 | 0.0029236 | 3.30E-11 | 0.129712 | 42669393 | 9.31487E-05 | 40.57134525 |
|  | rs17151639 | G | A | 7 | 435516 | 0.0120124 | 0.00219494 | 3.70E-08 | 0.273616 | 127637816 | 6.8767E-05 | 29.95106503 |
|  | rs17274750 | C | A | 21 | 435516 | -0.01882 | 0.00334393 | 2.20E-08 | 0.0971766 | 16353809 | 7.27259E-05 | 31.67545694 |
|  | rs17323117 | G | A | 2 | 435516 | 0.0290604 | 0.00368573 | 1.70E-14 | 0.0761944 | 230162971 | 0.000142722 | 62.16617492 |
|  | rs17400325 | C | T | 2 | 435516 | 0.0607651 | 0.00500995 | 1.60E-35 | 0.041114 | 178565913 | 0.000337669 | 147.1091399 |
|  | rs1740610 | A | C | 1 | 435516 | 0.0226011 | 0.00235593 | 1.00E-21 | 0.779715 | 41502680 | 0.00021127 | 92.03062988 |
|  | rs17428825 | G | A | 13 | 435516 | 0.0122544 | 0.00216012 | 4.90E-09 | 0.299115 | 46522676 | 7.38911E-05 | 32.18298731 |
|  | rs174550 | C | T | 11 | 435516 | -0.0199726 | 0.00206823 | 7.70E-23 | 0.344422 | 61571478 | 0.000214079 | 93.25444623 |
|  | rs17461749 | G | A | 3 | 435516 | -0.0394087 | 0.00738126 | 3.60E-08 | 0.0179697 | 172018312 | 6.54471E-05 | 28.50501123 |
|  | rs17487484 | G | T | 18 | 435516 | -0.0136281 | 0.0019761 | 4.20E-11 | 0.505792 | 50723283 | 0.000109195 | 47.56097977 |
|  | rs175444 | C | G | 14 | 435516 | -0.0180349 | 0.00198674 | 9.50E-20 | 0.46683 | 75601782 | 0.000189173 | 82.40307366 |
|  | rs17577779 | C | T | 14 | 435516 | 0.0219619 | 0.00202398 | 4.30E-28 | 0.484054 | 101200445 | 0.000270275 | 117.7403688 |
|  | rs17585974 | G | T | 17 | 435516 | 0.0185668 | 0.00259785 | 1.30E-13 | 0.192937 | 44249199 | 0.000117271 | 51.07918763 |
|  | rs17616063 | G | A | 16 | 435516 | -0.0273183 | 0.00375274 | 1.30E-13 | 0.074613 | 51436882 | 0.000121662 | 52.99176598 |
|  | rs17714046 | C | T | 5 | 435516 | 0.0377457 | 0.00532797 | 5.90E-13 | 0.0444015 | 180661980 | 0.000115228 | 50.18910275 |
|  | rs1783826 | G | T | 11 | 435516 | 0.011057 | 0.00197978 | 6.60E-10 | 0.541133 | 57402964 | 7.16153E-05 | 31.19167948 |
|  | rs1800574 | T | C | 12 | 435516 | 0.147052 | 0.00588285 | 1.40E-141 | 0.0291911 | 121416864 | 0.001432648 | 624.8335301 |
|  | rs181362 | T | C | 22 | 435516 | -0.0150396 | 0.00250818 | 7.70E-10 | 0.192063 | 21932068 | 8.25496E-05 | 35.95449358 |
|  | rs1832007 | G | A | 10 | 435516 | 0.0589125 | 0.00273166 | 1.40E-109 | 0.153848 | 5254847 | 0.001066827 | 465.1142276 |
|  | rs1861402 | A | G | 2 | 435516 | 0.0197197 | 0.00340887 | 1.40E-09 | 0.908818 | 64879863 | 7.6832E-05 | 33.46399095 |
|  | rs1888223 | A | C | 9 | 435516 | -0.0123915 | 0.00214764 | 1.70E-09 | 0.300229 | 101864974 | 7.64342E-05 | 33.29069024 |
|  | rs1902023 | C | A | 4 | 435516 | -0.026768 | 0.00198716 | 6.70E-44 | 0.473798 | 69536084 | 0.000416468 | 181.4530112 |
|  | rs1942583 | C | T | 18 | 435516 | 0.0155299 | 0.00197527 | 5.20E-15 | 0.525702 | 75198145 | 0.000141912 | 61.81336529 |
|  | rs1956881 | C | A | 14 | 435516 | 0.0119481 | 0.00204144 | 7.10E-09 | 0.598227 | 23394069 | 7.86477E-05 | 34.25488099 |
|  | rs1978903 | T | A | 1 | 435516 | -0.0138113 | 0.00218381 | 3.80E-11 | 0.276604 | 91789628 | 9.18323E-05 | 39.99792139 |
|  | rs1984470 | A | G | 16 | 435516 | 0.0128172 | 0.00197062 | 4.70E-11 | 0.458293 | 53250926 | 9.71257E-05 | 42.30371966 |
|  | rs2023569 | T | C | 6 | 435516 | -0.00954159 | 0.00197538 | 3.70E-08 | 0.442467 | 84292572 | 5.35689E-05 | 23.33126052 |
|  | rs2025258 | C | T | 14 | 435516 | -0.0129092 | 0.00209526 | 3.10E-10 | 0.354552 | 24654489 | 8.71527E-05 | 37.95952943 |
|  | rs2047812 | G | A | 11 | 435516 | -0.041043 | 0.002673 | 4.50E-58 | 0.806867 | 48162042 | 0.000541055 | 235.76443 |
|  | rs2059404 | A | G | 12 | 435516 | 0.0142155 | 0.0019808 | 1.40E-12 | 0.489259 | 46215163 | 0.000118246 | 51.50400841 |
|  | rs2074684 | T | C | 7 | 435516 | 0.0218063 | 0.00293439 | 1.60E-13 | 0.134966 | 100803710 | 0.000126785 | 55.22380195 |
|  | rs2081687 | C | T | 8 | 435516 | -0.0266711 | 0.00208473 | 2.40E-39 | 0.663279 | 59388565 | 0.000375677 | 163.6742008 |
|  | rs2153960 | A | G | 6 | 435516 | 0.0515017 | 0.00216219 | 5.30E-129 | 0.711406 | 108988184 | 0.001301026 | 567.3530922 |
|  | rs2168812 | A | G | 1 | 435516 | -0.0327126 | 0.00254032 | 5.10E-40 | 0.181495 | 243777066 | 0.000380613 | 165.8254854 |
|  | rs2174460 | A | G | 7 | 435516 | -0.0158564 | 0.00228257 | 5.70E-13 | 0.750996 | 55991292 | 0.000110792 | 48.25685132 |
|  | rs2187642 | C | A | 12 | 435516 | -0.0216403 | 0.00202385 | 2.70E-27 | 0.623893 | 11855624 | 0.000262453 | 114.3320308 |
|  | rs2189336 | A | G | 17 | 435516 | 0.0147397 | 0.0019866 | 6.70E-14 | 0.474075 | 5326162 | 0.000126386 | 55.04963351 |
|  | rs2193587 | T | C | 3 | 435516 | -0.0186948 | 0.00242765 | 5.60E-15 | 0.779786 | 185990096 | 0.000136146 | 59.30174937 |
|  | rs2198557 | G | T | 18 | 435516 | 0.0188436 | 0.00214994 | 7.20E-19 | 0.304065 | 1620210 | 0.000176358 | 76.81978357 |
|  | rs2230316 | A | G | 17 | 435516 | -0.0197649 | 0.00205606 | 1.50E-22 | 0.607247 | 17997209 | 0.000212139 | 92.40929993 |
|  | rs2230587 | A | G | 1 | 435516 | 0.0265902 | 0.00303683 | 4.00E-18 | 0.117683 | 65311262 | 0.000176004 | 76.66554924 |
|  | rs2232016 | T | C | 1 | 435516 | 0.0144846 | 0.00240457 | 3.50E-10 | 0.215774 | 107599918 | 8.33101E-05 | 36.28575547 |
|  | rs2234059 | C | T | 22 | 435516 | -0.0323358 | 0.00237745 | 5.60E-45 | 0.224328 | 41777913 | 0.000424576 | 184.9875248 |
|  | rs2235711 | T | C | 6 | 435516 | 0.0202484 | 0.00227072 | 8.20E-20 | 0.751247 | 38148770 | 0.000182545 | 79.51558501 |
|  | rs224331 | C | A | 20 | 435516 | -0.0147758 | 0.00208544 | 1.40E-13 | 0.345288 | 34022387 | 0.000115253 | 50.20010378 |
|  | rs2271976 | C | A | 12 | 435516 | -0.0239931 | 0.0034628 | 1.20E-12 | 0.0891686 | 123751726 | 0.000110221 | 48.00825451 |
|  | rs2274649 | T | A | 9 | 435516 | 0.0154065 | 0.00224516 | 1.80E-12 | 0.263839 | 5090934 | 0.000108109 | 47.08812415 |
|  | rs2277222 | C | T | 10 | 435516 | 0.0157997 | 0.0021702 | 2.50E-15 | 0.300122 | 13494594 | 0.000121686 | 53.00247732 |
|  | rs2280099 | G | A | 4 | 435516 | 0.0264339 | 0.00256982 | 1.00E-26 | 0.178747 | 90035549 | 0.000242889 | 105.8071659 |
|  | rs2288842 | G | C | 19 | 435516 | -0.0181293 | 0.00220811 | 2.80E-18 | 0.276696 | 11031464 | 0.000154757 | 67.40912157 |
|  | rs2292423 | A | T | 4 | 435516 | -0.0115849 | 0.00202346 | 9.30E-10 | 0.406719 | 187175722 | 7.5259E-05 | 32.77882163 |
|  | rs2293889 | G | T | 8 | 435516 | -0.0263509 | 0.00199387 | 1.20E-42 | 0.565715 | 116599199 | 0.000400884 | 174.6607149 |
|  | rs2294239 | G | A | 22 | 435516 | -0.0144081 | 0.00205621 | 2.30E-12 | 0.412422 | 29449477 | 0.000112726 | 49.09943559 |
|  | rs2296198 | C | T | 6 | 435516 | 0.0138723 | 0.00227788 | 1.80E-09 | 0.748842 | 18399750 | 8.51519E-05 | 37.08798743 |
|  | rs2298083 | A | G | 1 | 435516 | -0.0272557 | 0.00298669 | 1.60E-20 | 0.123669 | 183515428 | 0.000191182 | 83.27840345 |
|  | rs2311528 | A | G | 1 | 435516 | 0.0139482 | 0.0021955 | 1.50E-10 | 0.27077 | 17309540 | 9.2667E-05 | 40.36151456 |
|  | rs2323034 | A | G | 6 | 435516 | 0.0356429 | 0.00197856 | 1.10E-74 | 0.433992 | 166306809 | 0.000744595 | 324.5231032 |
|  | rs2395943 | G | A | 6 | 435516 | -0.0121093 | 0.00199068 | 2.60E-10 | 0.587736 | 42940673 | 8.4956E-05 | 37.00267972 |
|  | rs2414095 | G | A | 15 | 435516 | -0.0137421 | 0.00206676 | 6.10E-12 | 0.649305 | 51524292 | 0.000101503 | 44.21036693 |
|  | rs2443728 | C | T | 3 | 435516 | -0.013703 | 0.00251607 | 3.30E-08 | 0.186638 | 11609198 | 6.81008E-05 | 29.66086977 |
|  | rs2488249 | C | T | 1 | 435516 | -0.0182643 | 0.00204601 | 5.30E-19 | 0.350608 | 208000018 | 0.000182939 | 79.68719973 |
|  | rs249624 | T | C | 12 | 435516 | 0.0246123 | 0.00263366 | 5.10E-22 | 0.167249 | 98191847 | 0.00020049 | 87.3339273 |
|  | rs2504063 | G | A | 6 | 435516 | -0.0112482 | 0.00198434 | 4.50E-08 | 0.577013 | 152090707 | 7.3773E-05 | 32.13156593 |
|  | rs2607775 | G | C | 3 | 435516 | 0.0156003 | 0.00196984 | 6.10E-15 | 0.488283 | 14220095 | 0.000143992 | 62.71941539 |
|  | rs2665799 | G | A | 17 | 435516 | -0.0295443 | 0.00211844 | 6.80E-46 | 0.324081 | 61919723 | 0.000446393 | 194.4970769 |
|  | rs2684789 | A | G | 15 | 435516 | -0.0160102 | 0.00198127 | 4.00E-15 | 0.549033 | 99492045 | 0.000149912 | 65.29864852 |
|  | rs2738208 | G | A | 10 | 435516 | 0.01271 | 0.00230027 | 1.90E-08 | 0.75277 | 38306128 | 7.00969E-05 | 30.5303284 |
|  | rs2738787 | G | A | 20 | 435516 | -0.0316348 | 0.00362425 | 9.10E-19 | 0.907587 | 62328375 | 0.00017491 | 76.18893392 |
|  | rs273957 | T | C | 7 | 435516 | -0.0227471 | 0.00202094 | 1.30E-30 | 0.613663 | 137600690 | 0.000290814 | 126.6902635 |
|  | rs2786189 | T | G | 6 | 435516 | -0.0122298 | 0.00196232 | 3.10E-09 | 0.484556 | 147531081 | 8.91777E-05 | 38.84159494 |
|  | rs28399993 | A | G | 6 | 435516 | -0.0884991 | 0.013312 | 5.40E-11 | 0.0130287 | 31732155 | 0.000101471 | 44.19662393 |
|  | rs28410315 | G | A | 9 | 435516 | 0.0284184 | 0.00278141 | 1.30E-26 | 0.146992 | 119338898 | 0.000239641 | 104.3920023 |
|  | rs2854746 | C | G | 7 | 435516 | -0.0464025 | 0.00200876 | 1.90E-121 | 0.39796 | 45960645 | 0.001223744 | 533.6108614 |
|  | rs28551714 | A | G | 4 | 435516 | 0.0287297 | 0.00218758 | 6.50E-42 | 0.280565 | 45131089 | 0.000395875 | 172.4774407 |
|  | rs2855749 | G | A | 12 | 435516 | -0.0113668 | 0.00213383 | 3.30E-08 | 0.302747 | 12046429 | 6.51515E-05 | 28.37623744 |
|  | rs2878298 | C | T | 3 | 435516 | 0.0127527 | 0.00203102 | 3.40E-11 | 0.421376 | 49406080 | 9.05175E-05 | 39.42519881 |
|  | rs2888877 | C | T | 7 | 435516 | 0.0151113 | 0.00245521 | 7.00E-10 | 0.801614 | 92228400 | 8.6973E-05 | 37.88125618 |
|  | rs2893449 | G | A | 7 | 435516 | -0.0251359 | 0.00430421 | 6.10E-09 | 0.945269 | 33026378 | 7.83004E-05 | 34.10358078 |
|  | rs2895168 | G | A | 5 | 435516 | 0.0157575 | 0.00220305 | 1.60E-13 | 0.274903 | 142895518 | 0.000117455 | 51.15922176 |
|  | rs293281 | A | G | 10 | 435516 | 0.0115891 | 0.00209473 | 1.70E-09 | 0.32966 | 53217522 | 7.02763E-05 | 30.60844883 |
|  | rs3008050 | T | C | 6 | 435516 | -0.0115775 | 0.00200737 | 3.50E-09 | 0.394221 | 166064041 | 7.63726E-05 | 33.26386629 |
|  | rs3117234 | G | A | 6 | 435516 | 0.0143138 | 0.00251955 | 5.10E-09 | 0.23898 | 33073984 | 7.41016E-05 | 32.27467907 |
|  | rs3122934 | G | C | 9 | 435516 | -0.015591 | 0.00198434 | 9.80E-16 | 0.439516 | 128154100 | 0.000141726 | 61.73248718 |
|  | rs3136520 | T | C | 11 | 435516 | -0.0404171 | 0.00652967 | 6.70E-10 | 0.0245453 | 46743232 | 8.79641E-05 | 38.31297153 |
|  | rs329120 | T | C | 5 | 435516 | 0.0170407 | 0.0019973 | 2.40E-18 | 0.418347 | 133861756 | 0.000167114 | 72.79243766 |
|  | rs33912345 | A | C | 14 | 435516 | -0.0230144 | 0.00203154 | 2.20E-30 | 0.608461 | 60976537 | 0.000294589 | 128.3354282 |
|  | rs34233878 | C | T | 18 | 435516 | 0.02428 | 0.00332951 | 7.60E-13 | 0.0976325 | 42594280 | 0.00012209 | 53.17833349 |
|  | rs34312198 | A | C | 7 | 435516 | 0.0178553 | 0.0031566 | 6.40E-09 | 0.110684 | 99674870 | 7.34614E-05 | 31.995817 |
|  | rs34348385 | G | C | 7 | 435516 | -0.0472804 | 0.00284491 | 2.50E-64 | 0.136857 | 6717416 | 0.000633791 | 276.199715 |
|  | rs343964 | G | A | 2 | 435516 | 0.0143477 | 0.00264383 | 3.30E-09 | 0.165226 | 44966378 | 6.76183E-05 | 29.45069362 |
|  | rs34536443 | C | G | 19 | 435516 | -0.0543907 | 0.00485758 | 2.80E-29 | 0.0459052 | 10463118 | 0.000287793 | 125.3739701 |
|  | rs34631447 | G | A | 3 | 435516 | 0.0119902 | 0.00200578 | 1.10E-09 | 0.398327 | 188472322 | 8.20439E-05 | 35.73421673 |
|  | rs34767 | G | T | 5 | 435516 | 0.0154624 | 0.00220482 | 5.40E-13 | 0.286541 | 102489511 | 0.000112916 | 49.18192622 |
|  | rs34967399 | C | T | 2 | 435516 | 0.0412141 | 0.00701016 | 5.30E-09 | 0.0200258 | 28357730 | 7.93592E-05 | 34.56477911 |
|  | rs35041525 | C | G | 3 | 435516 | -0.0172219 | 0.00306667 | 2.80E-08 | 0.115996 | 14414455 | 7.2409E-05 | 31.53741133 |
|  | rs35243581 | T | C | 11 | 435516 | -0.0117094 | 0.00211268 | 1.60E-08 | 0.31712 | 43707364 | 7.05289E-05 | 30.71848886 |
|  | rs357269 | A | G | 5 | 435516 | -0.0138387 | 0.00263406 | 2.90E-08 | 0.16652 | 38877564 | 6.33735E-05 | 27.60179133 |
|  | rs35740221 | G | T | 18 | 435516 | -0.0115504 | 0.00202952 | 1.40E-08 | 0.386158 | 31387714 | 7.43654E-05 | 32.38958501 |
|  | rs36000545 | G | A | 17 | 435516 | -0.0177863 | 0.00206481 | 2.50E-19 | 0.39416 | 79093822 | 0.000170346 | 74.20087769 |
|  | rs36023044 | T | C | 10 | 435516 | 0.0222258 | 0.0027607 | 3.10E-15 | 0.149768 | 125083220 | 0.000148802 | 64.81482893 |
|  | rs36023504 | T | C | 4 | 435516 | -0.0151361 | 0.00204916 | 4.10E-14 | 0.382075 | 38698924 | 0.000125262 | 54.55998506 |
|  | rs362285 | A | G | 4 | 435516 | -0.0574356 | 0.0079984 | 7.10E-15 | 0.0153343 | 3258321 | 0.000118386 | 51.56488949 |
|  | rs364199 | G | A | 21 | 435516 | 0.0145758 | 0.00204102 | 3.90E-12 | 0.597704 | 42818132 | 0.000117089 | 50.99977803 |
|  | rs3738951 | G | A | 2 | 435516 | 0.013786 | 0.00199146 | 7.70E-12 | 0.442283 | 225368321 | 0.000110022 | 47.92160759 |
|  | rs3745683 | A | G | 19 | 435516 | 0.0277232 | 0.00379148 | 7.30E-13 | 0.0737624 | 11348521 | 0.000122747 | 53.46470598 |
|  | rs3752416 | C | T | 6 | 435516 | -0.0259787 | 0.00228632 | 1.10E-29 | 0.692845 | 26045929 | 0.000296366 | 129.1096799 |
|  | rs3755304 | T | C | 2 | 435516 | -0.0235201 | 0.00291913 | 8.10E-18 | 0.129801 | 42589921 | 0.00014904 | 64.91864716 |
|  | rs3761706 | A | G | 2 | 435516 | 0.0280505 | 0.00404317 | 4.00E-13 | 0.0630121 | 111899881 | 0.000110506 | 48.13214487 |
|  | rs3791679 | G | A | 2 | 435516 | 0.0181918 | 0.00233842 | 1.70E-15 | 0.226857 | 56096892 | 0.000138945 | 60.52075225 |
|  | rs3809627 | A | C | 16 | 435516 | -0.0163347 | 0.00200713 | 1.90E-16 | 0.402174 | 30103160 | 0.000152055 | 66.23222219 |
|  | rs3812316 | G | C | 7 | 435516 | 0.0368404 | 0.00293799 | 2.00E-37 | 0.127549 | 73020337 | 0.0003609 | 157.2338516 |
|  | rs3832685 | GCTC | G | 10 | 435516 | -0.01582 | 0.00207628 | 4.80E-14 | 0.344848 | 104629793 | 0.000133284 | 58.05493227 |
|  | rs3850805 | A | G | 18 | 435516 | 0.0142514 | 0.00229619 | 2.90E-11 | 0.754747 | 3817613 | 8.84417E-05 | 38.52098846 |
|  | rs3912391 | A | G | 4 | 435516 | -0.0291086 | 0.00196646 | 8.70E-50 | 0.507111 | 39698824 | 0.000502863 | 219.1141419 |
|  | rs397969 | C | T | 17 | 435516 | -0.0118884 | 0.00201407 | 2.10E-09 | 0.397962 | 19804247 | 7.99943E-05 | 34.84140842 |
|  | rs4073455 | G | T | 8 | 435516 | 0.0133688 | 0.00199774 | 2.10E-11 | 0.57612 | 144978607 | 0.000102815 | 44.78214865 |
|  | rs41288985 | C | G | 6 | 435516 | -0.0209204 | 0.00371384 | 2.60E-09 | 0.0757227 | 100016340 | 7.28547E-05 | 31.7315725 |
|  | rs4144501 | A | G | 12 | 435516 | 0.015406 | 0.00196412 | 2.70E-14 | 0.51266 | 94156587 | 0.000141247 | 61.52360239 |
|  | rs41514145 | T | G | 4 | 435516 | 0.0120853 | 0.00228685 | 4.60E-08 | 0.244624 | 178965658 | 6.41221E-05 | 27.92785099 |
|  | rs4234798 | G | T | 4 | 435516 | 0.0377704 | 0.00202047 | 3.20E-80 | 0.615908 | 7219933 | 0.000801763 | 349.4591056 |
|  | rs424272 | T | C | 4 | 435516 | 0.0129411 | 0.00228335 | 2.80E-10 | 0.754306 | 124024947 | 7.37499E-05 | 32.12147047 |
|  | rs4245150 | T | G | 11 | 435516 | -0.0140118 | 0.00201937 | 5.10E-12 | 0.619963 | 113364647 | 0.000110536 | 48.1453186 |
|  | rs4282339 | A | G | 5 | 435516 | -0.051088 | 0.00242003 | 2.00E-105 | 0.207441 | 168256240 | 0.001022229 | 445.650408 |
|  | rs445036 | C | T | 8 | 435516 | -0.017862 | 0.00217062 | 2.30E-17 | 0.290271 | 81408409 | 0.000155461 | 67.71588755 |
|  | rs4468717 | T | C | 18 | 435516 | -0.0275171 | 0.00386422 | 5.80E-14 | 0.0766855 | 3457606 | 0.00011642 | 50.70836899 |
|  | rs466037 | C | T | 9 | 435516 | -0.0145004 | 0.00207266 | 5.70E-12 | 0.349588 | 136875263 | 0.00011237 | 48.94426811 |
|  | rs4675814 | G | T | 2 | 435516 | 0.0148231 | 0.00239021 | 4.20E-10 | 0.787189 | 242388463 | 8.83006E-05 | 38.45952928 |
|  | rs4678144 | G | A | 3 | 435516 | 0.0176626 | 0.00244715 | 1.00E-13 | 0.201184 | 124404399 | 0.0001196 | 52.09380641 |
|  | rs4678732 | A | G | 3 | 435516 | 0.0154845 | 0.00202921 | 1.70E-15 | 0.373304 | 33212485 | 0.000133684 | 58.22887382 |
|  | rs4683324 | C | T | 3 | 435516 | 0.0202224 | 0.00198934 | 6.30E-26 | 0.579488 | 47251284 | 0.000237214 | 103.3345062 |
|  | rs4687612 | G | A | 3 | 435516 | 0.0191391 | 0.0028222 | 2.30E-12 | 0.140644 | 52367932 | 0.000105589 | 45.99021647 |
|  | rs4705873 | A | G | 5 | 435516 | -0.0382759 | 0.00264797 | 3.50E-51 | 0.164995 | 132267167 | 0.000479526 | 208.9405373 |
|  | rs4743034 | A | G | 9 | 435516 | 0.0200938 | 0.00235977 | 4.40E-18 | 0.224586 | 109632353 | 0.000166459 | 72.50747891 |
|  | rs4833676 | G | C | 4 | 435516 | 0.014029 | 0.00212169 | 2.20E-11 | 0.314778 | 121720941 | 0.000100379 | 43.72074736 |
|  | rs4872203 | G | A | 8 | 435516 | 0.0145335 | 0.00209028 | 1.30E-12 | 0.667045 | 23734849 | 0.000110989 | 48.34254444 |
|  | rs4888156 | C | T | 16 | 435516 | 0.0297713 | 0.00207494 | 2.30E-47 | 0.659686 | 81604256 | 0.000472471 | 205.8650006 |
|  | rs4895842 | C | T | 6 | 435516 | -0.0140588 | 0.00211071 | 1.30E-11 | 0.68455 | 129349539 | 0.000101857 | 44.36468227 |
|  | rs4897242 | G | A | 6 | 435516 | 0.0163434 | 0.00262915 | 5.60E-11 | 0.827542 | 128388990 | 8.87179E-05 | 38.64133274 |
|  | rs4938799 | G | T | 11 | 435516 | 0.0168137 | 0.00202763 | 3.10E-16 | 0.381991 | 120208257 | 0.000157862 | 68.76179061 |
|  | rs4988501 | G | A | 7 | 435516 | -0.0210058 | 0.00233465 | 2.60E-21 | 0.259103 | 31011485 | 0.000185845 | 80.95298985 |
|  | rs505922 | C | T | 9 | 435516 | 0.0135684 | 0.00211998 | 3.70E-11 | 0.317355 | 136149229 | 9.40478E-05 | 40.9629999 |
|  | rs532964 | G | A | 5 | 435516 | 0.0151958 | 0.00197514 | 6.50E-15 | 0.519634 | 78340286 | 0.00013589 | 59.19014105 |
|  | rs5398 | A | G | 3 | 435516 | 0.0229408 | 0.0021764 | 1.00E-25 | 0.285527 | 170715830 | 0.00025505 | 111.106045 |
|  | rs55707100 | T | C | 15 | 435516 | -0.145514 | 0.00627484 | 1.80E-123 | 0.0255668 | 43820717 | 0.001233287 | 537.7770334 |
|  | rs55714927 | T | C | 17 | 435516 | 0.0198101 | 0.00253847 | 5.40E-16 | 0.190136 | 7080316 | 0.000139818 | 60.90139923 |
|  | rs55843942 | G | C | 1 | 435516 | -0.0219817 | 0.00294144 | 2.20E-15 | 0.126845 | 214219331 | 0.000128216 | 55.84709283 |
|  | rs55922628 | A | C | 10 | 435516 | -0.012306 | 0.00203269 | 2.00E-09 | 0.609205 | 77191441 | 8.41494E-05 | 36.65131202 |
|  | rs56145419 | A | G | 1 | 435516 | 0.0176149 | 0.00316705 | 5.30E-09 | 0.106693 | 179379405 | 7.10257E-05 | 30.93488671 |
|  | rs56206519 | A | G | 17 | 435516 | 0.0110959 | 0.002018 | 2.40E-08 | 0.400556 | 7303143 | 6.94142E-05 | 30.2329656 |
|  | rs56293839 | T | C | 4 | 435516 | -0.0155779 | 0.00263938 | 4.90E-10 | 0.166381 | 146797543 | 7.99788E-05 | 34.83469288 |
|  | rs56324928 | C | A | 9 | 435516 | 0.0217317 | 0.0023902 | 7.00E-21 | 0.217087 | 33933136 | 0.000189772 | 82.66409656 |
|  | rs56344540 | T | C | 20 | 435516 | -0.0450315 | 0.00360405 | 2.70E-37 | 0.0815877 | 49194444 | 0.000358337 | 156.116648 |
|  | rs572169 | T | C | 3 | 435516 | 0.054113 | 0.00213954 | 1.00E-144 | 0.313462 | 172165727 | 0.001466631 | 639.676518 |
|  | rs5742915 | C | T | 15 | 435516 | 0.02217 | 0.00202449 | 4.50E-28 | 0.456324 | 74336633 | 0.000275281 | 119.9217949 |
|  | rs5750202 | T | C | 22 | 435516 | 0.0268809 | 0.00291858 | 9.70E-22 | 0.131951 | 36339542 | 0.00019474 | 84.82862196 |
|  | rs5751777 | T | C | 22 | 435516 | -0.0181328 | 0.00200631 | 3.60E-20 | 0.586306 | 24267047 | 0.00018752 | 81.68299868 |
|  | rs5758896 | C | T | 22 | 435516 | 0.0108049 | 0.00201327 | 1.50E-08 | 0.594591 | 43115576 | 6.61309E-05 | 28.80285015 |
|  | rs585187 | T | G | 18 | 435516 | 0.0152522 | 0.00197716 | 1.50E-14 | 0.493232 | 58177124 | 0.000136621 | 59.50854849 |
|  | rs5899258 | GC | G | 9 | 435516 | -0.0180986 | 0.00206864 | 1.70E-19 | 0.365177 | 98218474 | 0.000175727 | 76.54523009 |
|  | rs595657 | A | G | 18 | 435516 | -0.0117734 | 0.0020991 | 1.10E-08 | 0.329817 | 41002846 | 7.22274E-05 | 31.45832154 |
|  | rs599839 | A | G | 1 | 435516 | -0.031267 | 0.00232644 | 3.30E-40 | 0.771969 | 109822166 | 0.000414576 | 180.628685 |
|  | rs6037513 | C | T | 20 | 435516 | 0.0133671 | 0.00200451 | 1.10E-11 | 0.586414 | 3223225 | 0.000102096 | 44.46885481 |
|  | rs60789653 | A | G | 20 | 435516 | -0.0263098 | 0.00265903 | 3.10E-23 | 0.170977 | 33447162 | 0.000224743 | 97.90088655 |
|  | rs6082354 | C | A | 20 | 435516 | -0.0446663 | 0.00209406 | 1.80E-106 | 0.667702 | 21217976 | 0.001043576 | 454.9668147 |
|  | rs60849954 | T | C | 14 | 435516 | -0.0246761 | 0.00427618 | 1.20E-09 | 0.0579201 | 73245733 | 7.64546E-05 | 33.29960583 |
|  | rs609385 | T | C | 5 | 435516 | -0.0208107 | 0.00198231 | 3.70E-27 | 0.430542 | 134597267 | 0.000252997 | 110.2118365 |
|  | rs6127684 | A | G | 20 | 435516 | 0.0181014 | 0.0028197 | 4.00E-11 | 0.142439 | 54809462 | 9.46179E-05 | 41.21132024 |
|  | rs61740705 | G | A | 4 | 435516 | 0.0190662 | 0.00249387 | 8.10E-16 | 0.199222 | 169343044 | 0.000134189 | 58.44921362 |
|  | rs61742747 | A | G | 16 | 435516 | -0.0766027 | 0.00621056 | 1.70E-34 | 0.0279251 | 1961866 | 0.000349197 | 152.1334229 |
|  | rs61957204 | A | G | 13 | 435516 | 0.0345879 | 0.00367397 | 8.90E-22 | 0.0785484 | 74084684 | 0.000203463 | 88.62886415 |
|  | rs62012820 | T | G | 15 | 435516 | -0.0158887 | 0.0025467 | 2.60E-10 | 0.18596 | 63908587 | 8.93672E-05 | 38.92415194 |
|  | rs62052820 | A | G | 16 | 435516 | -0.0245088 | 0.00234902 | 2.00E-24 | 0.226959 | 69575238 | 0.000249895 | 108.8600858 |
|  | rs62127750 | C | A | 2 | 435516 | 0.0298491 | 0.0045598 | 6.80E-12 | 0.048645 | 26130680 | 9.83839E-05 | 42.8517832 |
|  | rs632616 | T | C | 19 | 435516 | -0.0395989 | 0.00212434 | 2.80E-84 | 0.312676 | 4976335 | 0.000797201 | 347.4691055 |
|  | rs6431786 | C | T | 2 | 435516 | -0.0199933 | 0.00237089 | 2.70E-18 | 0.219316 | 6399104 | 0.000163257 | 71.11220761 |
|  | rs6447962 | A | G | 4 | 435516 | 0.0156898 | 0.00222892 | 5.60E-12 | 0.264874 | 20432533 | 0.000113761 | 49.55002279 |
|  | rs6452873 | G | A | 5 | 435516 | 0.0122476 | 0.00213872 | 3.10E-09 | 0.302209 | 89456841 | 7.52935E-05 | 32.79382935 |
|  | rs6461071 | C | T | 7 | 435516 | -0.0177711 | 0.00216132 | 3.70E-18 | 0.288634 | 14246698 | 0.00015521 | 67.60650459 |
|  | rs6471133 | C | A | 8 | 435516 | 0.0279382 | 0.00246476 | 1.10E-29 | 0.199631 | 134611160 | 0.000294928 | 128.4829723 |
|  | rs6486122 | T | C | 11 | 435516 | 0.0211515 | 0.00212186 | 3.20E-23 | 0.689178 | 13361524 | 0.000228111 | 99.36807973 |
|  | rs6498697 | G | A | 16 | 435516 | -0.0146271 | 0.00196279 | 1.90E-14 | 0.494968 | 17479794 | 0.0001275 | 55.53500218 |
|  | rs6510033 | G | A | 19 | 435516 | -0.0184607 | 0.00220966 | 6.50E-19 | 0.274319 | 30710785 | 0.00016024 | 69.7980721 |
|  | rs6510959 | A | G | 19 | 435516 | -0.020444 | 0.00277293 | 7.50E-12 | 0.155337 | 7184238 | 0.000124795 | 54.35655665 |
|  | rs6519133 | C | T | 22 | 435516 | -0.0294392 | 0.00203003 | 5.30E-47 | 0.393838 | 39096602 | 0.000482651 | 210.3028226 |
|  | rs6534673 | T | C | 4 | 435516 | 0.0166387 | 0.00219232 | 7.10E-15 | 0.720095 | 129142166 | 0.000132242 | 57.60085059 |
|  | rs6535413 | G | A | 4 | 435516 | -0.0143568 | 0.00199546 | 4.60E-13 | 0.435007 | 83857108 | 0.000118843 | 51.76393144 |
|  | rs655555 | A | C | 1 | 435516 | 0.0117711 | 0.00202432 | 1.50E-09 | 0.370872 | 234863292 | 7.76315E-05 | 33.81222671 |
|  | rs66550728 | G | A | 12 | 435516 | 0.0531625 | 0.00277097 | 1.30E-83 | 0.146777 | 31948210 | 0.000844454 | 368.0824824 |
|  | rs6659633 | A | G | 1 | 435516 | 0.0153961 | 0.00272128 | 3.10E-08 | 0.151651 | 68735312 | 7.34918E-05 | 32.0090653 |
|  | rs66699565 | G | A | 1 | 435516 | -0.0138332 | 0.00217739 | 1.80E-11 | 0.278641 | 28701455 | 9.26677E-05 | 40.36183192 |
|  | rs66720010 | G | A | 16 | 435516 | -0.0242669 | 0.00203382 | 4.70E-33 | 0.372321 | 83979322 | 0.000326782 | 142.364458 |
|  | rs668871 | T | C | 6 | 435516 | -0.0332842 | 0.00197269 | 2.10E-68 | 0.469452 | 160769811 | 0.000653237 | 284.6797432 |
|  | rs6798156 | G | T | 3 | 435516 | -0.013957 | 0.00215334 | 2.00E-10 | 0.295198 | 187596315 | 9.64524E-05 | 42.0104126 |
|  | rs68013747 | A | G | 8 | 435516 | 0.0203604 | 0.0021329 | 5.50E-21 | 0.307446 | 135656665 | 0.000209188 | 91.12333981 |
|  | rs6805889 | G | T | 3 | 435516 | -0.0134245 | 0.00195994 | 1.10E-11 | 0.518104 | 23389682 | 0.000107711 | 46.91467274 |
|  | rs68176600 | G | A | 12 | 435516 | -0.0146957 | 0.00208645 | 5.90E-13 | 0.329135 | 57618419 | 0.000113897 | 49.60924271 |
|  | rs684773 | C | A | 3 | 435516 | 0.0420925 | 0.00231982 | 7.30E-78 | 0.766785 | 135956305 | 0.000755385 | 329.2295968 |
|  | rs6879346 | G | A | 5 | 435516 | 0.0159945 | 0.00299063 | 1.80E-08 | 0.877061 | 162966787 | 6.56725E-05 | 28.60315707 |
|  | rs6934603 | G | A | 6 | 435516 | -0.0287109 | 0.00196732 | 5.70E-50 | 0.485716 | 87994015 | 0.000488795 | 212.9813642 |
|  | rs6964426 | A | G | 7 | 435516 | 0.0363569 | 0.00616791 | 3.30E-09 | 0.0259245 | 46640191 | 7.97735E-05 | 34.745266 |
|  | rs700752 | G | C | 7 | 435516 | 0.118622 | 0.00205628 | 1.00E-200 | 0.653068 | 46753553 | 0.00758326 | 3327.851872 |
|  | rs7072243 | A | G | 10 | 435516 | 0.0120007 | 0.00201703 | 3.80E-08 | 0.392866 | 50319387 | 8.12735E-05 | 35.39862954 |
|  | rs7174384 | A | C | 15 | 435516 | -0.0391857 | 0.00427882 | 4.50E-21 | 0.0566395 | 68606098 | 0.000192539 | 83.86971938 |
|  | rs7178424 | T | C | 15 | 435516 | -0.0376273 | 0.00198557 | 1.30E-85 | 0.448212 | 62380259 | 0.000823898 | 359.1151381 |
|  | rs7197580 | A | G | 16 | 435516 | -0.0119476 | 0.00197236 | 3.40E-10 | 0.484283 | 19278625 | 8.42458E-05 | 36.69331772 |
|  | rs7261425 | G | C | 20 | 435516 | -0.0507684 | 0.00220431 | 4.60E-125 | 0.2784 | 20068635 | 0.001216491 | 530.4440924 |
|  | rs72631343 | G | C | 17 | 435516 | -0.0179863 | 0.00294146 | 1.80E-09 | 0.128776 | 67191270 | 8.58453E-05 | 37.39002689 |
|  | rs727428 | C | T | 17 | 435516 | 0.0182778 | 0.00198468 | 2.20E-20 | 0.556472 | 7537792 | 0.000194705 | 84.81347563 |
|  | rs72793809 | T | C | 16 | 435516 | 0.0173673 | 0.00200615 | 1.90E-18 | 0.401521 | 28832382 | 0.000172052 | 74.94381792 |
|  | rs72799666 | T | C | 2 | 435516 | 0.056097 | 0.00527721 | 4.70E-27 | 0.0358512 | 25992902 | 0.00025939 | 112.9974193 |
|  | rs72841131 | C | T | 2 | 435516 | 0.0323549 | 0.00398469 | 5.60E-17 | 0.0647352 | 70414741 | 0.000151364 | 65.93090689 |
|  | rs72885919 | T | C | 2 | 435516 | 0.0120839 | 0.00201519 | 2.20E-09 | 0.385023 | 172418755 | 8.25548E-05 | 35.95673522 |
|  | rs7306772 | G | T | 12 | 435516 | -0.0141462 | 0.00217068 | 2.80E-10 | 0.714424 | 89914142 | 9.75083E-05 | 42.47036405 |
|  | rs7315980 | A | G | 12 | 435516 | 0.0202712 | 0.00354445 | 2.30E-08 | 0.0843069 | 53449321 | 7.50973E-05 | 32.7083935 |
|  | rs73238159 | T | C | 3 | 435516 | -0.0282502 | 0.00301026 | 4.80E-21 | 0.124276 | 142078759 | 0.000202182 | 88.07102354 |
|  | rs73530203 | A | G | 16 | 435516 | -0.0307402 | 0.00209334 | 3.40E-51 | 0.365614 | 31099859 | 0.000494897 | 215.6412491 |
|  | rs74400046 | A | G | 7 | 435516 | 0.0641896 | 0.00826378 | 2.40E-15 | 0.0144497 | 46636896 | 0.000138518 | 60.33507414 |
|  | rs747854 | A | G | 14 | 435516 | -0.0136172 | 0.00203493 | 2.50E-11 | 0.378423 | 69233836 | 0.000102808 | 44.77903075 |
|  | rs7489210 | T | C | 12 | 435516 | 0.0176173 | 0.00282676 | 7.80E-11 | 0.139819 | 20585303 | 8.91781E-05 | 38.84175378 |
|  | rs75218626 | T | C | 12 | 435516 | 0.0533349 | 0.00538607 | 1.90E-25 | 0.034257 | 111429783 | 0.000225101 | 98.0566358 |
|  | rs75403087 | A | G | 19 | 435516 | -0.023024 | 0.00339403 | 1.10E-12 | 0.0930136 | 39147510 | 0.000105653 | 46.01804933 |
|  | rs75524443 | A | G | 1 | 435516 | 0.0261416 | 0.00446898 | 1.40E-09 | 0.0505729 | 153792297 | 7.85614E-05 | 34.21728237 |
|  | rs7572505 | A | G | 2 | 435516 | -0.0194358 | 0.00229401 | 5.90E-18 | 0.24225 | 203152355 | 0.000164793 | 71.78145132 |
|  | rs757647 | A | G | 5 | 435516 | 0.038841 | 0.00243993 | 4.20E-57 | 0.203718 | 137707315 | 0.000581526 | 253.4101916 |
|  | rs76026733 | C | T | 5 | 435516 | 0.0382706 | 0.0043961 | 5.30E-19 | 0.0527775 | 53263963 | 0.000173987 | 75.78677416 |
|  | rs7607369 | G | A | 2 | 435516 | 0.0166638 | 0.0019782 | 8.10E-18 | 0.567036 | 219279097 | 0.000162904 | 70.95870804 |
|  | rs762360 | T | C | 21 | 435516 | 0.0409274 | 0.0021635 | 6.30E-83 | 0.695849 | 37425955 | 0.00082102 | 357.8594735 |
|  | rs763157 | A | C | 6 | 435516 | -0.0224474 | 0.00297289 | 7.40E-14 | 0.870959 | 35258539 | 0.000130892 | 57.01280601 |
|  | rs765250 | T | C | 12 | 435516 | 0.0127929 | 0.00213076 | 2.10E-09 | 0.694 | 908283 | 8.27616E-05 | 36.04681894 |
|  | rs76708468 | C | T | 17 | 435516 | 0.0893033 | 0.00512662 | 1.20E-69 | 0.0384512 | 62206299 | 0.000696252 | 303.438558 |
|  | rs76750172 | T | C | 13 | 435516 | 0.0660123 | 0.00645094 | 1.10E-25 | 0.0241381 | 28395297 | 0.000240378 | 104.7132719 |
|  | rs7697204 | T | C | 4 | 435516 | 0.0240998 | 0.00224949 | 8.50E-29 | 0.742076 | 148980174 | 0.000263475 | 114.7774968 |
|  | rs77107022 | T | C | 13 | 435516 | 0.0301452 | 0.00580325 | 2.90E-08 | 0.0308409 | 21955635 | 6.19531E-05 | 26.98309638 |
|  | rs77169818 | T | A | 18 | 435516 | -0.0585355 | 0.00495376 | 1.40E-32 | 0.0417998 | 74980601 | 0.000320498 | 139.6261446 |
|  | rs7723160 | G | A | 5 | 435516 | 0.020246 | 0.00224282 | 1.40E-20 | 0.740906 | 42860681 | 0.00018707 | 81.48686884 |
|  | rs7742369 | G | A | 6 | 435516 | -0.0263862 | 0.00258092 | 2.20E-26 | 0.175058 | 34165721 | 0.000239936 | 104.5207745 |
|  | rs77542162 | G | A | 17 | 435516 | 0.0437292 | 0.00675426 | 8.20E-12 | 0.0227081 | 67081278 | 9.62369E-05 | 41.91654913 |
|  | rs7759938 | T | C | 6 | 435516 | 0.0189059 | 0.00209934 | 6.20E-21 | 0.678486 | 105378954 | 0.000186185 | 81.10117699 |
|  | rs77666550 | C | T | 2 | 435516 | 0.0665311 | 0.00819825 | 3.00E-16 | 0.0151515 | 26052252 | 0.000151195 | 65.85747841 |
|  | rs7789908 | G | T | 7 | 435516 | 0.0197385 | 0.00241188 | 1.80E-16 | 0.791854 | 150530196 | 0.000153761 | 66.97533662 |
|  | rs7797661 | T | C | 7 | 435516 | 0.0132886 | 0.00202599 | 3.60E-11 | 0.629453 | 24006788 | 9.87727E-05 | 43.02113547 |
|  | rs78089241 | G | A | 11 | 435516 | 0.0374832 | 0.00555678 | 1.80E-11 | 0.0327017 | 48214064 | 0.000104467 | 45.50141686 |
|  | rs78357199 | T | C | 8 | 435516 | 0.034258 | 0.00493899 | 1.80E-12 | 0.0415618 | 53138151 | 0.000110458 | 48.11114823 |
|  | rs7846925 | C | T | 9 | 435516 | 0.0218276 | 0.00293837 | 2.50E-14 | 0.130449 | 86282649 | 0.000126689 | 55.18194653 |
|  | rs78659622 | C | T | 4 | 435516 | 0.0293359 | 0.00437531 | 5.00E-12 | 0.0532478 | 87605963 | 0.000103212 | 44.95512198 |
|  | rs78842967 | G | T | 16 | 435516 | -0.0226897 | 0.00322633 | 8.10E-12 | 0.103592 | 69087491 | 0.00011355 | 49.45815829 |
|  | rs79218426 | T | C | 12 | 435516 | 0.07343 | 0.00502251 | 3.70E-51 | 0.0396965 | 102889497 | 0.000490556 | 213.7486802 |
|  | rs794364 | G | A | 7 | 435516 | -0.0130278 | 0.00200104 | 1.10E-11 | 0.416813 | 75130594 | 9.7316E-05 | 42.38660483 |
|  | rs7952436 | T | C | 11 | 435516 | 0.0435131 | 0.00358516 | 3.60E-35 | 0.0819955 | 67024534 | 0.000338121 | 147.3061811 |
|  | rs798502 | C | A | 7 | 435516 | 0.019905 | 0.00214366 | 9.30E-21 | 0.296664 | 2789880 | 0.000197935 | 86.22050475 |
|  | rs7985107 | A | G | 13 | 435516 | -0.013198 | 0.00197673 | 8.90E-12 | 0.522708 | 107675363 | 0.000102346 | 44.57789396 |
|  | rs79880058 | G | A | 5 | 435516 | -0.0254567 | 0.00429237 | 5.10E-10 | 0.0552938 | 35170289 | 8.07552E-05 | 35.17287859 |
|  | rs8015400 | A | C | 14 | 435516 | 0.0175676 | 0.00211454 | 4.20E-17 | 0.677791 | 25930988 | 0.00015846 | 69.02256023 |
|  | rs80170948 | G | T | 5 | 435516 | 0.0356836 | 0.00483999 | 1.50E-14 | 0.0445435 | 64020316 | 0.000124793 | 54.35586381 |
|  | rs8017377 | A | G | 14 | 435516 | -0.016477 | 0.00203176 | 1.60E-17 | 0.469948 | 24883887 | 0.000150988 | 65.76721888 |
|  | rs8024939 | T | C | 15 | 435516 | 0.015039 | 0.00248928 | 2.70E-10 | 0.195341 | 79049237 | 8.38011E-05 | 36.49962686 |
|  | rs8028620 | T | C | 15 | 435516 | 0.0191364 | 0.00197683 | 3.60E-23 | 0.512974 | 99202875 | 0.000215122 | 93.70868418 |
|  | rs8065258 | T | C | 17 | 435516 | -0.0176581 | 0.00249336 | 2.20E-13 | 0.193387 | 55777447 | 0.00011515 | 50.15520081 |
|  | rs8068844 | C | T | 17 | 435516 | -0.0145539 | 0.00208191 | 2.30E-13 | 0.338802 | 40571284 | 0.000112197 | 48.86893519 |
|  | rs8076897 | C | T | 17 | 435516 | 0.0143947 | 0.00210565 | 5.60E-11 | 0.668153 | 1985050 | 0.000107296 | 46.733776 |
|  | rs8078723 | C | T | 17 | 435516 | -0.0238042 | 0.00202074 | 4.20E-34 | 0.388278 | 38166879 | 0.000318525 | 138.7663963 |
|  | rs8104929 | T | C | 19 | 435516 | 0.0117995 | 0.00206612 | 7.90E-09 | 0.351435 | 22375868 | 7.48824E-05 | 32.61475582 |
|  | rs8105174 | T | C | 19 | 435516 | -0.0487349 | 0.00252451 | 6.60E-85 | 0.18742 | 10347032 | 0.000854969 | 372.6696053 |
|  | rs8121509 | C | T | 20 | 435516 | 0.0273344 | 0.00198316 | 1.70E-44 | 0.452565 | 62712053 | 0.000436024 | 189.9772462 |
|  | rs813139 | G | A | 4 | 435516 | 0.0154585 | 0.00198585 | 1.70E-16 | 0.431791 | 145858473 | 0.000139116 | 60.59542331 |
|  | rs823094 | G | T | 1 | 435516 | -0.0148215 | 0.00198664 | 3.30E-14 | 0.422289 | 205689807 | 0.000127787 | 55.66009859 |
|  | rs828617 | T | G | 3 | 435516 | -0.0173991 | 0.00204502 | 8.90E-19 | 0.377476 | 98541422 | 0.000166181 | 72.3863127 |
|  | rs838133 | G | A | 19 | 435516 | 0.0278368 | 0.00204374 | 2.10E-44 | 0.542366 | 49259529 | 0.000425793 | 185.5176925 |
|  | rs861525 | T | C | 12 | 435516 | 0.0145315 | 0.0022191 | 1.20E-11 | 0.734427 | 33726340 | 9.8451E-05 | 42.88102434 |
|  | rs867264 | A | G | 20 | 435516 | 0.0130951 | 0.00198333 | 5.40E-11 | 0.551949 | 45760490 | 0.000100088 | 43.59389581 |
|  | rs887506 | G | A | 14 | 435516 | -0.0154127 | 0.00202772 | 1.70E-14 | 0.392392 | 74219367 | 0.000132642 | 57.77493778 |
|  | rs898962 | G | A | 16 | 435516 | -0.0131612 | 0.0020038 | 6.70E-12 | 0.600905 | 81497952 | 9.90456E-05 | 43.14000972 |
|  | rs901105 | C | A | 11 | 435516 | -0.0242322 | 0.00266079 | 1.40E-20 | 0.163715 | 77924607 | 0.000190405 | 82.93970681 |
|  | rs910633 | G | A | 1 | 435516 | -0.0124239 | 0.00195449 | 1.70E-10 | 0.512883 | 101749974 | 9.27694E-05 | 40.40610558 |
|  | rs9379084 | A | G | 6 | 435516 | 0.0212594 | 0.00313384 | 3.30E-12 | 0.116526 | 7231843 | 0.000105657 | 46.01997445 |
|  | rs9427104 | T | C | 1 | 435516 | -0.0217109 | 0.00195423 | 1.90E-29 | 0.47867 | 154589232 | 0.00028332 | 123.4247646 |
|  | rs943513 | A | G | 1 | 435516 | 0.0254815 | 0.00202406 | 9.40E-39 | 0.376201 | 44035472 | 0.000363782 | 158.4897646 |
|  | rs9465733 | C | A | 6 | 435516 | 0.0116816 | 0.00208299 | 1.10E-08 | 0.666361 | 20409775 | 7.22096E-05 | 31.45055385 |
|  | rs9533031 | T | G | 13 | 435516 | 0.0126855 | 0.00200684 | 5.20E-11 | 0.573951 | 42772717 | 9.17373E-05 | 39.95652285 |
|  | rs9534448 | A | G | 13 | 435516 | 0.0193792 | 0.0023102 | 3.10E-17 | 0.761137 | 47212961 | 0.000161547 | 70.36724309 |
|  | rs9549092 | C | T | 13 | 435516 | 0.0398124 | 0.00256305 | 4.90E-58 | 0.180976 | 40754324 | 0.000553704 | 241.2795789 |
|  | rs9578326 | T | G | 13 | 435516 | -0.0185832 | 0.00236144 | 4.50E-15 | 0.226523 | 21487599 | 0.000142174 | 61.92773225 |
|  | rs9604573 | A | G | 13 | 435516 | 0.0200385 | 0.00227968 | 1.20E-17 | 0.256471 | 114542858 | 0.000177379 | 77.26461505 |
|  | rs9657541 | T | C | 8 | 435516 | -0.018358 | 0.00255351 | 5.90E-13 | 0.204789 | 10643164 | 0.000118664 | 51.68608182 |
|  | rs9687846 | A | G | 5 | 435516 | 0.0141287 | 0.0024503 | 6.60E-10 | 0.200637 | 55861894 | 7.63359E-05 | 33.24787499 |
|  | rs976002 | G | A | 4 | 435516 | -0.0363152 | 0.00231206 | 1.60E-55 | 0.243704 | 69343287 | 0.000566146 | 246.704281 |
|  | rs9813894 | A | G | 3 | 435516 | 0.0298518 | 0.00271765 | 3.70E-29 | 0.843124 | 88188420 | 0.000276968 | 120.6568558 |
|  | rs9833810 | A | C | 3 | 435516 | -0.0133955 | 0.00208821 | 5.80E-11 | 0.329644 | 113363762 | 9.44766E-05 | 41.14978028 |
|  | rs985296 | A | G | 5 | 435516 | -0.0219062 | 0.00201674 | 8.60E-30 | 0.386007 | 88377644 | 0.00027084 | 117.9864891 |
|  | rs9859077 | C | G | 3 | 435516 | 0.0272236 | 0.00210416 | 3.70E-41 | 0.33181 | 101136402 | 0.000384205 | 167.3908038 |
