# Supplementary material for: Tetrandrine, a Major Alkaloid From Stephaniae Tetrandrae Radix, Ameliorates Non‐Alcoholic Fatty Liver Disease in Zebrafish via the PI3K/AKT/STAT3 Pathway
Source: Food Sci Nutr. 2026 May 12;14(5):e71814. doi: 10.1002/fsn3.71814 (PMC13168532; doi:10.1002/fsn3.71814)
Supplement: Supplementary file 10 — Table S9: Results of MR analysis of feature genes and NAFLD. [file FSN3-14-e71814-s015.docx]

**Table S9 Results of MR Analysis of feature genes and NAFLD**

| Gene | ID number | MR Methods | N SNPs | OR (95%CI) | *P* value |
| --- | --- | --- | --- | --- | --- |
| TP53 | eqtl-a-ENSG00000141510 | Inverse variance weighted | 2 | 0.96(0.68,1.35) | 0.81 |
|  |  | MR Egger | 7 | 0.81(0.56,1.17) | 0.31 |
|  |  | Weighted median | 7 | 0.82(0.70,0.96) | 0.02 |
| STAT3 | eqtl-a-ENSG00000168610 | Inverse variance weighted | 7 | 0.85(0.74,0.97) | 0.02 |
|  |  | Simple mode | 7 | 0.84(0.67,1.05) | 0.17 |
|  |  | Weighted mode | 7 | 0.82(0.69,0.97) | 0.06 |
|  |  | MR Egger | 21 | 1.08(0.85,1.37) | 0.55 |
|  |  | Weighted median | 21 | 1.01(0.90,1.13) | 0.86 |
| EGFR | prot-a-909 | Inverse variance weighted | 21 | 0.96(0.88,1.05) | 0.37 |
|  |  | Simple mode | 21 | 1.08(0.87,1.34) | 0.51 |
|  |  | Weighted mode | 21 | 1.08(0.87,1.33) | 0.50 |
|  |  | MR Egger | 9 | 1.08(0.92,1.26) | 0.40 |
|  |  | Weighted median | 9 | 1.08(0.97,1.21) | 0.16 |
| AKT1 | eqtl-a-ENSG00000142208 | Inverse variance weighted | 9 | 1.04(0.95,1.15) | 0.38 |
|  |  | Simple mode | 9 | 1.02(0.83,1.26) | 0.87 |
|  |  | Weighted mode | 9 | 1.08(0.96,1.20) | 0.24 |
|  |  | MR Egger | 6 | 1.15(0.92,1.43) | 0.29 |
|  |  | Weighted median | 6 | 1.02(0.90,1.16) | 0.79 |
| TNF | eqtl-a-ENSG00000232810 | Inverse variance weighted | 6 | 0.99(0.87,1.13) | 0.91 |
|  |  | Simple mode | 6 | 0.88(0.63,1.22) | 0.47 |
|  |  | Weighted mode | 6 | 1.03(0.90,1.18) | 0.64 |
|  |  | MR Egger | 9 | 0.98(0.83,1.16) | 0.81 |
|  |  | Weighted median | 9 | 1.00(0.91,1.11) | 0.95 |
| CTNNB1 | eqtl-a-ENSG00000168036 | Inverse variance weighted | 9 | 1.01(0.93,1.10) | 0.82 |
|  |  | Simple mode | 9 | 1.08(0.87,1.36) | 0.50 |
|  |  | Weighted mode | 9 | 0.99(0.89,1.10) | 0.91 |
|  |  | MR Egger | 7 | 0.54(0.26,1.13) | 0.16 |
|  |  | Weighted median | 7 | 1.19(0.88,1.61) | 0.27 |
| BCL2 | eqtl-a-ENSG00000171791 | Inverse variance weighted | 7 | 1.12(0.88,1.42) | 0.38 |
|  |  | Simple mode | 7 | 1.23(0.75,2.00) | 0.44 |
|  |  | Weighted mode | 7 | 1.24(0.80,1.94) | 0.37 |
|  |  | MR Egger | 386 | 0.85(0.68,1.05) | 0.13 |
|  |  | Weighted median | 386 | 0.93(0.87,1.11) | 0.43 |
| INS | ebi-a-GCST90025989 | Inverse variance weighted | 386 | 0.97(0.87,1.09) | 0.62 |
|  |  | Simple mode | 386 | 0.91(0.57,1.43) | 0.67 |
|  |  | Weighted mode | 386 | 0.91(0.73,1.12) | 0.35 |
