# Supplementary material for: Tetrandrine, a Major Alkaloid From Stephaniae Tetrandrae Radix, Ameliorates Non‐Alcoholic Fatty Liver Disease in Zebrafish via the PI3K/AKT/STAT3 Pathway
Source: Food Sci Nutr. 2026 May 12;14(5):e71814. doi: 10.1002/fsn3.71814 (PMC13168532; doi:10.1002/fsn3.71814)
Supplement: Supplementary file 11 — Table S10: Heterogeneity and pleiotropy of MR analysis results. [file FSN3-14-e71814-s014.docx]

**Table S10 Heterogeneity and Pleiotropy of MR Analysis results**

|  | Heterogeneity | | | | Pleiotropy | | MR-PRESSO | |
| --- | --- | --- | --- | --- | --- | --- | --- | --- |
| Gene | MR Egger | | IVW | | MR Egger | | Global Test | |
|  | Cochran’s Q | *P-*value | Cochran’s Q | *P*-value | Egger intercept | *P*-value | RSSobs | *P*-value |
| TP53 |  |  | 0.84 | 0.36 |  |  |  |  |
| STAT3 | 3.27 | 0.66 | 3.35 | 0.76 | 0.01 | 0.79 | 4.53 | 0.827 |
| EGFR | 25.50 | 0.14 | 26.87 | 0.14 | -0.02 | 0.32 | 29.58 | 0.143 |
| AKT1 | 1.85 | 0.97 | 2.08 | 0.98 | -0.007 | 0.65 | 5.44 | 0.877 |
| TNF | 0.67 | 0.95 | 3.10 | 0.68 | -0.028 | 0.19 | 14.12 | 0.511 |
| CTNNB1 | 6.34 | 0.50 | 6.54 | 0.59 | 0.009 | 0.67 | 9.19 | 0.624 |
| BCL2 | 3.09 | 0.69 | 7.18 | 0.30 | 0.067 | 0.09 | 16.49 | 0.112 |
| INS | 525.82 | 1.92E-06 | 528.80 | 1.49E-06 | 0.004 | 0.14 | 538.60 | <0.001 |
